# Downregulated PDIA3P1 lncRNA Impairs Trophoblast Phenotype by Regulating Snail and SFRP1 in PE

**DOI:** 10.1155/2024/8972022

**Published:** 2024-04-27

**Authors:** Zhengzheng Ding, Liuxin Wu, Yue Sun, Yuanyuan Zhu, Qing Zuo, Li Yuan, Cong Wang, Lizhou Sun, Yetao Xu, Yuanyuan Zhang

**Affiliations:** ^1^Department of Obstetrics and Gynecology, First Affiliated Hospital of Nanjing Medical University, Nanjing 210029, Jiangsu, China; ^2^Department of Obstetrics and Gynecology, Nanjing Maternity and Child Health Care Hospital, Women' s Hospital of Nanjing Medical University, 123 Tianfeixiang, Mochou Road, Qinhuai District, Nanjing 210004, Jiangsu, China; ^3^Department of Obstetrics and Gynecology, Taizhou Maternity Hospital Affiliated to Nantong University, Taizhou 225300, Jiangsu, China

## Abstract

Preeclampsia (PE) manifests as a pregnancy-specific complication arising from compromised placentation characterized by inadequate trophoblast invasion. A growing body of evidence underscores the pivotal involvement of pseudogenes, a subset of long noncoding RNAs, in the pathological processes of PE. This study presents a novel finding, demonstrating a significant downregulation of the pseudogene PDIA3P1 in PE placental tissues compared to normal tissues. In vitro functional assays revealed that suppressing PDIA3P1 hindered trophoblast proliferation, invasion, and migration, concurrently upregulating the expression of secreted frizzled-related protein 1 (SFRP1). Further exploration of the regulatory role of PDIA3P1 in PE, utilizing human trophoblasts, established that PDIA3P1 exerts its function by binding to HuR, thereby enhancing the stability of Snail expression in trophoblasts. Overall, our findings suggest a crucial role for PDIA3P1 in regulating trophoblast properties and contributing to the pathogenesis of PE, offering potential targets for prognosis and therapeutic intervention.

## 1. Background

Preeclampsia (PE), marked by hypertension onset coupled with proteinuria (>300 mg/day) after 20 weeks of gestation in normotensive women [1], affects 2%–8% of pregnancies and remains a primary cause of global maternal and perinatal morbidity and mortality [2]. PE leads to significant acute and long-term complications, including eclampsia, stroke, liver rupture, pulmonary edema, and renal failure, with potential lethality [3]. Additionally, PE correlates closely with fetal growth restriction and preterm birth, either spontaneous or through iatrogenic delivery. Presently, apart from delivery, effective clinical methods for PE control are lacking [4, 5]. Consequently, global researchers emphasize enhancing clinical and basic PE research to improve perinatal outcomes.

Various preliminary theories have been posited, with the two-stage theory being widely acknowledged [6]. This theory divides PE occurrence and development into abnormal placentation and maternal syndrome development [7]. In a normal pregnancy, uterine spiral artery remodeling occurs moderately [8], with cytotrophoblasts invading maternal spiral artery arterioles and remodeling them to form low-resistance, high-bored blood vessels, ensuring sufficient oxygen, and nutrient supply to the fetus [9, 10]. In PE, cytotrophoblasts fail to invade the myometrium, restricting spiral arteries to the decidua, causing incomplete placental perfusion, and releasing hypoxia-inducible factors into the peripheral blood [11, 12]. In essence, the placenta's pivotal role in PE pathogenesis is initiated by placental ischemia and the subsequent release of antiangiogenic factors into circulation [13]. Despite some progress in PE pathogenesis research, deeper underlying mechanisms necessitate further exploration and elucidation.

The evolution of high-throughput technologies in recent decades has facilitated a comprehensive exploration of the noncoding genome at an unprecedented resolution and scale [14]. Concurrently, numerous long noncoding RNAs (lncRNAs) have surfaced through functional genomics studies [15]. LncRNAs, transcripts exceeding 200 nucleotides that do not undergo protein translation, surprisingly emerge as master regulators of gene expression, exerting a crucial regulatory role in biological processes, notably in the onset and progression of PE [16, 17]. For example, the PVT1 lncRNA modulates cell migration, invasion, and proliferation by hindering ANGPTL4 transcription through its binding to EZH2 in trophoblasts [18]. The downregulation of HOXA11-AS in placental tissues from PE patients is associated with the regulation of trophoblast migration and invasion through the HOXA11-AS/MiR-15b-5p/HOXA7 pathway [19].

Pseudogenes, representing defective gene copies, number over 14,000 in the human genome [20]. Recent research exploring the connection between pseudogenes and cancer progression discovered 1970 novel peptides from pseudogenes in tumor tissues, including breast cancer, lung cancer, and colorectal cancer [21]. Pseudogene-expressed RNAs constitute crucial components of lncRNAs [22]. Pseudogenes, a subclass of lncRNAs originating from protein-coding genes that have lost their protein-producing capability, are poorly understood in terms of their underlying regulatory mechanisms in PE [23–25].

In this study, we identified a novel pseudogene (PDIA3P1) as a significant regulator promoting trophoblast proliferation and migration. Given the scarcity of studies on the biological functions and molecular mechanisms of PDIA3P1 in PE, we undertook the following investigations. Initially, we observed a significant downregulation of PDIA3P1 in the placental tissues of PE patients compared to normal placental tissues. In vitro functional assays demonstrated that PDIA3P1 knockdown upregulated the expression of secreted frizzled-related protein 1 (SFRP1), exerting an inhibitory effect on the Wnt/*β*-catenin signaling pathway and thereby restraining trophoblast proliferation, invasion, and migration. Additionally, PDIA3P1 stabilized Snail mRNA expression and enhanced Snail expression by binding to HuR. Overall, our study advances the understanding of the mechanism through which PDIA3P1 regulates trophoblast properties and contributes to the pathogenesis of PE.

## 2. Materials and Methods

### 2.1. Methods

#### 2.1.1. Criteria for PE Patients

Preeclampsia diagnosis is established in women exhibiting new-onset hypertension (systolic blood pressure ≥ 140 mmHg and/or diastolic blood pressure ≥ 90 mmHg) accompanied by proteinuria (≥0.3 g/24 hr or random urine protein (+)) or other indicators of severe organ dysfunction in the latter half of pregnancy. Severe preeclampsia can be diagnosed in patients presenting with any of the following adverse conditions: (1) escalating blood pressure with systolic pressure exceeding 160 mmHg and/or diastolic pressure equal to or greater than 110 mmHg; (2) proteinuria surpassing 5.0 g/24 hr or random urine protein (++); (3) serum creatinine exceeding 1.2 mg/dl; (4) thrombocytopenia, blood platelet count <100,000/ML (<100 × 10^9^/L); (5) elevated microangiopathic hemolytic LDH; (6) impaired liver function indicated by elevated serum transaminase levels-ALT or AST; (7) persistent headache or other brain or visual disorders; (8) prolonged abdominal pain duration; (9) heart failure, pulmonary edema; (10) fetal growth restriction or oligohydramnios; (11) progressive renal insufficiency; and (12) new-onset cerebral or visual disturbances.

#### 2.1.2. Collection of Patient Samples

A total of 48 placental tissue samples were procured from the Obstetrical Department of the First Affiliated Hospital of Nanjing Medical University between December 2018 and May 2020. Placenta tissue samples (approximately 1 cm × 1 cm × 1 cm) were extracted from the central area of the placenta maternal surface at four different locations within 5 min postdelivery, avoiding necrotic and calcified regions. These samples were placed in embedding molds containing optimal cutting temperature (OCT) medium, frozen over a dry ice/ethanol slurry, and stored at −80°C for subsequent RNA and protein extraction. Patient clinical characteristics were analyzed, as presented in *Supplementary 1*.

#### 2.1.3. Cell Culture

HTR-8/SVneo, JAR, and HUVEC cell lines were procured from the Type Culture Collection of the Chinese Academy of Sciences (Shanghai, China). HTR-8/SVneo cells were sustained in RPMI-1640 medium (Gibco, Grand Island, NY, USA), while the other cell lines were maintained in DMEM (Gibco) supplemented with 10% fetal bovine serum and 1% penicillin–streptomycin. Cells were cultured in a standard humidified atmosphere of 5% CO_2_ at 37°C, with fresh medium replenished every 2 days.

Plasmid construction, siRNA, and cell transfection: invitrogen (Shanghai, China) provided siRNAs. The full-length cDNA of human PDIA3P1 was synthesized and cloned into an expression vector by RiboBio (Guangzhou, China), with verification through sequencing. PDIA3P1 was introduced into the pEXP-RB-Mam-EGFP empty vector using EcoRI and XhoI restriction sites. Following plasmid transformation, ampicillin-resistance genes were employed for screening, and monoclonal colonies were selected for culture. Plasmids were prepared using the Endo-Free Maxi Plasmid Kit (TIANGEN, China). Cells underwent transfection with siRNA or plasmids using Lipofectamine™ 3000 (Invitrogen, Carlsbad, CA, USA) following the manufacturer's instructions. *Supplementary 2* lists all sequences.

#### 2.1.4. RNA Extraction and qPCR Analysis

Total RNA from tissues and cultured cells was extracted using TRIZOL reagent (Invitrogen). Subsequently, this RNA underwent reverse transcription into cDNA using a Reverse Transcription Kit (Takara, Dalian, China). All PCR amplifications were conducted in triplicates for each cDNA sample on the Step OnePlus System (Applied Biosystems). Glyceraldehyde 3-phosphate dehydrogenase (GAPDH) served as the internal control for comparison. *Supplementary 3* lists all primer sequences employed in this study.

#### 2.1.5. Cell Proliferation Assays

In the Cell Counting Kit-8 (CCK-8) assay, cells were seeded at a density of 5,000 cells/well in 96-well plates. Cell proliferation was gauged using the CCK-8 Kit (Vazyme, Nanjing, China) following the manufacturer's instructions. After a 2-hr incubation under culture conditions, microplate readers measured absorbance at 450 nm. EdU experiments adhered to the manufacturer's protocol for the EdU labeling/detection kit (RiboBio, Guangzhou, China). Treated cells were introduced to 50 *μ*M EdU-labeling medium and incubated for 2 hr, followed by fixation with 4% paraformaldehyde and permeation with 0.5% Triton X-100. Subsequently, cells were treated with 1 × Apollo reaction solution for 30 min and DAPI staining solution for 5 min. EdU-positive cells were observed and counted under a fluorescence microscope. The colony formation assay involved seeding a specified number of transfected cells into six-well plates, maintaining them in suitable media for 12 days with medium replacement every 4 days. Colonies were fixed with 4% paraformaldehyde for 20 min and stained with 0.1% crystal violet for 20 min. Visible colonies were photographed and counted.

Flow cytometric analysis of cell cycle: For cell cycle analysis, cells underwent incubation using the PI/RNase Staining Kit (KeyGEN, China). The staining buffer of PI/RNase A was prepared at a 9 : 1 dilution. Cells were harvested, suspended in 500 mL of 70% cold ethanol, and fixed overnight at 4°C. After washing with PBS, cell pellets were incubated with the staining solution in the dark for 30 min. Flow cytometry (BD FACScan; BD Biosciences) estimated the percentage of cells in the G0/G1, S, and G2/M phases.

#### 2.1.6. Cell Migration and Invasion Assays

Transwell assays, conducted in 24-well transwell chambers with 8-mm pore-size polycarbonate membranes (Corning, USA), were employed to detect cell migration and invasion. Medium without FBS (4 × 10^4^ cells/200 *μ*L) was added to the upper membrane portion coated with/without Matrigel (BD, USA), and 500 *μ*L medium containing 10% FBS was added to the lower chamber. After 24 hr for migration or 48 hr for invasion, chambers were fixed with methanol for 30 min and stained with 0.1% crystal violet solution for 20 min. Positive cells penetrating the filter membrane were observed and photographed under the microscope.

#### 2.1.7. Network Formation Assay

HUVEC cells underwent network formation assays following the method reported by Xu et al. [26]. Transfected cells with siRNA or plasmid (5 × 10^4^ cells/well) were cultured in 96-well plates with five duplicates. After 6 hr, images were captured under the microscope, and the Angiogenesis Analyzer, developed for ImageJ software, was used to analyze the number of branches and total branch length in each field. Results were derived from three repeated experiments.

#### 2.1.8. RNA-Sequencing Analysis

RNA-sequencing (RNA-seq) experiments were conducted by Applied Protein Technology (Shanghai, China), with mRNA-seq libraries established according to Illumina standard protocols (San Diego, CA, USA). Total RNA from siNC and siPDIA3P1-1#-transfected HTR-8/SVneo cells was extracted, reverse-transcribed into cDNA, and fragmented via nebulization to establish the mRNA-seq library. Differentially expressed genes were identified by comparing siRNAs with control DESeq2, with fold changes of ≥2.0 set as the cut-off values for aberrant mRNA expression. *Supplementary 4* lists the analysis of RNA transcriptome sequencing data in this study.

#### 2.1.9. In Vitro Transcription Assays and RNA Pull-Down Mass Spectrometry (LC-MS/MS) Assays

In RNA pull-down assays, PDIA3P1 RNA and its antisense RNA were transcribed and biotinylated in vitro using a DNA template with the T7 promoter, following the Ribo™ RNAmax-T7 Biotin Labeling Transcription Kit instructions (Cat. AM1344, Invitrogen, CA, USA). Biotinylated RNA was captured using streptavidin magnetic beads and then incubated with HTR-8/SVneo cell extract. The eluted protein from the RNA–protein complex underwent sodium dodecyl sulfate–polyacrylamide gel electrophoresis (SDS-PAGE), western blotting using HuR antibody, and staining with silver staining solution (Beyotime, China) for mass spectrometry. After elution of lncRNA-interacting proteins, mass spectrometric analysis was performed using an LTQ linear ion trap mass spectrometer (Thermo Finnigan, San Jose, CA). The detailed process of the RNA pull-down assay is listed in *Supplementary 5*.

#### 2.1.10. Fluorescence In Situ Hybridization

A PDIA3P1-specific probe designed and synthesized by RiboBio (China) was used for fluorescence in situ hybridization (FISH). The localization and distribution of PDIA3P1 were detected using an FISH kit according to the manufacturer's instructions. 18S RNA and U6 served as controls to indicate the cytoplasm and nucleus, respectively.

Subcellular dractionation: Nuclear and cytosolic fractions were separated using the PARIS Kit (Life Technologies) following the manufacturer's instructions. U1 and GAPDH were used as nucleus and cytoplasm controls, respectively, for comparison. PDIA3P1, GAPDH, and U1 levels were detected using RT-PCR.

#### 2.1.11. RNA Immunoprecipitation Analysis

RNA immunoprecipitation (RIP) assays were executed using the Millipore Magna RIP Kit (Millipore) in adherence to the manufacturer's instructions. HTR-8/SVneo cells, harvested at a density of 80%–90% in a 15 cm diameter cell culture dish, underwent lysis with RIP lysis buffer. Subsequently, cell lysates were incubated overnight at 4°C with magnetic beads conjugated with control IgG and HuR antibodies. Following washing, the beads were incubated with proteinase K to isolate the immunoprecipitated RNA, which was then reverse transcribed into cDNA and analyzed using qPCR.

#### 2.1.12. Immunohistochemistry Analysis

Immunohistochemistry (IHC) analysis assessed SFRP1 protein expression in placental tissue. The anti-SFRP1 antibody for IHC was procured from Abcam (Cambridge, UK). Placental tissues from PE patients and normal pregnant women were embedded in paraffin, following conventional treatment, and subjected to IHC analysis.

#### 2.1.13. Western Blot Analysis

Western blotting, conducted according to standard methods, aimed to detect proteins. Proteins extracted from cells or tissues treated with RIPA lysis buffer (Beyotime, China) supplemented with protease inhibitor cocktail and PMSF (Roche) were quantified using the BCA Kit (Beyotime, China). Subsequently, protein samples were separated using SDS-PAGE, and proteins on the gel were transferred onto PVDF membranes (Bio-Rad). Overnight incubation at 4°C followed with the corresponding primary antibodies (1 : 1,000): HuR, GAPDH (Abcam, Cambridge, UK), E-cadherin, N-cadherin, Snail, and *β*-catenin (Cell Signaling Technology, MA, USA). HRP-conjugated sheep antirabbit IgG secondary antibody (1 : 10,000) from Cell Signaling Technology was employed. Protein signals were detected using ECL western blotting substrate (Thermo Fisher, USA), and band intensity was quantified using the Quantity One software (Bio-Rad). The Uncut western gel images are listed in *Supplementary 6*.

#### 2.1.14. Statistical Analyses

SPSS 20.0 (IBM) was utilized for statistical analyses, and data were expressed as mean ± SD of three independently repeated experiments. Comparison between two independent groups was executed using Student's *t*-test, with statistical significance considered at *p* values of <0.05 or <0.01.

## 3. Results

### 3.1. PDIA3P1 Expression Is Significantly Downregulated in the Placental Tissue of Patients with PE

**Figure 1:**
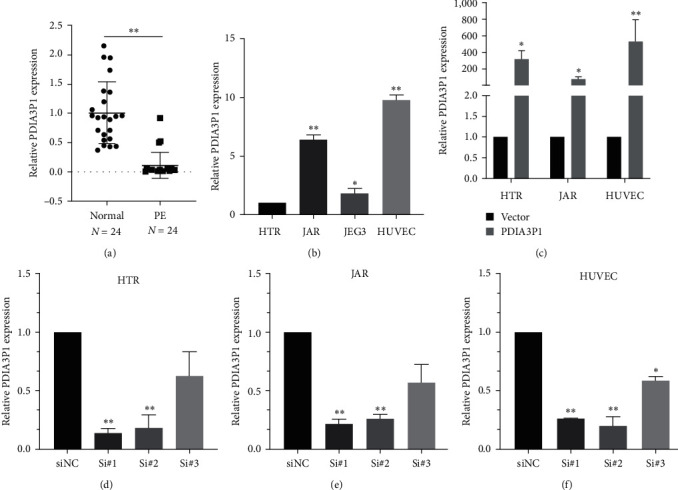
PDIA3P1 downregulation in PE placental tissues. (a) RT-qPCR analysis revealed diminished PDIA3P1 expression in PE placental tissues compared to normal tissues. (b) PDIA3P1 expression levels were assessed in trophoblast cell lines (HTR-8/SVneo, JAR, and JEG3) and the HUVEC cell line. (c) RT-qPCR analysis depicted PDIA3P1 expression in HTR-8/SVneo, JAR, and HUVEC cells transfected with a PDIA3P1-overexpressing plasmid. Relative PDIA3P1 expression in HTR-8/SVneo (d), JAR (e), and HUVEC (f) cells after PDIA3P1 siRNA transfection. Data are presented as mean ± SD.  ^*∗*^*p*  < 0.05;  ^*∗∗*^*p*  < 0.01.

As indicated in *Supplementary 1*, patients with PE exhibited significantly elevated systolic and diastolic blood pressures, as well as proteinuria levels, compared to normal pregnant women. Conversely, neonatal body weight was noticeably lower in PE pregnancies. To further validate the expression of PDIA3P1, we conducted qPCR assays on 24 pairs of PE and normal placental tissues. PDIA3P1 expression in PE placental tissues was significantly lower than in normal placental tissues (*p*  < 0.05) (Figure 1(a)).

Subsequent examinations assessed PDIA3P1 expression in trophoblast cell lines (HTR-8/SVneo, JAR, and JEG3) and the HUVEC cell line, as depicted in Figure 1(b). We chose the normal trophoblast cell line HTR-8/SVneo, the JAR cell line exhibiting relatively high PDIA3P1 expression, and the HUVEC cell line to investigate PDIA3P1′s functional activity. Results demonstrated ectopic overexpression of PDIA3P1 with the pEGFP-PDIA3P1 vector in the selected cell lines (Figure 1(c)). Additionally, three independent siRNAs (1#, 2#, and 3#) were transfected into the cell lines to downregulate PDIA3P1 expression. As illustrated in Figure 1(d)–1(f), siRNAs 1# and 2# more effectively reduced PDIA3P1 expression, and they were chosen for subsequent functional experiments. Overall, these findings indicate that the diminished expression of PDIA3P1 may hold crucial clinical significance in the onset and progression of PE.

### 3.2. Reduced Expression of PDIA3P1 Affects Trophoblast Proliferation and Induces Cell Cycle Arrest

**Figure 2:**
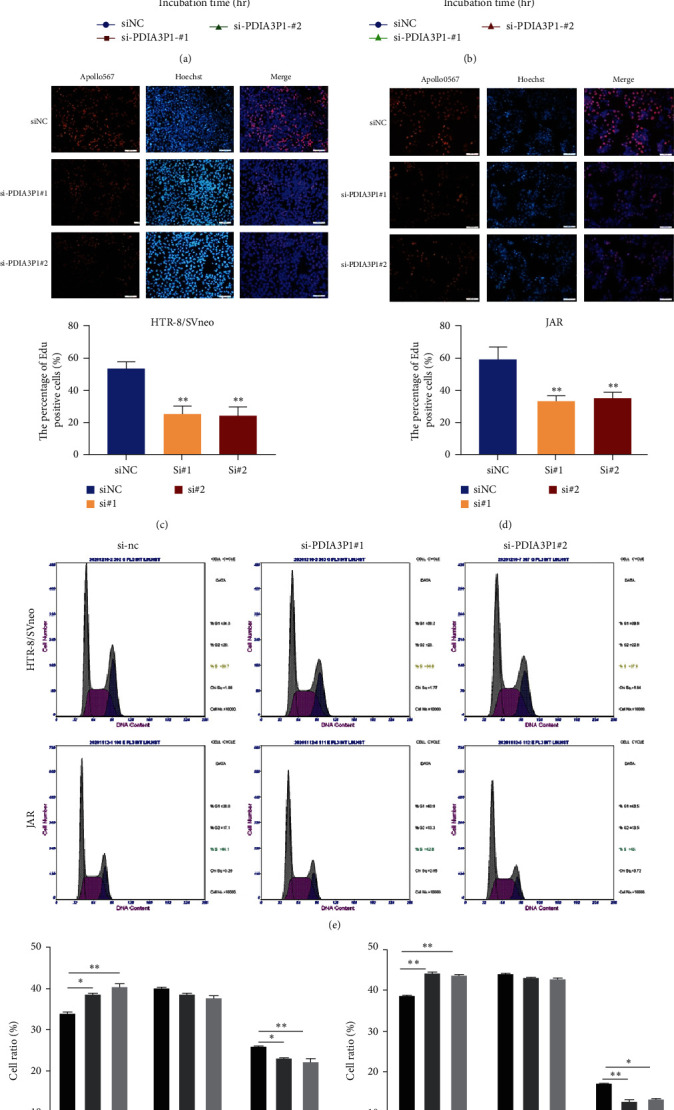
PDIA3P1 depletion and trophoblast proliferation. (a) Validation of cell proliferation viability in si-PDIA3P1-transfected HTR-8/SVneo cells. (b) CCK-8 assays determined the proliferation of JAR cells post PDIA3P1 siRNA transfection. EdU assays illustrated the proliferation of HTR-8/SVneo (c) and JAR cells (d) following siPDIA3P1 transfection. (e) Flow cytometry evaluated the impact of si-PDIA3P1 on the cell cycle in HTR-8/SVneo and JAR cells. On the right is the quantification of cell cycle (f). Data are reported as mean ± SD.  ^*∗*^*p*  < 0.05;  ^*∗∗*^*p*  < 0.01.

To explore the biological implications of PDIA3P1 in trophoblasts, gain-of-function and loss-of-function experiments were conducted. The in vitro CCK-8 assay revealed a significant inhibition of proliferative activity in HTR-8/SVneo and JAR cells following PDIA3P1 knockdown (Figures 2(a) and 2(b)). Additionally, EdU staining confirmed the reduction in the proportion of EdU-positive cells in the interference group (Figures 2(c) and 2(d)). Conversely, PDIA3P1 overexpression markedly enhanced the proliferation of HTR-8/SVneo cells (*Supplementary 7*) and JAR cells (*Supplementary 7*). Collectively, these findings indicate that downregulation of PDIA3P1 expression adversely influences the proliferation of HTR/SVneo and JAR trophoblasts. Furthermore, we explored whether PDIA3P1-mediated proliferation involved cell cycle regulation in HTR-8/SVneo and JAR cells, as evidenced by flow cytometric analysis. PDIA3P1 knockdown induced cell cycle arrest in the G0/G1 phase (Figures 2(e) and 2(f)), while PDIA3P1 overexpression facilitated the transition from G0/G1 to S phase (*Supplementary 7*), aligning with the results of cell proliferation. These observations imply that PDIA3P1 participates in cell proliferation by regulating the trophoblast cell cycle progression.

### 3.3. Regulatory Role of PDIA3P1 in Trophoblast Migration, Invasion, and Tube Formation

**Figure 3:**
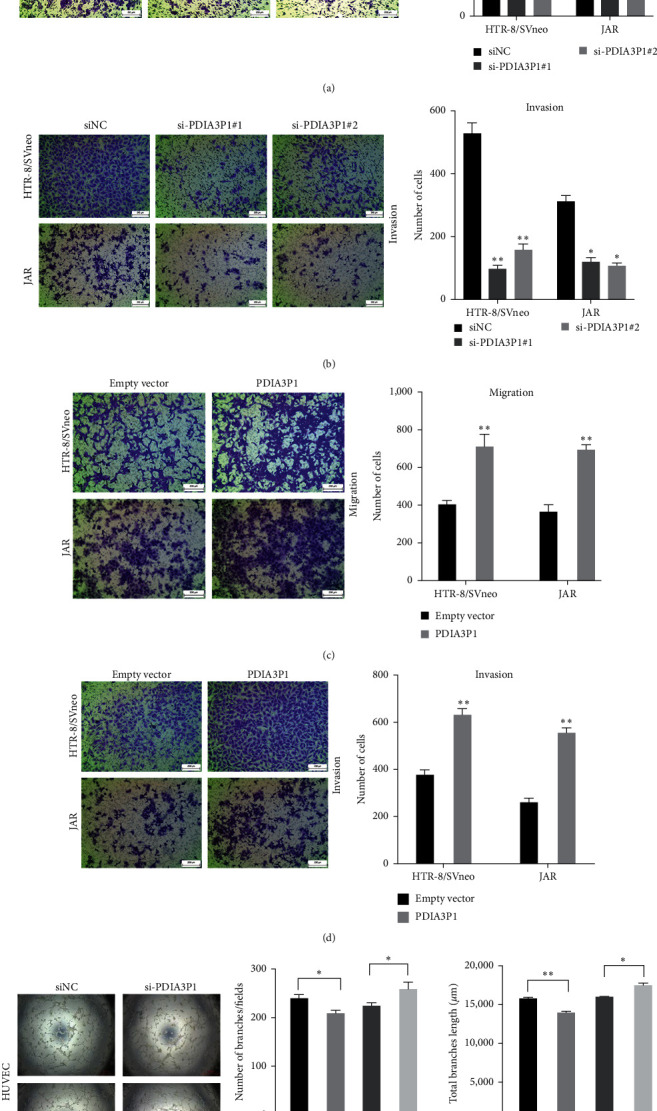
PDIA3P1 in trophoblast migration and invasion. (a) Transwell assay assessed the effects of siPDIA3P1 on HTR-8/SVneo and JAR cell migration, with quantification on the right. (b) Transwell assay evaluated the impact of siPDIA3P1 on HTR-8/SVneo and JAR cell invasion, with quantification on the right. (c and d) Cell migration (c) and invasion (d) were confirmed in HTR-8/SVneo and JAR cells transfected with a PDIA3P1-overexpressing plasmid. Quantification is provided below. PDIA3P1 expression was silenced or overexpressed in HUVECs. Performing network formation, cells transfected with siRNAs/overexpressed plasmid targeting PDIA3P1 showed a decrease/increase in node numbers as compared to the negative control. (e) Tube formation images of HUVEC cells transfected with siPDIA3P1 or PDIA3P1 plasmid were photographed. The number of branches (f) and total branch length (g) was analyzed through tube formation assays. Data are reported as mean ± SD.  ^*∗*^*p*  < 0.05;  ^*∗∗*^*p*  < 0.01.

Trophoblast migration and invasion are pivotal in placentation, and their dysregulation is implicated in the pathogenesis of PE. Transwell assays were conducted to assess whether heightened PDIA3P1 expression could drive cell migration. Intriguingly, PDIA3P1 depletion in human villous trophoblast cell lines, HTR-8/SVneo and JAR cells, substantially reduced the number of migrational cells (Figures 3(a) and 3(b)). Conversely, PDIA3P1 overexpression notably promoted the invasion and migration of HTR-8/SVneo and JAR cells (Figures 3(c) and 3(d)). The impact of PDIA3P1 on the in vitro network formation ability of HUVEC cells was also investigated. PDIA3P1 played a positive role in network formation, evident by a significant reduction in the number of branches and total branch length after PDIA3P1 siRNA transfection (Figures 3(e), 3(f), and 3(g)). Moreover, elevated PDIA3P1 expression markedly increased branch number and length compared with the empty vector (Figures 3(e), 3(f), and 3(g)).

### 3.4. SFRP1 Identified as a Principal Downstream Target of PDIA3P1

**Figure 4:**
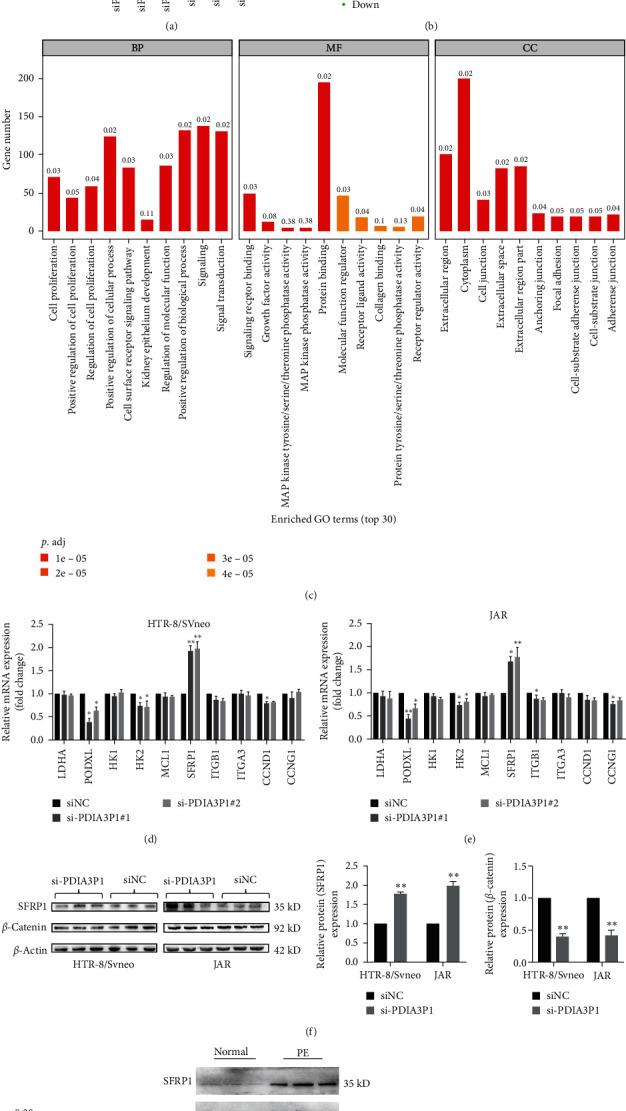
SFRP1 as a downstream target of PDIA3P1. (a, b) Identification of differentially expressed genes in siPDIA3P1-transfected HTR-8/SVneo cells through RNA-sequencing and gene expression profiling. (c) Gene ontology analysis revealed significant effects of PDIA3P1 depletion on cell growth and adhesion processes in trophoblasts. (d, e) RT-PCR confirmation of altered mRNA gene levels in PDIA3P1-deficient HTR-8/SVneo and JAR cells. (f) Western blot analysis showing changes in SFRP1 and *β*-catenin expression after siNC or si-PDIA3P1 transfection in HTR-8/SVneo and JAR cells, with *β*-actin as the internal control. (g) RT-qPCR analysis of SFRP1 mRNA expression in PE and normal placental tissues. (h) Western blot analysis of SFRP1 protein levels in PE and normal placental tissues, using GAPDH as a reference gene. (i) IHC demonstrates higher SFRP1 expression in PE placental tissues compared to normal tissues. Data are presented as mean ± SD.  ^*∗*^*p*  < 0.05;  ^*∗∗*^*p*  < 0.01.

Based on the RNA transcriptome sequencing data, significant alterations in gene transcript levels were observed in HTR/SVneo cells following PDIA3P1 knockdown (Figures 4(a) and 4(b)). Evaluation of biological pathways affected by PDIA3P1, using Gene Ontology and Kyoto Encyclopedia of Genes and Genomes databases, revealed changes in genes associated with cell motility and adhesion in PDIA3P1-depleted cells (Figure 4(c)), aligning with functional experiments. Subsequently, qPCR analysis confirmed a substantial upregulation of SFRP1 after PDIA3P1 knockdown in HTR-8/SVneo and JAR cells (Figures 4(d) and 4(e)), indicating SFRP1 as a crucial downstream mediator of PDIA3P1. Western blotting further validated a distinct increase in SFRP1 protein levels in PDIA3P1-depleted cells (Figure 4(f)). Notably, evaluation of SFRP1 expression in placental tissues, using qPCR, western blotting, and IHC staining, indicated a significant elevation in PE placental tissues (Figure 4(g)). Additionally, western blotting and IHC results demonstrated higher SFRP1 expression in PE placental tissues compared to normal placental tissues (Figures 4(h) and 4(i)). In summary, these findings establish a negative correlation between SFRP1 and PDIA3P1 expression levels in PE placental tissues.

### 3.5. SFRP1 Impact on Trophoblast Proliferation and Migration In Vitro and Its Mediation in PDIA3P1 Biological Function

**Figure 5:**
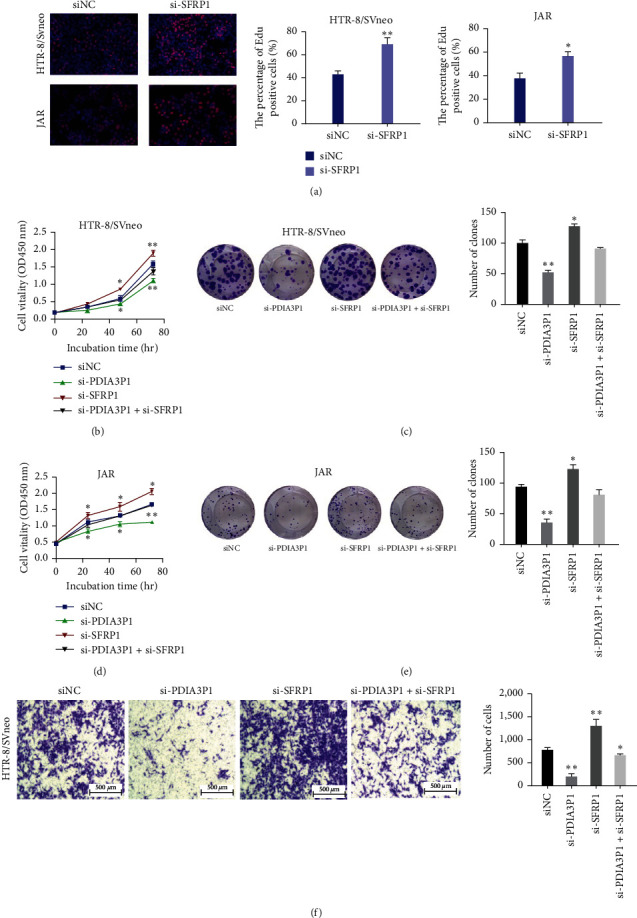
SFRP1′s impact on trophoblast proliferation and migration. (a) Validation of cell proliferation viability in si-SFRP1-transfected HTR-8/SVneo and JAR cells using EdU assay. (b) CCK-8 assays determine the proliferation ability of HTR-8/SVneo cells cotransfected with si-PDIA3P1 and si-SFRP1. (c) Colony formation assays assessing viability of si-PDIA3P1- and si-SFRP1-cotransfected HTR-8/SVneo cells. (d) Proliferation ability of JAR cells cotransfected with si-PDIA3P1 and si-SFRP1 analyzed by CCK-8 assays. (e) Colony formation assays determining viability of si-PDIA3P1- and si-SFRP1-cotransfected JAR cells. (f) Transwell assays investigating changes in the migration abilities of si-PDIA3P1- and si-SFRP1-cotransfected HTR-8/SVneo cells. Data are reported as mean ± SD.  ^*∗*^*p*  < 0.05;  ^*∗∗*^*p*  < 0.01.

We next ascertain the involvement of SFRP1 in the biological function of PDIA3P1 in trophoblasts. First, SFRP1 expression was silenced in HTR-8/SVneo and JAR cells more efficiently by si-SFRP1-1# and si-SFRP1-2# by analyzing quantitative RT-PCR. (*Supplementary 7*), then si-SFRP1 was transfected into HTR-8/SVneo and JAR trophoblasts, demonstrating enhanced cell proliferation in EdU assays (Figure 5(a)). Further, cotransfection with si-PDIA3P1 and si-SFRP1 in HTR-8/SVneo and JAR cells, assessed through CCK-8 and clone formation assays, revealed that si-SFRP1 could partially rescue the si-PDIA3P1-induced proliferation inhibition (Figures 5(b) and 5(c)–5(e)). Additionally, migration experiments with HTR-8/SVneo cells demonstrated that SFRP1 knockdown promoted migration and partially counteracted the migration inhibition induced by PDIA3P1 knockout (Figure 5(f)). In conclusion, these findings affirm the biological functional role of PDIA3P1 in trophoblasts, mediated by the inhibition of SFRP1 expression.

### 3.6. PDIA3P1 Involvement in Trophoblast Biological Functions through Snail Regulation

**Figure 6:**
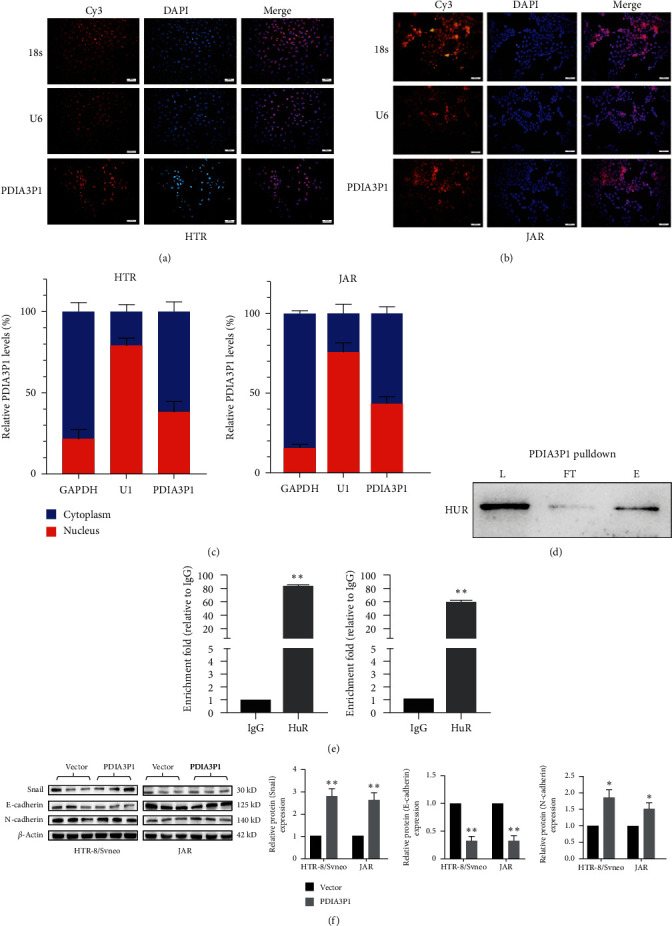
PDIA3P1′s involvement in trophoblast functions via HuR and Snail regulation. FISH analysis of PDIA3P1 distribution in HTR-8/SVneo (a) and JAR cells (b), indicating cytoplasmic localization. (c) Nucleocytoplasmic separation assay confirming subcellular localization of PDIA3P1 in HTR-8/SVneo and JAR cells. (d) RNA pull-down assay demonstrating the direct interaction between HuR and PDIA3P1 in HTR-8/SVneo cells. (e) RNA immunoprecipitation (RIP) experiments evaluating associations between PDIA3P1-HuR and HuR-Snail in HTR-8/SVneo cells. (f) Western blot analysis revealed changes in Snail, E-cadherin, and N-cadherin expression after PDIA3P1 overexpression in HTR-8/SVneo and JAR cells. Data are presented as mean ± SD.  ^*∗*^*p*  < 0.05;  ^*∗∗*^*p*  < 0.01.

The subcellular localization of PDIA3P1 is pivotal for deciphering its molecular mechanisms. Utilizing FISH in trophoblasts, we identified the primary cytoplasmic localization of PDIA3P1 (Figures 6(a) and 6(b)), indicating its engagement in posttranscriptional regulation. Subsequently, subcellular fractionation was employed to affirm PDIA3P1′s presence in both nuclear and cytoplasmic fractions of trophoblasts (Figure 6(c)), aligning with FISH results.

In pursuit of proteins binding to PDIA3P1, an RNA pull-down assay coupled with mass spectrometry identified HuR as a predominant binding partner. Subsequent RNA pull-down assays using biotinylated PDIA3P1 in HTR-8/SVneo cell lysates validated the specific interaction between HuR and PDIA3P1 (Figure 6(d)). Additionally, RIP assays demonstrated substantial PDIA3P1 enrichment by anti-HuR antibodies compared to nonspecific IgG control (Figure 6(e)). HuR, an RNA-binding protein known for stabilizing the mRNAs of MMPs and Snail, a prominent EMT-promoting transcription factor, was found to directly bind to Snail in HTR-8/SVneo cells (Figure 6(e)). Considering that Snail, E-cadherin, and N-cadherin are markers for pathways involved in cell invasion and migration, their protein levels were confirmed via western blotting. PDIA3P1 overexpression significantly upregulated Snail and N-cadherin protein expression, while E-cadherin expression markedly decreased (Figure 6(f)). These findings validate that PDIA3P1 can directly interact with HuR to modulate the expression of target genes, such as Snail, in trophoblasts.

## 4. Discussion

### 4.1. Trophoblast Behavior and PE Pathogenesis

During implantation, trophoblasts adhere to and subsequently invade the maternal endometrium, initiating decidualization and maternal spiral artery remodeling, ultimately forming the placenta [8]. Human placental development involves two primary trophoblast types: villus trophoblasts and extravillous trophoblasts (EVTs) [9]. EVT invasion is crucial for embryo implantation and placental formation. According to the classical perspective, impaired EVT invasion leads to inadequate uterine spiral artery remodeling, resulting in shallow placental implantation, compromised blood perfusion, ischemia, and other pathological changes, ultimately triggering PE [10]. A comprehensive understanding of factors governing trophoblast biological behavior and their regulatory mechanisms is pivotal for advancing the theoretical basis for PE diagnosis and treatment.

### 4.2. Pseudogene-Expressed RNAs and PDIA3P1 in PE

With advancements in high-throughput and genome sequencing technologies, exploration of lncRNAs' new biological functions in embryo development and tumorigenesis is expanding [27–29]. Pseudogene-expressed RNAs, a unique type of lncRNA, are considered homologous genes akin to functional genes and their defective counterparts [23, 24]. Although pseudogenes exhibit high sequence structural similarity to functional genes, they lack normal protein-coding function. Research on the expression and function of pseudogenes is in its early stages, and their specific roles in PE pathogenesis require further exploration. While previous studies have linked PDIA3P1 to promoting proliferation and invasion in hepatocellular carcinoma cells [30], its role in PE remains unexplored. In our study, we delved into the potential molecular mechanism of PDIA3P1 in trophoblasts, revealing its downregulation in PE placental tissues compared to normal tissues. Moreover, PDIA3P1 expression negatively correlated with blood pressure, gestational weeks, infant body weight, and proteinuria.

SFRP1, Wnt/*β*-catenin pathway, and trophoblast dynamics: SFRP1, a member of the secreted glycoprotein SFRP family, is implicated in various disease-related pathways [31]. Epigenetic silencing of SFRP1 can disrupt cell proliferation, migration, and invasion. The Wnt/*β*-catenin signaling pathway, a conserved axis, plays a role in diverse physiological processes, including proliferation, migration, and invasion [32]. Dysregulation of the Wnt/*β*-catenin cascade is associated with cancer and placental diseases [33, 34]. SFRP1 is recognized as a negative modulator of the Wnt signaling pathway [35]. Studies indicate that upregulation of SFRP1 expression inhibits the Wnt/*β*-catenin pathway in nonsmall cell lung cancer [36] and epithelial ovarian cancer [35]. Our study affirms that PDIA3P1 knockdown effectively upregulates SFRP1 expression, inhibiting trophoblast proliferation, invasion, and migration. Conversely, PDIA3P1 overexpression yields opposite results, establishing a link between PDIA3P1, SFRP1, and the Wnt/*β*-catenin pathway in trophoblast dynamics.

### 4.3. HuR and PDIA3P1 in Disease Regulation

HuR, an RNA-binding protein affiliated with the embryonic lethal abnormal vision family, targets mRNAs containing adenine- and uridine-rich elements in their 3′- or 5′-UTRs [15]. Cytoplasmic binding of HuR to these mRNAs typically induces adenine–uridine-rich element (ARE)-mediated mRNA decay, leading to mRNA stabilization and heightened translation [37]. In tumorigenesis, HuR interacts with specific mRNAs encoding proteins associated with cell proliferation, survival, angiogenesis, and invasion, thereby promoting cancer progression [38–40]. HuR's role in stabilizing the mRNAs of matrix metalloproteinases (MMPs) and Snails, crucial elements in epithelial–mesenchymal transition (EMT), underscores its significance in disease-related gene expression and EMT regulation [40, 41]. Our experiments verified that PDIA3P1 indirectly interacts with Snail mRNA by binding to HuR, consequently elevating Snail protein expression and contributing to EMT regulation in trophoblasts.

## 5. Conclusion

Our study positions PDIA3P1 as a pivotal regulator in the pathogenesis of PE, suggesting its potential as a diagnostic biomarker for the condition. However, certain deficiencies and limitations in our current understanding of PDIA3P1′s role in PE persist. Further investigations into the precise upstream regulatory mechanisms governing PDIA3P1 and additional animal experiments are imperative to deepen our comprehension of PDIA3P1′s involvement in PE.

## Data Availability

All data, models, and code generated or used during the study appear in the submitted article.

## Supplementary Materials

Supplementary 1 Clinical characteristics of PE patients and normal pregnancies.

Supplementary 2 Target sequences of siRNAs.

Supplementary 3 RT-PCR primers.

Supplementary 4 Analysis of the RNA transcriptome sequencing data.

Supplementary 5 Detailed process of RNA pulldown assays.

Supplementary 6 Uncut western gel imagines.

Supplementary 7 Figure S1: ethynyl deoxyuridine (EdU) immunostaining confirmed that *PDIA3P1* overexpression significantly promoted HTR-8/SVneo cells proliferation. Figure S2: EdU assay confirmed that *PDIA3P1* overexpression significantly promoted JAR cell proliferation. Figure S3: the overexpression of PDIA3P1 in HTR-8/SVneo and JAR cells resulted in a decrease in the percentage of G0 phase of the cell cycle. Figure S4: SFRP1 expression was silenced in HTR-8/SVneo and JAR cells more efficiently by si-SFRP1-1# and si-SFRP1-2# by analyzing quantitative RT-PCR data. Data are reported as mean ± SD.  ^*∗*^*p*  < 0.05.
