# Supplementary material for: Downregulated PDIA3P1 lncRNA Impairs Trophoblast Phenotype by Regulating Snail and SFRP1 in PE
Source: Anal Cell Pathol (Amst). 2024 Apr 27;2024:8972022. doi: 10.1155/2024/8972022 (PMC11074859; doi:10.1155/2024/8972022)
Supplement: Supplementary 1 — Clinical characteristics of PE patients and normal pregnancies. [file 8972022.f1.docx]

SUPPLEMENTARY TABLES

Supplementary Table 1. Clinical characteristics of PE patients and normal pregnancies.

| Variable | Normal (N=24) | PE (N=24) | P value |
| --- | --- | --- | --- |
| Maternal age (year) | 30.375±17.635 | 30.583±17.384 | p>0.05 |
| Maternal weight（Kg） | 73.637±44.294 | 74.258±52.812 | p>0.05 |
| Smoking | 0 | 0 | p>0.05 |
| Systolic blood（mmHg） | 116.5±19.391 | 160.667±28.753 | p<0.01 |
| Diastolic blood（mmHg） | 71.625±18.157 | 107.417±44.341 | p<0.01 |
| Proteinuria (g/day) | ＜0.3 | ＞0.3 | p<0.05 |
| Body weight of infant（g） | 3418.75±582.20 | 2285.417±635.82 | p<0.05 |
| Gestational age（week） | 38.583±1.732 | 33.083±2.341 | p<0.05 |
