# Supplementary material for: Downregulated PDIA3P1 lncRNA Impairs Trophoblast Phenotype by Regulating Snail and SFRP1 in PE
Source: Anal Cell Pathol (Amst). 2024 Apr 27;2024:8972022. doi: 10.1155/2024/8972022 (PMC11074859; doi:10.1155/2024/8972022)
Supplement: Supplementary 2 — Target sequences of siRNAs. [file 8972022.f2.docx]

SUPPLEMENTARY TABLES

Supplementary Table 2. Target sequences of siRNAs.

| siRNA Oligonucleotide | Sequence (5’-3’) |
| --- | --- |
| si-PDIA3P1-1# | 5'-GAUAACGGAGAUGGUAUCAUCUUAU-3' |
|  | 5‘-AUAAGAUGAUACCAUCUCCGUUAUC-3 |
| si-PDIA3P1-2# | 5'-GAGAUUCCUGUUGUUGCUAUCAGAA-3' |
|  | 5'-UUCUGAUAGCAACAACAGGAAUCUC-3 |
| si-PDIA3P1-3# | 5'-CAGCCAACAAGAAGCUAAAUCCAAA-3' |
|  | 5'-UUUGGAUUUAGCUUCUUGUUGGCUG-3 |
| si-SFRP1-1# | 5‘-CGAGAUGCUUAAGUGUGACAATT-3’ |
|  | 5'-UUGUCACACUUAAGCAUCUCGTT-3 |
| si-SFRP1-2# | 5‘-GGCCAUCAUUGAACAUCUCTT-3’ |
|  | 5'-GAGAUGUUCAAUGAUGGCCTT-3 |
| si-SFRP1-3# | 5‘-GCCACCACUUCCUCAUCAUTT-3’ |
|  | 5'-AUGAUGAGGAAGUGGUGGCTT-3 |
