# Supplementary material for: Downregulated PDIA3P1 lncRNA Impairs Trophoblast Phenotype by Regulating Snail and SFRP1 in PE
Source: Anal Cell Pathol (Amst). 2024 Apr 27;2024:8972022. doi: 10.1155/2024/8972022 (PMC11074859; doi:10.1155/2024/8972022)
Supplement: Supplementary 3 — RT-PCR primers. [file 8972022.f3.docx]

SUPPLEMENTARY TABLES

Supplementary Table 3. RT-qPCR primers.

| Gene | Sequence (5’-3’) |
| --- | --- |
| GAPDH | F：GAAGGTGAAGGTCGGAGTC |
|  | R：GAAGATGGTGATGGGATTTC |
| U1 | F：GGGAGATACCATGATCACGAAGGT |
|  | R：CCACAAATTATGCAGTCGAGTTTCCC |
| SFRP1 | F：CGTGGTTGCCCTAGAACCTA |
|  | R：GAAACTCTCTCGCTGGATGG |
| PDIA3P1 | F：GCCGAGAGGACAGAATGGAT |
|  | R：ATAACAGTGCAGCTAAGAAATGGC |
| LDHA | F：GGCCTGTGCCATCAGTATCT |
|  | R：TCTTCCAAGCCACGTAGGTC |
| PODXL | F：AAGATAAGTGCGGCATACGG |
|  | R：ATGATGCCATGCAGACGAT |
| HK1 | F：CACCCGAGGGAAGTTTAACA |
|  | R：AATACTGTGGGTGCGTCTTG |
| HK2 | F：GGCTCGCTCAACGACATTC |
|  | R：TCACCAGGATAAGCCTCACC |
| MCL1 | F：TGCATTGGCATCTTTGGAT |
|  | R：AGGGAGGGTCACTCAGGTTT |
| ITGB1 | F：AAGTTTCAAGGGCAAACGTG |
|  | R：GGACACAGGATCAGGTTGGA |
| ITGA3 | F：CTCCAGACCTCGCTTAGCAT |
|  | R：CCATTGCTGACTTCGTAGGG |
| CCND1 | F：CTTGAGGGACGCTTTGTCTG |
|  | R：TGGAAACATGCCGGTTACAT |
| CCNG1 | F：CGATAATGGCCTCAGAATGAC |
|  | R：TGCTTGGGCTGTACCTTCAT |
