# Supplementary material for: Downregulated PDIA3P1 lncRNA Impairs Trophoblast Phenotype by Regulating Snail and SFRP1 in PE
Source: Anal Cell Pathol (Amst). 2024 Apr 27;2024:8972022. doi: 10.1155/2024/8972022 (PMC11074859; doi:10.1155/2024/8972022)
Supplement: Supplementary 4 — Analysis of the RNA transcriptome sequencing data. [file 8972022.f4.pdf]

Supplementary table 4

gene_id	symbol	description	FoldChange	p-value	p-adjust	GO ID
ENSG000001	PDIA3	protein disu	-2.5157	2.67E-276	9.57E-272	GO:0003723
ENSG000001	LDHA	lactate dehy	-1.0389	4.60E-134	8.24E-130	GO:0005634
ENSG000001	CCND1	cyclin D1	-1.9694	6.22E-124	7.43E-120	GO:0001934
ENSG000001	STMN1	stathmin 1	-1.4605	3.47E-123	2.57E-119	GO:0005737
Novel.21209	-	-	1.1004	2.14E-118	1.28E-114	
ENSG000001	ITGB1	integrin sub	-1.0602	2.68E-108	1.37E-104	GO:0045766
ENSG000001	MTPN	myotrophin	-1.1209	4.27E-88	1.91E-84	GO:0005634
ENSG000000	ITGA3	integrin sub	-1.2223	1.92E-82	6.89E-79	GO:0016020
ENSG000000	RAI14	retinoic acid	-1.1322	1.25E-79	4.07E-76	GO:0005634
ENSG000001	PRNP	prion protei	-1.1713	1.44E-77	4.30E-74	GO:0043433
ENSG000002	VDAC1	voltage dep	-1.0095	3.57E-74	8.53E-71	GO:0005634
ENSG000000	CD44	CD44 molec	-1.0923	3.71E-73	8.31E-70	GO:0070374
ENSG000001	SDC1	syndecan 1	-1.3435	8.66E-70	1.65E-66	GO:0016020
ENSG000001	PODXL	podocalyxin	-1.0586	8.75E-70	1.65E-66	GO:0016477
ENSG000001	CDK2	cyclin deper	-1.1305	2.45E-69	4.18E-66	GO:0005634
ENSG000001	SRGN	serglycin	-1.071	3.37E-69	5.49E-66	GO:0005794
ENSG000001	HMGA1	high mobilit	-1.0915	1.16E-68	1.80E-65	GO:0045944
ENSG000001	PHLDA1	pleckstrin h	-1.3039	3.70E-68	5.53E-65	GO:0005634
Novel.11964	-	-	1.7887	8.18E-64	9.77E-61	
ENSG000001	S100A6	S100 calcium	-1.4305	8.97E-61	1.00E-57	GO:0005634
ENSG000001	SGPP1	sphingosine	-1.1303	2.18E-60	2.36E-57	GO:0016020
ENSG000001	COL6A2	collagen typ	-1.095	4.22E-58	4.19E-55	GO:0005615
ENSG000001	SERPINE1	serpin family	-1.3693	1.17E-55	1.10E-52	GO:0005615
ENSG000001	GJA1	gap junction	-1.5761	2.29E-54	2.00E-51	GO:0005921
ENSG000001	SHMT2	serine hydro	-1.0851	8.32E-54	7.10E-51	GO:0005634
Novel.1365	-	-	1.2051	1.21E-53	1.01E-50	
ENSG000000	SYNE2	spectrin rep	1.0795	2.71E-52	2.16E-49	GO:0005634
ENSG000000	CD99	CD99 molec	-1.0481	4.57E-52	3.56E-49	GO:0016020
ENSG000001	IL6ST	interleukin 6	-1.0555	7.73E-52	5.89E-49	GO:0008284
ENSG000001	FOSL1	FOS like 1, A	-1.4494	2.65E-51	1.98E-48	GO:0005634
ENSG000000	ACSL4	acyl-CoA sy	-1.0255	3.15E-51	2.30E-48	GO:0005737
ENSG000001	DRAP1	DR1 associa	-1.0189	9.32E-48	5.86E-45	GO:0005634
ENSG000001	TTN	titin	1.4126	2.54E-47	1.57E-44	GO:0003300
ENSG000001	CCNG1	cyclin G1	-1.0124	8.82E-47	5.27E-44	GO:0005634
ENSG000002	LYN	LYN proto-c	-1.3562	1.20E-45	6.91E-43	GO:0001934
ENSG000002	KCNQ1OT1	KCNQ1 opp	1.4356	1.18E-44	6.53E-42	
ENSG000001	ANKRD36	ankyrin repe	1.279	3.01E-44	1.61E-41	
ENSG000001	DAP	death assoc	-1.0846	4.98E-44	2.62E-41	GO:0006915
ENSG000001	SARAF	store-opera	-1.3516	1.02E-42	4.85E-40	GO:0016020
ENSG000001	EMP1	epithelial m	-1.0416	3.10E-42	1.42E-39	GO:0016020
ENSG000001	NQO1	NAD(P)H qu	-1.1789	5.35E-42	2.43E-39	GO:0003723
ENSG000001	NT5E	5'-nucleotic	-1.1861	7.30E-42	3.23E-39	GO:0005829
ENSG000001	RPL34	ribosomal p	-1.1027	3.88E-41	1.63E-38	GO:0005737
Novel.3436	-	-	1.8668	8.44E-41	3.52E-38	
ENSG000001	COL6A1	collagen typ	-1.0177	9.36E-41	3.86E-38	GO:0016020
ENSG000001	SFRP1	secreted friz	-1.384	1.00E-38	3.86E-36	GO:0005615

ENSG000002	NOL7	nucleolar pr	-1.1126	1.22E-38	4.66E-36	GO:0003723
ENSG000001	KPNA3	karyopherin	-1.013	1.22E-37	4.42E-35	GO:0005634
ENSG000001	G0S2	G0/G1 switc	-2.681	3.98E-37	1.33E-34	GO:0005739
ENSG000001	PRR26	proline rich	2.0838	1.46E-36	4.66E-34	
ENSG000001	FKBP10	FK506 bindi	-1.0709	1.73E-36	5.49E-34	GO:0016853
ENSG00000(	TOMM34	translocase	-1.2575	1.35E-35	4.03E-33	GO:0005634
ENSG000001	TGM2	transglutam	-2.0331	1.56E-35	4.61E-33	GO:0046872
ENSG00000(	MCAM	melanoma c	-1.0914	1.75E-35	5.11E-33	GO:0016020
ENSG000001	NR2F2	nuclear rece	-1.0846	5.58E-35	1.55E-32	GO:0001937
ENSG000001	TNIP1	TNFAIP3 int	-1.1672	7.44E-34	1.99E-31	GO:0005634
ENSG000001	RNF138	ring finger p	-1.224	1.08E-33	2.85E-31	GO:0005634
ENSG000001	TIMP3	TIMP metall	-1.237	2.57E-33	6.62E-31	GO:0070373
ENSG000001	WNT3	Wnt family r	-1.1567	2.83E-32	7.09E-30	GO:0005615
ENSG000002	CARMN	cardiac meso	1.642	2.87E-32	7.15E-30	
Novel.11802	-	-	-1.5735	3.01E-32	7.44E-30	
ENSG000001	LAMB3	laminin sub	-1.4699	5.00E-32	1.21E-29	GO:0007155
ENSG000002	BDNF-AS	BDNF antise	1.9104	6.86E-32	1.63E-29	
ENSG000001	PLIN2	perilipin 2	-1.3084	1.58E-31	3.57E-29	GO:0016020
ENSG000001	MARCKSL1	MARCKS like	-1.2288	1.94E-31	4.36E-29	GO:0005737
ENSG000001	PERP	PERP, TP53 a	-1.2159	2.25E-31	5.00E-29	GO:0016020
ENSG00000(	GCLM	glutamate-c	-1.245	2.53E-31	5.59E-29	GO:0005829
ENSG00000(	PLAUR	plasminoge	-1.2651	4.68E-31	1.01E-28	GO:0016020
ENSG000001	CTGF	connective t	-1.2102	7.24E-31	1.54E-28	GO:0070374
ENSG000001	PTX3	pentraxin 3	-1.7394	1.81E-30	3.78E-28	GO:0005615
ENSG000001	SPHK1	sphingosine	-1.6204	4.01E-30	8.06E-28	GO:0005737
ENSG000001	BEX3	brain expres	-1.094	5.45E-30	1.09E-27	GO:0005634
ENSG00000(	MOK	MOK protei	-1.1133	6.27E-30	1.23E-27	GO:0004672
ENSG000002	ITPRIPL2	ITPRIP like 2	-1.0052	2.97E-29	5.52E-27	GO:0016020
ENSG000002	TMEM75	transmembr	1.8919	1.08E-28	1.93E-26	
ENSG000001	ZNF682	zinc finger p	1.3126	1.28E-28	2.25E-26	GO:0003676
ENSG000001	ANGPTL4	angiopoieti	-1.7073	1.38E-28	2.42E-26	GO:0051005
ENSG00000(	CYBA	cytochrome	-1.4583	8.59E-28	1.44E-25	GO:0016020
ENSG000002	AC022149.1	integrin bet	-1.3012	1.50E-27	2.50E-25	
ENSG000001	HDAC8	histone dea	1.0231	4.67E-27	7.54E-25	GO:0031397
ENSG000002	RPS29	ribosomal p	-1.1314	4.89E-27	7.82E-25	GO:0005737
ENSG000001	FGF5	fibroblast gi	-1.2817	1.04E-26	1.59E-24	GO:0005622
ENSG000002	NUTM2B-AS	NUTM2B an	1.1211	2.11E-26	3.19E-24	
ENSG000001	EFHD2	EF-hand do	-1.0824	2.36E-26	3.54E-24	GO:0016020
ENSG00000(	FSCN1	fascin actin-	-1.0722	2.86E-26	4.24E-24	GO:0003723
ENSG000001	PHF19	PHD finger p	-1.0537	4.07E-25	5.71E-23	GO:0046872
Novel.27418	-	-	1.5534	2.12E-24	2.81E-22	
ENSG000002	LINC-PINT	long interge	1.714	7.40E-24	9.63E-22	
ENSG00000(	PPP1R12B	protein pho	1.0258	1.05E-23	1.35E-21	GO:0005737
ENSG000001	H1FX	H1 histone f	-1.1623	1.12E-23	1.43E-21	GO:0003723
Novel.27411	-	-	1.6323	1.59E-23	2.01E-21	
ENSG000001	RBM38	RNA bindin	-1.2051	2.48E-23	3.12E-21	GO:0003676
ENSG000001	ITGA2	integrin sub	-1.0216	3.34E-23	4.17E-21	GO:0016020
ENSG000001	PTP4A3	protein tyro	-1.3458	6.36E-23	7.77E-21	GO:0016020

ENSG000001	PACSIN3	protein kina	-1.1306	6.46E-23	7.87E-21	GO:0005737
ENSG00000(	IL11	interleukin 1	-1.2188	8.47E-23	1.02E-20	GO:0008083
ENSG000001	SH3BGRL3	SH3 domain	-1.0451	8.90E-23	1.07E-20	GO:0005634
ENSG000001	AEBP1	AE binding	-1.1388	8.97E-23	1.07E-20	GO:0005615
ENSG000002	MIR29B2CH(	MIR29B2 an	1.7594	3.00E-22	3.44E-20	
ENSG000001	RAB31	RAB31, mem	-1.2085	5.28E-22	5.89E-20	GO:0016020
ENSG000001	SPOCD1	SPOC doma	-1.6718	2.40E-21	2.53E-19	GO:0006351
ENSG000001	ADAM32	ADAM meta	1.8289	3.33E-21	3.50E-19	GO:0016020
ENSG000002	AC092828.1	novel transc	2.4124	5.92E-21	6.08E-19	
ENSG000001	HIST1H1D	histone clus	2.6148	4.32E-20	4.00E-18	GO:0003723
ENSG000001	UBL4A	ubiquitin lik	-1.0325	8.04E-20	7.16E-18	GO:0005634
ENSG00000(	DKK3	dickkopf WI	-1.0461	1.79E-19	1.54E-17	GO:0090090
ENSG000001	GAMT	guanidinoa(	-1.6344	2.01E-19	1.72E-17	GO:0008168
ENSG000001	SLCO4A1	solute carrie	-1.069	2.71E-19	2.29E-17	GO:0016020
ENSG000002	STARD13-A:	STARD13 an	2.1014	4.96E-19	4.12E-17	
ENSG000001	RP2	RP2, ARL3 G	-1.2703	5.41E-19	4.45E-17	GO:0005737
ENSG000001	EFNB3	ephrin B3	-1.3048	5.94E-19	4.85E-17	GO:0016020
ENSG000001	PDIA3P1	protein disu	-1.7951	1.11E-18	8.78E-17	
ENSG000002	RALGAPA1F	Ral GTPase a	1.6542	1.38E-18	1.08E-16	
ENSG000002	AL031281.2	novel protei	1.673	1.54E-18	1.20E-16	GO:0000166
ENSG00000(	MRPL43	mitochondri	-1.1603	1.92E-18	1.49E-16	GO:0003723
Novel.23497	-	-	1.4006	1.98E-18	1.53E-16	
ENSG000001	PCLO	piccolo pres	1.6968	2.69E-18	2.05E-16	GO:0046872
ENSG000002	HELLPAR	HELLP assoc	1.0314	1.36E-17	9.60E-16	
ENSG000002	SRMP2	spermidine :	2.7232	1.60E-17	1.12E-15	
ENSG000001	ZCCHC24	zinc finger C	-1.2286	1.81E-17	1.26E-15	GO:0003676
ENSG000001	OMD	osteomodul	2.9479	2.69E-17	1.83E-15	GO:0007155
ENSG000001	PTGFR	prostagland	-1.238	4.05E-17	2.71E-15	GO:0005737
ENSG000001	PRXL2B	peroxiredox	-1.3865	7.46E-17	4.81E-15	GO:0005737
ENSG000002	ZNRD1ASP	zinc ribbon	1.0229	1.08E-16	6.87E-15	
ENSG000002	AC058791.1	novel transc	1.9644	1.10E-16	7.00E-15	
ENSG000002	AC087294.1	novel transc	1.3041	1.24E-16	7.82E-15	
ENSG000002	AC018628.1	TEC	1.3468	1.94E-16	1.21E-14	
ENSG000001	DUSP7	dual specific	-1.0673	2.02E-16	1.25E-14	GO:0005737
ENSG000001	ANPEP	alanyl amin(	-1.19	3.21E-16	1.93E-14	GO:0016020
ENSG000001	ZNF66	zinc finger p	1.2902	4.28E-16	2.53E-14	GO:0003676
ENSG000001	HRNR	hornerin	1.7947	4.72E-16	2.77E-14	GO:0005634
ENSG000002	AC138409.1	integral mer	1.3164	5.15E-16	3.01E-14	
ENSG000002	AF117829.1	uncharacteri	1.1367	6.96E-16	4.00E-14	
ENSG000001	OGN	osteoglycin	1.8124	8.85E-16	5.02E-14	GO:0005615
ENSG000002	PGM5P2	phosphoglu	1.134	9.29E-16	5.25E-14	
ENSG000001	NRN1	neuritin 1	-2.1967	9.84E-16	5.55E-14	GO:0016020
ENSG00000(	NRP1	neuropilin 1	-1.2181	1.06E-15	5.96E-14	GO:0016020
ENSG000001	C9orf84	chromosom	1.3771	1.39E-15	7.70E-14	GO:0016787
ENSG000002	Z82217.1	novel transc	2.0837	2.70E-15	1.47E-13	
ENSG000001	WNK4	WNK lysine	-1.2947	2.72E-15	1.48E-13	GO:0000166
ENSG000001	RPL22L1	ribosomal p	-1.1527	2.92E-15	1.58E-13	GO:0005622
ENSG000001	ASPN	asporin	2.2403	3.06E-15	1.65E-13	GO:0005509

ENSG000001	LRRTM2	leucine rich	2.092	4.01E-15	2.12E-13	GO:0016020
ENSG000002	AC067930.6	TEC	2.3139	4.34E-15	2.28E-13	
ENSG000001	HIST1H1C	histone clus	1.3051	5.27E-15	2.75E-13	GO:0003723
ENSG000001	SOCS2	suppressor c	-1.4198	5.40E-15	2.81E-13	GO:0005737
ENSG000001	ZNF782	zinc finger p	1.2229	5.95E-15	3.09E-13	GO:0003676
ENSG000001	MT2A	metallothior	-1.1114	6.52E-15	3.38E-13	GO:0005634
ENSG000001	EGFL7	EGF like dor	-1.1402	7.06E-15	3.66E-13	GO:0005509
ENSG000001	IER3	immediate e	-1.1275	8.95E-15	4.57E-13	GO:0043066
ENSG000002	HIST2H2BF	histone clus	2.2453	9.16E-15	4.66E-13	GO:0005634
Novel.15664	-	-	1.0985	9.83E-15	4.96E-13	
ENSG000002	AC016831.4	novel transc	2.3065	1.05E-14	5.29E-13	
ENSG000001	GPRC5B	G protein-cc	-1.1751	1.07E-14	5.36E-13	GO:0016020
ENSG000001	ITGA7	integrin sub	-1.0738	1.39E-14	6.91E-13	GO:0016020
ENSG000001	HBEGF	heparin binc	-1.2429	1.45E-14	7.21E-13	GO:0005615
ENSG000002	LUCAT1	lung cancer	-1.3728	1.47E-14	7.30E-13	
ENSG000001	LY6K	lymphocyte	-1.4778	1.62E-14	8.01E-13	GO:0005737
ENSG000001	LRRC37A3	leucine rich	1.3894	1.67E-14	8.22E-13	GO:0016020
ENSG000001	HPCAL1	hippocalcin	-1.028	1.86E-14	9.15E-13	GO:0016020
ENSG000001	IRF2BPL	interferon re	-1.0107	3.08E-14	1.48E-12	GO:0005634
ENSG000001	ZBTB20	zinc finger a	1.0239	3.46E-14	1.64E-12	GO:0003676
ENSG000001	DNPH1	2'-deoxynuc	-1.0697	5.43E-14	2.51E-12	GO:0005634
ENSG000001	HTR2B	5-hydroxytr	2.9217	5.67E-14	2.60E-12	GO:0005737
ENSG000001	CTXN1	cortexin 1	-1.7155	6.06E-14	2.76E-12	GO:0016020
ENSG000002	SLFNL1-AS1	SLFNL1 anti	1.0173	6.14E-14	2.79E-12	
ENSG000001	SLC16A6	solute carrie	1.4769	7.17E-14	3.22E-12	GO:0016020
ENSG000002	DNAJC19	DnaJ heat sh	-1.0936	7.39E-14	3.31E-12	GO:0016020
ENSG000001	ECM2	extracellular	1.4743	7.43E-14	3.33E-12	GO:0005576
ENSG000001	COMMD8	COMM dom	-1.034	7.44E-14	3.33E-12	GO:0005634
Novel.17486	-	-	2.1253	8.09E-14	3.60E-12	
ENSG000002	AC026124.1	novel transc	1.8741	8.48E-14	3.77E-12	
ENSG000001	TRIB3	tribbles pseu	-1.0754	9.93E-14	4.35E-12	GO:0003714
ENSG000002	ADGRG1	adhesion G	-1.188	1.04E-13	4.54E-12	GO:0005622
ENSG000002	AC068587.4	uncharacteri	1.1236	1.14E-13	4.94E-12	
ENSG000001	CKMT2	creatine kina	1.7579	1.64E-13	6.99E-12	GO:0016020
ENSG000002	ERVFRD-1	endogenous	2.3677	1.69E-13	7.17E-12	GO:0016020
ENSG000001	FRMD5	FERM doma	1.152	1.78E-13	7.54E-12	GO:0016020
ENSG000001	EPHA4	EPH recepto	1.0713	1.88E-13	7.95E-12	GO:0016020
ENSG000001	PM20D1	peptidase M	1.4152	2.31E-13	9.69E-12	GO:0046872
ENSG000002	FLJ42393	uncharacteri	1.9298	3.27E-13	1.35E-11	
ENSG000002	LINC-PINT	long interge	1.3381	3.46E-13	1.42E-11	
ENSG000002	AC007878.1	novel transc	1.8444	3.87E-13	1.58E-11	
ENSG000000	MXD1	MAX dimeriz	-1.0012	5.45E-13	2.21E-11	GO:0005829
ENSG000001	LRRC75A	leucine rich	1.1176	5.62E-13	2.27E-11	GO:0005737
ENSG000001	HIST2H2BE	histone clus	1.4179	6.15E-13	2.47E-11	GO:0005634
Novel.15669	-	-	2.024	6.40E-13	2.56E-11	
Novel.27420	-	-	1.3865	6.98E-13	2.79E-11	
ENSG000002	RAI1-AS1	RAI1 antiser	2.3457	8.17E-13	3.23E-11	
ENSG000002	TMEM14EP	transmembr	2.6383	9.73E-13	3.81E-11	

ENSG000001	PIGM	phosphatidy	-1.113	1.03E-12	4.03E-11	GO:0016020
ENSG000001	IGF2BP2-AS	IGF2BP2 ant	1.5239	1.08E-12	4.21E-11	
ENSG000002	AC068491.4	TEC	2.0269	1.26E-12	4.89E-11	
ENSG000002	SMCR5	Smith-Mage	2.1898	1.37E-12	5.26E-11	
ENSG000001	LDHB	lactate dehy	-1.0822	1.46E-12	5.59E-11	GO:0005737
ENSG000002	TMEM158	transmembr	-2.701	1.62E-12	6.16E-11	GO:0016020
ENSG000002	AC003681.1	novel transc	1.2182	1.64E-12	6.22E-11	
ENSG00000(	MGLL	monoglycer	-1.4141	2.33E-12	8.62E-11	GO:0005737
Novel.19478	-	-	2.058	3.00E-12	1.10E-10	
ENSG00000(	FOXC1	forkhead bc	-1.1957	3.20E-12	1.16E-10	GO:0045944
ENSG000001	ANKRD36B	ankyrin repe	1.1066	3.23E-12	1.17E-10	GO:0005515
ENSG000001	ZNF410	zinc finger p	1.134	4.33E-12	1.54E-10	GO:0003676
ENSG000001	CXCL12	C-X-C motif	-1.1381	4.45E-12	1.58E-10	GO:0008083
ENSG00000(	SEMA3B	semaphorin	-1.0629	6.25E-12	2.19E-10	GO:0005783
ENSG000001	LIMD2	LIM domain	-1.3702	6.70E-12	2.34E-10	GO:0005634
ENSG000002	LINC00513	long interge	1.7539	7.85E-12	2.72E-10	
ENSG000002	AC015799.1	novel transc	1.2655	1.17E-11	3.98E-10	
ENSG000001	DUSP5	dual specific	-1.3753	1.20E-11	4.05E-10	GO:0005634
ENSG000001	HIST1H1E	histone clus	2.5213	1.24E-11	4.19E-10	GO:0003723
ENSG000002	LCMT1-AS2	LCMT1 antis	2.1697	1.25E-11	4.20E-10	
ENSG000002	AC103810.2	novel transc	1.8782	1.27E-11	4.27E-10	
ENSG000001	MPZL3	myelin prote	1.4427	1.40E-11	4.69E-10	GO:0016020
ENSG000002	MT-RNR1	mitochondri	2.2319	1.54E-11	5.13E-10	
ENSG000001	TRIM47	tripartite mc	-1.0096	1.61E-11	5.37E-10	GO:0005634
ENSG00000(	CEACAM1	carcinoembr	2.0497	1.70E-11	5.61E-10	GO:0016020
ENSG000002	AC009951.1	TEC	2.4167	1.71E-11	5.65E-10	
ENSG000002	HIST1H4E	histone clus	2.0316	2.44E-11	7.98E-10	GO:0003723
Novel.1701(	-	-	1.4169	2.56E-11	8.36E-10	
ENSG000002	AC002558.3	novel transc	1.056	3.20E-11	1.04E-09	
ENSG000001	ABI3	ABI family m	-1.6063	3.99E-11	1.28E-09	GO:0005737
ENSG000002	LINC01572	long interge	1.1428	4.04E-11	1.29E-09	
ENSG000002	AC067931.2	TEC	1.955	4.57E-11	1.45E-09	
ENSG000001	SLFNL1	schlafen like	1.0312	4.96E-11	1.57E-09	GO:0000166
ENSG000002	GNAS-AS1	GNAS antise	1.4315	5.49E-11	1.73E-09	
ENSG000002	MT-RNR2	mitochondri	1.6405	7.04E-11	2.19E-09	
ENSG000001	OMG	oligodendrc	1.3985	7.29E-11	2.27E-09	GO:0016020
ENSG000002	AATBC	apoptosis as	1.3964	7.46E-11	2.31E-09	
ENSG00000(	GADD45B	growth arres	-1.1041	8.42E-11	2.59E-09	GO:0005634
ENSG000001	HIST1H2BD	histone clus	1.0984	1.06E-10	3.23E-09	GO:0005634
ENSG000001	PLTP	phospholipi	-1.0071	1.19E-10	3.61E-09	GO:0005615
ENSG000001	ENKD1	enkurin don	-1.0573	1.23E-10	3.72E-09	GO:0005515
ENSG000002	AC087241.4	novel transc	2.8107	1.29E-10	3.90E-09	
ENSG000001	MAFF	MAF bZIP tr	-1.0258	1.51E-10	4.48E-09	GO:0005634
ENSG000002	POU5F2	POU domain	1.9352	1.54E-10	4.56E-09	GO:0005634
ENSG000001	HIST1H1B	histone clus	1.8937	1.90E-10	5.53E-09	GO:0003723
ENSG000002	AC138409.2	Putative bet	1.1028	1.91E-10	5.56E-09	
ENSG000001	METTL26	methyltrans	-1.0659	2.03E-10	5.89E-09	
ENSG000002	TMEM161B-	TMEM161B a	1.2488	2.46E-10	7.07E-09	

ENSG000001	PLAU	plasminoge	-1.2868	2.60E-10	7.43E-09	GO:0008236
ENSG000001	ZNF138	zinc finger p	1.0746	3.31E-10	9.34E-09	GO:0003676
ENSG000001	ETV4	ETS variant 4	-1.5873	3.47E-10	9.74E-09	GO:0005634
ENSG000001	ANKRD20A5	ankyrin repe	1.3849	3.92E-10	1.09E-08	
ENSG000001	KRT34	keratin 34	-2.2574	4.23E-10	1.17E-08	GO:0005198
ENSG000002	HSPA1A	heat shock p	-1.1553	4.57E-10	1.26E-08	GO:0010628
ENSG000001	TPPP	tubulin poly	1.0374	4.76E-10	1.31E-08	GO:0005634
ENSG000002	ALMS1-IT1	ALMS1 intro	1.4557	5.80E-10	1.57E-08	
ENSG000002	OFD1P17	OFD1 pseuc	1.9727	5.80E-10	1.57E-08	
ENSG000002	KRT8P14	keratin 8 pse	-1.0859	5.91E-10	1.60E-08	
ENSG000001	ZNF235	zinc finger p	1.3505	6.64E-10	1.79E-08	GO:0003676
ENSG000002	AL022323.4	novel transc	1.5378	8.08E-10	2.16E-08	
ENSG000000	FLT4	fms related	-1.065	8.43E-10	2.24E-08	GO:0005634
ENSG000000	ATP2A3	ATPase sarc	-1.36	9.36E-10	2.48E-08	GO:0005634
ENSG000001	ZNF695	zinc finger p	1.0403	9.67E-10	2.56E-08	GO:0003676
ENSG000002	AP002833.3	TEC	1.5459	1.02E-09	2.69E-08	
ENSG000002	AC092881.1	TEC	1.8951	1.12E-09	2.94E-08	
ENSG000002	AL353795.3	TEC	2.289	1.28E-09	3.32E-08	
ENSG000002	MALAT1	metastasis a	1.1161	1.42E-09	3.66E-08	
ENSG000002	AL358216.1	novel transc	2.1897	1.52E-09	3.91E-08	
ENSG000002	GAPDHP1	glyceraldehy	-1.1254	1.80E-09	4.59E-08	
ENSG000002	AC103796.1	novel transc	2.1735	1.81E-09	4.60E-08	
ENSG000001	STXBP5L	syntaxin bin	2.1331	1.82E-09	4.62E-08	GO:0005737
ENSG000001	CLEC11A	C-type lectin	-1.0148	1.82E-09	4.62E-08	GO:0005737
ENSG000002	HSPA1B	heat shock p	-1.0438	2.12E-09	5.31E-08	GO:0010628
ENSG000001	UBE3D	ubiquitin pr	1.0247	2.34E-09	5.82E-08	GO:0005737
ENSG000002	HIST1H2AE	histone clus	2.0081	2.85E-09	6.99E-08	GO:0005634
ENSG000001	MYEOV	myeloma ov	-2.2276	2.89E-09	7.08E-08	
ENSG000002	EDDM13	epididymal p	1.55	3.19E-09	7.79E-08	GO:0016020
ENSG000001	DUSP4	dual specific	-1.5055	3.40E-09	8.25E-08	GO:0005634
ENSG000002	AC008105.3	novel transc	-1.2659	4.53E-09	1.08E-07	
ENSG000002	AP003900.1	novel transc	2.1745	4.80E-09	1.14E-07	
ENSG000001	ESM1	endothelial	-3.8382	4.91E-09	1.16E-07	GO:0005576
ENSG000002	MIR1206	microRNA 1	2.2484	5.00E-09	1.18E-07	
ENSG000002	AC017002.1	novel transc	2.3608	5.74E-09	1.35E-07	
ENSG000001	CYP3A5	cytochrome	1.5119	5.84E-09	1.37E-07	GO:0016020
ENSG000001	SOX9	SRY-box 9	-1.8115	5.97E-09	1.40E-07	GO:0045944
ENSG000001	RASGRP1	RAS guanyl	1.3978	6.31E-09	1.47E-07	GO:0005737
ENSG000001	INHBB	inhibin subu	-1.4991	7.07E-09	1.64E-07	GO:0008083
ENSG000002	AL136164.3	TEC	1.1806	7.33E-09	1.69E-07	
ENSG000001	PSG6	pregnancy s	3.0842	7.73E-09	1.77E-07	GO:0005576
ENSG000002	SNHG14	small nucleo	2.1782	7.85E-09	1.80E-07	
ENSG000002	AC092801.1	novel transc	2.4556	8.32E-09	1.90E-07	
ENSG000002	LINC01535	long interge	1.2427	9.39E-09	2.13E-07	
ENSG000001	IGFBP4	insulin like g	-1.1907	9.96E-09	2.25E-07	GO:0005576
ENSG000001	ARID5A	AT-rich inte	-1.1042	1.00E-08	2.26E-07	GO:0003723
ENSG000002	RF00598	-	2.7177	1.06E-08	2.39E-07	
ENSG000002	PCDHGB6	protocadher	2.116	1.14E-08	2.55E-07	GO:0016020

ENSG000001	ADM2	adrenomedu	-1.3838	1.19E-08	2.67E-07	GO:0005622
ENSG000002	AF131215.2	novel transc	1.7733	1.21E-08	2.69E-07	
ENSG000001	ANGPTL1	angiopoietir	1.8743	1.32E-08	2.92E-07	GO:0005576
ENSG000002	N4BP2L2-IT	N4BPL2 intr	1.3074	1.32E-08	2.92E-07	
Novel.1364	-	-	1.9774	1.39E-08	3.07E-07	
ENSG000001	JUP	junction pla	-1.3066	1.62E-08	3.53E-07	GO:0043537
ENSG000001	GNGT2	G protein su	-1.3895	1.63E-08	3.54E-07	GO:0016020
ENSG000001	NDUFV2	NADH:ubiqu	1.663	1.74E-08	3.77E-07	GO:0016020
ENSG000002	EPN2-AS1	EPN2 antise	1.1927	2.01E-08	4.31E-07	
ENSG000002	GMDS-DT	GMDS diver	1.4263	2.07E-08	4.43E-07	
ENSG000002	HIST1H3A	histone clus	3.3617	2.09E-08	4.46E-07	GO:0005634
ENSG000002	AC091132.4	novel transc	1.7516	2.09E-08	4.46E-07	
ENSG000001	GCNT7	glucosaminy	1.5747	2.36E-08	5.01E-07	GO:0016020
ENSG000001	ZNF233	zinc finger p	1.7213	2.44E-08	5.17E-07	GO:0003676
ENSG000001	HIST1H2AI	histone clus	2.2932	2.46E-08	5.19E-07	GO:0005634
ENSG000002	FOXP1-IT1	FOXP1 intro	2.2999	2.59E-08	5.44E-07	
ENSG000001	HIST1H2BC	histone clus	1.957	2.76E-08	5.76E-07	GO:0005634
ENSG000002	HIST1H2BG	histone clus	1.7114	2.81E-08	5.86E-07	GO:0005634
ENSG000002	AC008781.2	uncharacteri	2.0714	2.93E-08	6.07E-07	
ENSG000001	PDZD2	PDZ domain	1.1208	3.04E-08	6.28E-07	GO:0005634
ENSG000001	INSYN2	inhibitory sy	1.658	3.05E-08	6.29E-07	GO:0016020
Novel.17002	-	-	1.8364	3.47E-08	7.11E-07	
ENSG000001	VWA1	von Willebra	-1.3245	3.64E-08	7.42E-07	GO:0005576
ENSG000002	AC004884.2	novel transc	2.708	3.90E-08	7.91E-07	
ENSG000002	AP002833.2	novel transc	2.7005	4.08E-08	8.26E-07	
ENSG000002	Z97832.2	novel transc	1.255	4.15E-08	8.38E-07	
ENSG000002	AL132655.1	novel transc	1.5853	4.33E-08	8.75E-07	
ENSG000001	MAGI2	membrane a	1.0708	4.71E-08	9.46E-07	GO:0005634
ENSG000002	AC091181.1	TEC	2.0008	4.78E-08	9.57E-07	
ENSG000002	AC024451.2	NSFL1 (p97)	1.8892	4.78E-08	9.58E-07	
ENSG000002	AC131649.2	TEC	1.7327	5.08E-08	1.02E-06	
ENSG000001	GPR18	G protein-co	2.1622	5.13E-08	1.02E-06	GO:0016020
ENSG000001	ZNF750	zinc finger p	2.031	5.25E-08	1.05E-06	GO:0005634
ENSG000002	AL049830.4	intraflagella	2.5541	5.68E-08	1.13E-06	
ENSG000002	LINC02407	long interge	-1.6142	5.70E-08	1.13E-06	
ENSG000002	AC091132.3	TEC	1.3425	5.82E-08	1.15E-06	
ENSG000001	PCOLCE	procollagen	-1.079	5.91E-08	1.17E-06	GO:0005615
ENSG000002	AC002558.2	novel transc	1.5167	6.20E-08	1.22E-06	
ENSG000001	NT5C	5', 3'-nucleo	-1.0243	6.91E-08	1.35E-06	GO:0016311
Novel.5903	-	-	1.8242	6.95E-08	1.36E-06	
ENSG000002	AC004898.1	UBX domain	2.3919	7.14E-08	1.39E-06	
ENSG000002	PSG1	pregnancy s	1.8924	7.94E-08	1.54E-06	GO:0005515
ENSG000001	UBTD1	ubiquitin do	-1.065	8.25E-08	1.59E-06	GO:0005515
ENSG000002	RN7SL648P	RNA, 7SL, cy	2.5607	9.02E-08	1.74E-06	
ENSG000002	AC026362.2	novel transc	1.4752	9.30E-08	1.79E-06	
ENSG000001	LPAR6	lysophospha	1.6637	9.43E-08	1.81E-06	GO:0016020
ENSG000001	DNAH7	dynein axon	1.1198	9.77E-08	1.87E-06	GO:0005737
ENSG000001	CCDC85B	coiled-coil c	-1.0051	1.05E-07	2.00E-06	GO:0005634

ENSG000001	TUBB2B	tubulin beta	-1.2196	1.07E-07	2.03E-06	GO:0005634
Novel.1265(	-	-	1.0377	1.15E-07	2.17E-06	
ENSG000002	AC115223.1	ribosomal p	-1.1499	1.16E-07	2.18E-06	
ENSG000002	AC138951.1	POM121 me	1.4977	1.21E-07	2.26E-06	
ENSG000001	LEF1	lymphoid er	-1.7894	1.24E-07	2.32E-06	GO:0045944
ENSG000002	AL359918.1	proteasome	1.6661	1.30E-07	2.43E-06	
ENSG000002	AC122718.2	DEAD (Asp-	2.2976	1.32E-07	2.45E-06	
ENSG000002	AC127070.4	novel transc	1.3648	1.32E-07	2.45E-06	
ENSG000002	PCDHGA8	protocadher	1.6886	1.36E-07	2.51E-06	GO:0016020
ENSG000002	HNRNPA1P(	heterogenec	2.8755	1.38E-07	2.54E-06	
ENSG000001	CACNB2	calcium volt	1.2099	1.39E-07	2.57E-06	GO:1904879
ENSG000002	AKT3-IT1	AKT3 intron	2.0209	1.44E-07	2.64E-06	
ENSG000002	AL589655.1	pseudogene	2.7749	1.56E-07	2.85E-06	
ENSG000002	KCNQ5-IT1	KCNQ5 intro	1.8351	1.62E-07	2.96E-06	
ENSG000002	MIR503HG	MIR503 host	-1.0901	1.67E-07	3.03E-06	
ENSG000002	PRKCA-AS1	PRKCA antis	2.0062	1.68E-07	3.04E-06	
ENSG000002	RAB30-AS1	RAB30 antis	1.0215	1.73E-07	3.14E-06	
ENSG000002	AC005476.1	PDZ domair	2.6116	1.78E-07	3.22E-06	
ENSG000001	CXCL1	C-X-C motif	-2.4698	1.96E-07	3.54E-06	GO:0008083
ENSG00000(	KCNK2	potassium t	-1.2391	1.99E-07	3.58E-06	GO:0016020
ENSG000001	B3GALT2	beta-1,3-ga	1.7731	2.00E-07	3.59E-06	GO:0016020
ENSG000002	AC005261.5	TEC	1.1325	2.04E-07	3.66E-06	
ENSG00000(	CACNA1G	calcium volt	-1.7062	2.06E-07	3.68E-06	GO:0005737

hannel activ	hsa:8913	hsa04010;hs	MAPK signaling pathway		21.4	71.21
ENSG000002	RNU4-25P	RNA, U4 sm	2.3376	2.11E-07	3.77E-06	
ENSG00000(	CLDN18	claudin 18	1.6018	2.12E-07	3.79E-06	GO:0016020
ENSG000001	KLF16	Kruppel like	-1.0209	2.35E-07	4.16E-06	GO:0003676
ENSG000002	RNA5-8SN2	RNA, 5.8S ri	1.9029	2.46E-07	4.33E-06	
ENSG000002	AF127577.6	TEC	1.7407	2.50E-07	4.41E-06	
ENSG000002	AC005480.1	novel transc	1.2932	2.56E-07	4.50E-06	
ENSG000002	AC108471.1	ribosomal p	1.9996	2.67E-07	4.66E-06	
ENSG000001	DNAH6	dynein axor	1.3017	2.87E-07	4.99E-06	GO:0005737
ENSG000002	AC105233.5	novel transc	1.1749	2.89E-07	5.03E-06	
ENSG000002	AL157938.3	novel transc	2.0735	2.90E-07	5.04E-06	
ENSG000001	TNFRSF11A	TNF recepto	-1.1345	2.90E-07	5.04E-06	GO:0051091
ENSG000002	AF131215.1	ribosomal p	2.5832	3.09E-07	5.36E-06	
ENSG00000(	KCNK6	potassium t	-1.2788	3.12E-07	5.40E-06	GO:0016020
ENSG000002	AL049840.5	novel transc	1.4002	3.32E-07	5.72E-06	
ENSG000002	AC127035.1	novel transc	1.8505	3.58E-07	6.14E-06	
ENSG000001	ANKAR	ankyrin and	1.0562	3.65E-07	6.26E-06	GO:0016020
ENSG000001	ZNF670-ZN	ZNF670-ZN	1.8753	3.66E-07	6.26E-06	GO:0003676
ENSG000002	AL138759.1	novel transc	1.4766	3.69E-07	6.30E-06	
ENSG000002	HIST1H3C	histone clus	2.733	3.71E-07	6.32E-06	GO:0005634
ENSG000002	HIST1H2AL	histone clus	2.111	3.79E-07	6.44E-06	GO:0005634
ENSG000002	AC139887.2	uncharacteri	1.1222	4.06E-07	6.88E-06	
ENSG000002	ZNF571-AS:	ZNF571 anti	1.5616	4.53E-07	7.62E-06	

ENSG000002	AC010422.4	novel transc	2.0747	4.63E-07	7.76E-06	
ENSG000002	AP001442.1	novel transc	1.6104	4.63E-07	7.77E-06	
ENSG000002	AC018628.2	novel transc	1.7611	4.90E-07	8.17E-06	
ENSG000002	ZNF853	zinc finger p	-1.3702	5.08E-07	8.44E-06	GO:0003676
ENSG000002	Z95331.1	novel transc	1.2064	5.20E-07	8.63E-06	
ENSG000001	CCDC171	coiled-coil c	1.0044	5.27E-07	8.73E-06	
ENSG000001	PDE10A	phosphodie	1.0325	5.61E-07	9.26E-06	GO:0005737
ENSG000001	KCNJ13	potassium v	1.8933	5.61E-07	9.26E-06	GO:0016020
ENSG000001	DUSP6	dual specific	-1.383	5.91E-07	9.70E-06	GO:0005737
ENSG000002	AL157938.2	novel transc	2.1389	5.96E-07	9.76E-06	
ENSG000002	ZFPM2-AS1	ZFPM2 antis	1.2781	5.98E-07	9.78E-06	
ENSG000000	CDC42	cell division	1.446	6.88E-07	1.11E-05	GO:0005737
Novel.20156	-	-	1.5096	6.93E-07	1.12E-05	
ENSG000002	RNA5SP278	RNA, 5S ribo	3.0963	6.98E-07	1.13E-05	
ENSG000001	NEB	nebulin	1.1942	7.02E-07	1.13E-05	GO:0005737
ENSG000002	GPSM3	G protein si	-1.0244	7.04E-07	1.14E-05	GO:0030695
ENSG000002	AC016596.1	ribosomal p	-2.5347	7.11E-07	1.15E-05	
ENSG000002	TAS2R30	taste 2 recep	2.9562	7.16E-07	1.15E-05	GO:0016020
ENSG000001	OR2L5	olfactory rec	3.3159	7.22E-07	1.16E-05	GO:0016020
ENSG000000	RAB27B	RAB27B, me	-1.2653	7.82E-07	1.25E-05	GO:0003924
ENSG000002	DDX50P1	DEAD-box h	1.5864	8.15E-07	1.30E-05	
ENSG000001	IMPA2	inositol mor	-1.1025	8.20E-07	1.31E-05	GO:0046854
ENSG000002	HIST1H2BO	histone clus	2.0945	8.23E-07	1.31E-05	GO:0005634
ENSG000002	AC005183.1	SDA1 doma	1.939	8.26E-07	1.32E-05	
ENSG000002	AF131215.4	novel transc	1.4622	8.46E-07	1.35E-05	
ENSG000002	AC009032.1	novel transc	1.4017	9.24E-07	1.46E-05	
ENSG000002	PSG10P	pregnancy s	2.5449	9.30E-07	1.47E-05	
ENSG000002	AL359922.2	novel transc	1.1536	9.79E-07	1.54E-05	
ENSG000002	PCDHGA11	protocadher	2.1531	9.93E-07	1.56E-05	GO:0016020
ENSG000001	GPR22	G protein-c	2.2529	1.01E-06	1.58E-05	GO:0016020
ENSG000002	HIST1H2BH	histone clus	1.3118	1.01E-06	1.58E-05	GO:0005634
ENSG000001	NRGN	neurogranir	-1.8099	1.05E-06	1.64E-05	GO:0016301
ENSG000002	OR2L1P	olfactory rec	3.2624	1.13E-06	1.74E-05	
ENSG000001	PGF	placental gr	-1.3245	1.13E-06	1.75E-05	GO:0016020
ENSG000002	AL133230.1	novel transc	1.3805	1.14E-06	1.76E-05	
ENSG000002	LINC00894	long interge	1.3435	1.14E-06	1.76E-05	
ENSG000002	HIST1H2BM	histone clus	2.4283	1.14E-06	1.77E-05	GO:0005634
ENSG000002	AC011447.6	TEC	1.0605	1.16E-06	1.78E-05	
ENSG000001	FLG	filaggrin	1.1506	1.17E-06	1.80E-05	GO:0005634
ENSG000002	AC078846.1	novel transc	1.1546	1.18E-06	1.82E-05	
ENSG000001	DCLK1	doublecortir	-1.0751	1.34E-06	2.03E-05	GO:0005622
ENSG000001	DPM3	dolichyl-ph	-1.219	1.35E-06	2.05E-05	GO:0016020
ENSG000001	SHC2	SHC adapto	-1.1681	1.44E-06	2.17E-05	GO:0035556
ENSG000002	HIST1H2BF	histone clus	2.1422	1.44E-06	2.17E-05	GO:0005634
ENSG000002	PSMD10P1	proteasome	1.2737	1.46E-06	2.20E-05	
ENSG000001	FOXC2	forkhead bo	-1.2553	1.46E-06	2.20E-05	GO:0010595
ENSG000002	AC011933.2	novel transc	1.9442	1.47E-06	2.21E-05	
ENSG000000	SERPIND1	serpin family	1.8782	1.51E-06	2.26E-05	GO:0005615

ENSG00000(	CYB561	cytochrome	-1.1971	1.60E-06	2.39E-05	GO:0016020
ENSG00000	YPEL4	yippee like 4	1.3689	1.68E-06	2.50E-05	GO:0005634
ENSG00000	AC009044.1	TEC	3.2642	1.72E-06	2.56E-05	
ENSG00000	SPDYE6	speedy/RIN	1.0756	1.80E-06	2.67E-05	GO:0019901
ENSG00000	LINC02577	long interge	1.2948	1.89E-06	2.78E-05	
ENSG00000	AC008498.2	novel transc	2.2975	1.95E-06	2.86E-05	
ENSG00000	ST7-OT4	ST7 overlap	2.2088	1.98E-06	2.91E-05	
ENSG00000	PSG7	pregnancy s	2.4347	1.99E-06	2.92E-05	GO:0005576
ENSG00000	TAS2R31	taste 2 recep	2.2003	2.00E-06	2.94E-05	GO:0016020
ENSG00000	AC005730.3	novel transc	1.4352	2.24E-06	3.27E-05	
ENSG00000	EIF4BP3	eukaryotic t	-1.7459	2.26E-06	3.30E-05	
ENSG00000	HIST1H4H	histone clus	1.2954	2.27E-06	3.30E-05	GO:0003723
ENSG00000	EGR1	early growth	-1.8912	2.31E-06	3.36E-05	GO:0010628
ENSG00000	AC025178.1	novel transc	2.2392	2.39E-06	3.46E-05	
Novel.27404	-	-	2.3777	2.42E-06	3.50E-05	
ENSG00000	B4GALNT4	beta-1,4-N-	-1.0151	2.46E-06	3.55E-05	GO:0016020
ENSG00000	AC117382.1	DnaJ (Hsp4C	1.937	2.47E-06	3.57E-05	
ENSG00000	ALDH2	aldehyde de	-1.1924	2.49E-06	3.59E-05	GO:0016491
ENSG00000	AL360270.2	novel transc	1.906	2.73E-06	3.92E-05	
ENSG00000	PCDHA9	protocadher	2.0918	2.81E-06	4.02E-05	GO:0016020
ENSG00000	AL121989.1	novel transc	2.9347	2.88E-06	4.11E-05	
ENSG00000	AQP11	aquaporin 1	1.0544	2.89E-06	4.12E-05	GO:0016020
ENSG00000	SNCG	synuclein ga	-1.7963	2.94E-06	4.18E-05	GO:0005737
ENSG00000	AC100821.1	ESF1, nucleo	1.1332	3.03E-06	4.30E-05	
ENSG00000	MTND2P2	MT-ND2 pse	3.7308	3.04E-06	4.31E-05	
ENSG00000	C2CD4C	C2 calcium c	-1.1745	3.10E-06	4.39E-05	
ENSG00000(	CDHR5	cadherin rel	1.1245	3.18E-06	4.50E-05	GO:0016020
ENSG00000	P2RY14	purinergic re	2.423	3.32E-06	4.68E-05	GO:0016020
ENSG00000	HIST1H2BI	histone clus	2.1876	3.32E-06	4.68E-05	GO:0005634
ENSG00000	PCDHGA10	protocadher	1.4269	3.39E-06	4.76E-05	GO:0016020
ENSG00000	LAGE3	L antigen fa	-1.0722	3.40E-06	4.78E-05	GO:0005634
ENSG00000	NME3	NME/NM23	-1.2092	3.59E-06	5.02E-05	GO:0046872
ENSG00000	MYO7B	myosin VIIB	-1.4534	3.59E-06	5.03E-05	GO:0005737
ENSG00000	GAPDHP61	glyceraldehy	2.6453	3.61E-06	5.04E-05	
ENSG00000	ANGPTL3	angiopoietin	1.6901	3.75E-06	5.22E-05	GO:0005615
ENSG00000	AC131971.1	novel transc	1.553	3.92E-06	5.43E-05	
ENSG00000	AC130895.1	novel transc	2.0772	3.94E-06	5.46E-05	
ENSG00000	AC068025.2	novel transc	1.6417	4.03E-06	5.58E-05	
ENSG00000	LAMTOR5-A	LAMTOR5 a	1.1534	4.06E-06	5.61E-05	
ENSG00000	AC127024.8	TEC	1.1571	4.10E-06	5.65E-05	
ENSG00000	AC130650.2	novel transc	1.3757	4.15E-06	5.72E-05	
ENSG00000	BICRA-AS1	BICRA antise	2.4922	4.16E-06	5.73E-05	
ENSG00000	AC009948.5	novel transc	2.5656	4.20E-06	5.78E-05	
ENSG00000	HIST1H3B	histone clus	2.5125	4.78E-06	6.51E-05	GO:0005634
ENSG00000	BIRC6-AS2	BIRC6 antise	2.0858	4.86E-06	6.61E-05	
ENSG00000	FOS	Fos proto-o	-1.19	4.86E-06	6.61E-05	GO:0007179
ENSG00000	C7orf31	chromosom	-1.0153	4.89E-06	6.65E-05	GO:0005737
ENSG00000	CNTN2	contactin 2	2.0834	4.96E-06	6.74E-05	GO:0016020

ENSG000002	AL117381.1	novel transc	2.087	4.96E-06	6.74E-05	
ENSG000001	ZNF660	zinc finger p	1.7762	5.01E-06	6.80E-05	GO:0003676
ENSG000001	LRRC4	leucine rich	1.6185	5.10E-06	6.92E-05	GO:0016020
ENSG000001	LRRC20	leucine rich	-1.0309	5.23E-06	7.07E-05	GO:0005515
ENSG000002	AL139317.5	novel transc	1.7088	5.39E-06	7.27E-05	
ENSG000002	AL133243.4	TEC	1.5616	5.44E-06	7.34E-05	
ENSG000001	TIGD5	tigger transp	-1.0213	5.44E-06	7.34E-05	GO:0003676
ENSG000002	AC022973.3	novel transc	1.688	5.55E-06	7.46E-05	
ENSG000002	AC092139.2	novel transc	1.8848	5.73E-06	7.68E-05	
ENSG000002	AC016727.3	novel transc	1.403	5.92E-06	7.90E-05	
Novel.27423	-	-	1.8	6.08E-06	8.11E-05	
ENSG000002	ZNF564	zinc finger p	1.3868	6.12E-06	8.14E-05	GO:0003676
ENSG000002	RPL12P20	ribosomal p	2.3302	6.23E-06	8.26E-05	
ENSG000002	AL133284.1	novel transc	2.4339	6.63E-06	8.74E-05	
ENSG000001	UBE2Q2P1	ubiquitin co	1.2417	6.68E-06	8.80E-05	
ENSG000002	AC005920.2	novel transc	1.9574	6.82E-06	8.97E-05	
ENSG000001	SLC10A1	solute carrie	2.0449	6.85E-06	9.00E-05	GO:0016020
ENSG000001	PSG9	pregnancy s	1.0746	6.90E-06	9.04E-05	GO:0005576
ENSG000001	NXPH4	neurexophil	-1.2552	6.94E-06	9.09E-05	GO:0005576
ENSG000002	SERF1B	small EDRK-	-1.7747	6.99E-06	9.16E-05	GO:0003674
ENSG000001	SPARCL1	SPARC like 1	2.9718	7.06E-06	9.24E-05	GO:0005615
ENSG000002	HSPD1P11	heat shock p	1.3277	7.38E-06	9.62E-05	
ENSG000002	ST7-AS2	ST7 antisens	1.8809	7.46E-06	9.72E-05	
ENSG000001	WNT7B	Wnt family r	-1.9433	7.59E-06	9.88E-05	GO:0005102
Novel.27450	-	-	3.6506	7.84E-06	0.00010169	
ENSG000001	FAM19A2	family with s	1.7777	7.89E-06	0.0001022	GO:0005737
ENSG000002	AC064874.1	novel transc	2.5533	7.92E-06	0.00010259	
ENSG000001	FAM20A	FAM20A, gc	1.6471	7.98E-06	0.00010318	GO:0005783
ENSG000002	AC092139.3	novel transc	1.4078	8.11E-06	0.00010473	
ENSG000002	HIST1H3I	histone clus	2.1709	8.27E-06	0.0001066	GO:0005634
ENSG000002	AC134349.2	taste recept	2.0582	8.42E-06	0.00010837	
ENSG000001	MEGF11	multiple EGI	1.9091	8.48E-06	0.00010916	GO:0016020
ENSG000002	PCDHGB7	protocadher	2.2998	8.58E-06	0.00011028	GO:0016020
ENSG000000	PANX2	pannexin 2	-1.1035	8.73E-06	0.00011202	GO:0016020
ENSG000001	CXCL17	C-X-C motif	2.0594	8.83E-06	0.0001131	GO:0005515
ENSG000002	AC079915.1	novel transc	1.4142	8.90E-06	0.00011394	
ENSG000002	AC104332.1	novel pseuc	2.4594	8.94E-06	0.00011437	
ENSG000001	ZNF460	zinc finger p	1.3123	9.09E-06	0.00011612	GO:0003676
ENSG000001	TCEAL1	transcriptior	-1.1311	9.16E-06	0.00011703	GO:0005634
ENSG000002	MIR133A1HC	MIR133A1 h	1.734	9.18E-06	0.00011711	
Novel.5902	-	-	1.2922	9.18E-06	0.00011711	
ENSG000001	TOX2	TOX high m	-1.1831	9.25E-06	0.00011788	GO:0005634
ENSG000002	ERI3-IT1	ERI3 intronic	2.3133	9.28E-06	0.00011818	
ENSG000002	AC087257.1	novel transc	1.7866	9.36E-06	0.00011902	
ENSG000001	C11orf65	chromosom	1.3379	9.72E-06	0.00012337	GO:0005515
ENSG000001	C1orf122	chromosom	-1.1842	9.83E-06	0.00012465	
ENSG000002	AC016027.2	novel transc	1.4798	1.00E-05	0.00012666	
ENSG000002	AC234772.2	novel transc	1.7555	1.07E-05	0.00013442	

ENSG000002	AC021205.3	novel transc	1.5094	1.11E-05	0.00013865	
ENSG000002	AP002833.1	novel transc	1.5624	1.13E-05	0.00014045	
ENSG000001	KREMEN2	kringle cont	-1.444	1.13E-05	0.00014125	GO:0016020
ENSG000002	AC103810.5	novel transc	1.8114	1.14E-05	0.00014125	
ENSG000000	ANGPT2	angiopoietir	2.2882	1.18E-05	0.00014656	GO:0046872
ENSG000002	AC133644.2	novel transc	2.1029	1.19E-05	0.00014703	
ENSG000002	AC009054.2	novel transc	1.4585	1.19E-05	0.00014751	
ENSG000002	AL353748.2	developmer	2.8779	1.19E-05	0.00014791	
ENSG000002	BACH1-IT1	BACH1 intrc	1.294	1.25E-05	0.00015447	
ENSG000002	AL353804.1	novel transc	1.632	1.28E-05	0.00015699	
ENSG000002	AC005096.1	novel transc	1.9129	1.28E-05	0.00015728	
ENSG000002	AC063965.1	novel transc	2.2414	1.30E-05	0.00015974	
ENSG000002	CISD3	CDGSH iron	-1.019	1.34E-05	0.0001637	GO:0046872
ENSG000002	PEG13	paternally e:	1.256	1.37E-05	0.00016674	
ENSG000002	AL021328.1	uncharacteri	2.5773	1.39E-05	0.00016949	
ENSG000002	AL138828.1	novel transc	1.5422	1.40E-05	0.00017015	
ENSG000002	MIR1207	microRNA 1	2.3694	1.41E-05	0.00017152	
ENSG000002	RN7SL285P	RNA, 7SL, cy	1.9575	1.47E-05	0.0001778	
ENSG000002	AC105429.1	novel transc	1.3307	1.49E-05	0.00018021	
ENSG000001	ANGPTL7	angiopoietir	2.1639	1.57E-05	0.00018897	GO:0005515
ENSG000002	LBH	limb bud an	-1.3111	1.61E-05	0.00019325	GO:0005634
ENSG000002	AC011451.2	UBX domair	2.0494	1.72E-05	0.00020435	
ENSG000002	AC007496.3	novel transc	1.6455	1.75E-05	0.00020796	
ENSG000001	ELAVL2	ELAV like RN	1.2054	1.76E-05	0.00020898	GO:0003676
ENSG000002	AC010320.4	novel transc	3.1031	1.76E-05	0.00020946	
ENSG000002	MIR181A1H(	MIR181A1 h	3.0915	1.77E-05	0.00020965	
ENSG000002	AC010287.1	chromosom	1.9369	1.78E-05	0.00021057	
ENSG000001	CACNA2D4	calcium volt	-1.1779	1.80E-05	0.00021329	GO:0016020
ENSG000002	AL513327.1	novel transc	1.0164	1.81E-05	0.00021423	
ENSG000002	AC023509.6	-	2.0024	1.85E-05	0.00021876	
ENSG000002	AC027288.1	novel transc	2.8868	1.86E-05	0.00021974	
ENSG000002	RN7SL128P	RNA, 7SL, cy	2.2078	2.03E-05	0.00023765	
ENSG000002	AC013394.1	novel protei	1.7866	2.03E-05	0.00023765	
ENSG000002	HIST1H4B	histone clus	1.8916	2.07E-05	0.00024182	GO:0003723
ENSG000000	PTPRN	protein tyro	-1.1621	2.12E-05	0.00024677	GO:0005634
ENSG000002	BMS1P2	BMS1, ribos	1.1137	2.14E-05	0.0002486	
ENSG000000	MTMR7	myotubular	1.3162	2.24E-05	0.00025969	GO:0005737
ENSG000000	SOX8	SRY-box 8	-1.0703	2.28E-05	0.00026393	GO:0005634
ENSG000002	E2F3-IT1	E2F3 introni	1.645	2.29E-05	0.00026525	
ENSG000001	HACD4	3-hydroxyac	1.7709	2.42E-05	0.00027827	GO:0016020
ENSG000001	ABHD11	abhydrolase	-1.1126	2.42E-05	0.00027861	GO:0016787
ENSG000001	NMNAT2	nicotinamid	-1.1735	2.48E-05	0.00028448	GO:0005737
ENSG000002	AC092910.1	presenilin as	2.6207	2.60E-05	0.00029704	
ENSG000002	AC092574.2	novel transc	2.0299	2.60E-05	0.00029747	
ENSG000001	FIBCD1	fibrinogen C	-1.1619	2.62E-05	0.0002992	GO:0016020
ENSG000001	CORO1A	coronin 1A	-1.4567	2.62E-05	0.0002992	GO:0051015
ENSG000002	AP002495.1	Putative shc	1.0388	2.64E-05	0.00030114	GO:0005634
ENSG000002	FAM204CP	family with s	3.9945	2.66E-05	0.00030345	

ENSG000002	PCDHA10	protocadher	1.4345	2.70E-05	0.00030682	GO:0016020
ENSG000002	AC079866.2	novel transc	1.9553	2.70E-05	0.00030704	
ENSG000002	AL024474.2	novel transc	2.2243	2.72E-05	0.00030898	
ENSG000002	AL132777.1	MAD2 mitot	1.6712	2.74E-05	0.00031078	
ENSG000001	BMPER	BMP bindin	-1.3159	2.79E-05	0.00031668	GO:0005615
ENSG000001	CXCL8	C-X-C motif	-1.6111	2.84E-05	0.00032195	GO:0005622
ENSG000002	AL138847.2	novel transc	2.023	2.88E-05	0.00032603	
ENSG000001	LYSMD2	LysM domai	-1.5326	2.89E-05	0.00032666	
Novel.21056	-	-	1.2355	2.92E-05	0.00033036	
ENSG000002	TMLHE-AS1	TMLHE antis	1.895	2.95E-05	0.00033317	
Novel.27426	-	-	6.1382	2.97E-05	0.00033485	
ENSG000002	AC016590.2	novel transc	1.5176	3.02E-05	0.00033969	
ENSG000002	LINC01963	long interge	-1.0668	3.12E-05	0.00035054	
ENSG000002	AC125611.2	FGFR1 onco	1.5015	3.12E-05	0.00035073	
ENSG000002	CHMP4BP1	charged mu	1.5957	3.21E-05	0.00035981	
ENSG000002	AC125612.1	small nuclea	2	3.25E-05	0.00036442	
ENSG000002	AL133444.1	uncharacteri	1.6051	3.28E-05	0.00036758	
ENSG000002	AC137055.1	interferon ir	1.6205	3.39E-05	0.00037769	
ENSG000002	C3orf67-AS1	C3orf67 anti	4.0001	3.53E-05	0.00039242	
ENSG000002	SMAD1-AS2	SMAD1 antis	3.1041	3.59E-05	0.00039895	
ENSG000002	KRTAP2-3	keratin asso	-3.685	3.68E-05	0.00040715	GO:0005515
ENSG000002	AC131009.4	novel transc	1.2759	3.72E-05	0.00041064	
ENSG000002	AC006504.1	novel transc	1.3128	3.74E-05	0.00041256	
ENSG000002	BNIP3P11	BCL2 interac	1.3438	3.74E-05	0.0004126	
ENSG000002	AC013549.2	novel transc	2.0428	3.77E-05	0.00041535	
ENSG000002	AC021087.2	novel transc	1.3341	3.83E-05	0.00042114	
ENSG000002	DDX18P1	DEAD-box h	1.5724	3.87E-05	0.00042478	
ENSG000002	AC104162.2	novel transc	1.2805	3.90E-05	0.00042757	
ENSG000001	PALM3	paralemmin	-1.0509	3.94E-05	0.00043119	GO:0005737
ENSG000002	FP236383.3	novel transc	2.5457	4.05E-05	0.00044282	
ENSG000002	FO680682.1	novel transc	2.5812	4.13E-05	0.00045069	
ENSG000002	BNIP3P40	BCL2 interac	2.3069	4.21E-05	0.00045891	
ENSG000002	AL139274.1	novel TWIST	1.9733	4.27E-05	0.00046535	
ENSG000001	PRSS48	serine prote	1.3711	4.28E-05	0.00046642	GO:0016787
ENSG000002	SETP10	SET pseudog	2.3623	4.28E-05	0.00046655	
ENSG000002	AC008147.4	novel pseuc	2.0725	4.30E-05	0.00046755	
ENSG000002	AL139294.1	novel transc	2.5448	4.37E-05	0.00047517	
ENSG000002	TATDN2P2	TatD DNase	1.2201	4.39E-05	0.0004769	
ENSG000002	Z97198.1	novel transc	1.3652	4.40E-05	0.00047796	
ENSG000001	ARHGDIB	Rho GDP dis	-1.1044	4.43E-05	0.00048011	GO:0005737
ENSG000001	GPX2	glutathione	1.4997	4.43E-05	0.00048026	GO:0055114
ENSG000002	SNX18P3	sorting nexi	1.0313	4.45E-05	0.00048197	
ENSG000002	MTND5P26	MT-ND5 pse	2.5678	4.56E-05	0.00049198	
ENSG000002	AL138689.2	novel transc	1.8145	4.56E-05	0.00049211	
Novel.15561	-	-	-1.0594	4.58E-05	0.00049425	
ENSG000002	CERS1	ceramide syi	-1.1024	4.61E-05	0.00049686	GO:0016020
ENSG000002	AP000577.1	TEC	1.7839	4.67E-05	0.00050186	
ENSG000002	AL049637.1	uncharacteri	1.656	4.70E-05	0.0005051	

ENSG000002	AF127577.2	novel transc	1.8339	4.73E-05	0.0005076	
ENSG000002	ZNF75BP	zinc finger p	2.8733	4.79E-05	0.00051301	
ENSG000002	ZBED3-AS1	ZBED3 antis	1.1891	4.80E-05	0.00051388	
ENSG000002	AC010200.1	phosphatidy	1.419	4.94E-05	0.00052593	
ENSG000002	AC104984.3	novel transc	1.7931	4.95E-05	0.00052739	
ENSG000001	SMIM29	small integra	-1.0085	4.99E-05	0.00053133	GO:0016020
ENSG000002	CLIP1-AS1	CLIP1 antise	1.4642	5.02E-05	0.00053373	
ENSG000001	MAPK15	mitogen-ac	-1.3694	5.03E-05	0.00053487	GO:0005634
ENSG000001	DCHS1	dachsous ca	-1.0023	5.19E-05	0.00055035	GO:0005622
ENSG000002	RPS20P15	ribosomal p	2.207	5.20E-05	0.0005512	
ENSG000002	AC016027.3	novel transc	1.7364	5.23E-05	0.00055329	
ENSG000002	AC108688.1	DnaJ (Hsp40	2.2406	5.25E-05	0.00055499	
ENSG000002	TYW1B	tRNA-yW sy	1.2588	5.32E-05	0.00056152	GO:0016020
ENSG000002	MTCYBP18	MT-CYB pse	2.5996	5.33E-05	0.00056316	
ENSG000002	AL450226.1	uncharacteri	2.7047	5.40E-05	0.00056907	
ENSG000002	RF00424	-	5.9304	5.40E-05	0.00056951	
ENSG000002	CEACAMP10	carcinoembr	1.0651	5.46E-05	0.00057513	
ENSG000002	AC087392.3	novel transc	1.5105	5.50E-05	0.00057813	
ENSG000002	HNRNPA1P.	heterogene	1.6164	5.52E-05	0.00058029	
Novel.27425	-	-	2.9575	5.52E-05	0.00058042	
ENSG000002	RNU6-711P	RNA, U6 sm	2.4038	5.53E-05	0.00058147	
ENSG000002	AL645568.1	uncharacteri	1.6583	5.63E-05	0.00059	
ENSG000002	KRT18P57	keratin 18 ps	2.341	5.67E-05	0.00059421	
ENSG000002	KNOP1P2	lysine rich n	1.9678	5.71E-05	0.00059737	
ENSG000002	AC011815.1	novel transc	1.5433	5.82E-05	0.00060777	
ENSG000002	AC097493.2	ribosomal p	1.9019	6.00E-05	0.000624	
ENSG000002	KANSL1-AS	KANSL1 ant	1.8497	6.07E-05	0.0006303	
ENSG000002	AC016590.1	novel transc	1.5187	6.11E-05	0.00063428	
ENSG000002	AL162578.1	ribosomal p	2.1641	6.16E-05	0.00063952	
ENSG000002	AC008895.1	TEC	1.2893	6.21E-05	0.00064394	
ENSG000002	AC011447.5	BCL2/adenc	2.4651	6.36E-05	0.00065888	
Novel.17494	-	-	2.4898	6.59E-05	0.0006793	
ENSG000002	ANKRD44-I	ANKRD44 in	2.0385	6.63E-05	0.00068216	
ENSG000002	AC027018.1	novel transc	2.1112	6.96E-05	0.00071322	
ENSG000000	RGPD5	RANBP2-lik	1.2095	6.98E-05	0.00071546	GO:0005737
ENSG000002	PTTG4P	pituitary tur	2.4786	7.18E-05	0.00073368	
ENSG000002	PLCE1-AS1	PLCE1 antise	2.3401	7.24E-05	0.00073892	
ENSG000001	TMEM200A	transmembr	-1.1384	7.26E-05	0.00074031	GO:0016020
ENSG000001	LIF	LIF, interleu	-1.0075	7.30E-05	0.000744	GO:0005615
ENSG000001	SRP72P2	signal recog	2.1239	7.37E-05	0.00074952	
ENSG000002	AC005072.1	novel transc	1.2822	7.45E-05	0.00075726	
ENSG000002	AL096869.1	novel transc	4.2085	7.48E-05	0.00076019	
ENSG000002	AL590762.4	pseudogene	2.1164	7.52E-05	0.00076345	
ENSG000002	LINC02595	long interge	2.1795	7.70E-05	0.00077948	
ENSG000001	LRRC19	leucine rich	1.5416	7.73E-05	0.00078236	GO:0016020
ENSG000002	AL512326.1	UDP-Gal:be	1.9699	7.80E-05	0.00078877	
ENSG000002	AC083964.2	TEC	1.9718	7.97E-05	0.00080472	
ENSG000002	PRR13P1	proline rich	2.6758	8.13E-05	0.00081986	

ENSG000001	ZFHX2-AS1	ZFHX2 antis	1.3417	8.17E-05	0.0008234	
ENSG000002	AL353804.2	novel transc	1.7262	8.19E-05	0.00082557	
ENSG000001	ASB18	ankyrin repe	1.449	8.20E-05	0.00082615	GO:0016567
ENSG000002	SMC5-AS1	SMC5 antise	1.7103	8.25E-05	0.00083005	
ENSG000002	USP17L7	ubiquitin sp	4.7956	8.27E-05	0.00083153	GO:0005634
ENSG000002	MTCO1P28	MT-CO1 pse	1.3301	8.46E-05	0.00085009	
ENSG000002	AC012618.2	ribosomal p	1.4529	8.53E-05	0.00085629	
Novel.7366	-	-	1.9909	8.59E-05	0.00086176	
ENSG000002	EIF2S2P5	eukaryotic t	3.2367	8.70E-05	0.0008713	
Novel.5901	-	-	1.3662	8.75E-05	0.00087535	
ENSG000002	TCEA1P4	transcriptior	1.9109	8.76E-05	0.00087577	
ENSG000002	AC022973.1	ribosomal p	2.6716	8.77E-05	0.0008765	
ENSG000002	DIP2A-IT1	DIP2A intror	1.3046	8.85E-05	0.00088386	
ENSG000002	AF107885.2	novel transc	1.5327	8.86E-05	0.00088402	
ENSG000002	AC022022.2	leucine rich	2.4003	9.13E-05	0.00090825	
ENSG000002	AC002429.2	novel transc	2.3338	9.17E-05	0.00091059	
ENSG000002	AC009245.1	ribosomal p	-1.0618	9.16E-05	0.00091059	
ENSG000002	AC015845.1	novel transc	2.0851	9.21E-05	0.00091409	
ENSG000002	MFSD1P1	major facilit	1.5879	9.36E-05	0.00092756	
ENSG000002	MTRNR2L4	MT-RNR2 lil	1.2234	9.42E-05	0.00093291	GO:0005737
ENSG000002	AC012568.1	novel transc	1.9784	9.47E-05	0.0009371	
ENSG000002	AC131571.1	novel transc	2.1414	9.51E-05	0.00093927	
ENSG000001	ARSI	arylsulfatase	-1.0478	9.67E-05	0.0009525	GO:0046872
ENSG000002	AC092135.1	novel transc	2.5645	9.79E-05	0.0009635	
ENSG000001	INHBE	inhibin subu	-1.2402	9.86E-05	0.00096967	GO:0008083
ENSG000002	AC097493.1	ribosomal p	2.3613	9.98E-05	0.00097912	
ENSG000002	AC241520.1	zinc finger p	2.4307	9.98E-05	0.00097912	
ENSG000001	HMCN1	hemicentin	1.5649	9.98E-05	0.00097918	GO:0005737
ENSG000002	AC105235.1	TEC	1.4393	0.00010149	0.00099387	
ENSG000002	PHBP12	prohibitin p	1.8162	0.00010212	0.00099929	
ENSG000002	AC089984.2	MRS2 magn	1.9027	0.00010358	0.0010124	
ENSG000002	AL138820.1	novel transc	1.9924	0.00010431	0.0010191	
ENSG000002	AC110813.1	uncharacteri	2.357	0.00010523	0.0010266	
ENSG000002	RPL3P6	ribosomal p	1.6195	0.0001069	0.0010412	
ENSG000001	ARMC5	armadillo re	-1.0451	0.00010717	0.0010436	GO:0005829
ENSG000002	LRRC69	leucine rich	1.2465	0.00010927	0.0010613	
ENSG000002	AC079866.1	CTAGE fami	1.7097	0.00010989	0.0010666	
ENSG000001	C2CD6	C2 calcium c	1.9091	0.00010994	0.0010667	GO:0005515
ENSG000002	AC091057.4	novel transc	1.3743	0.00011061	0.0010723	
ENSG000001	RAB20	RAB20, mem	-1.5479	0.00011294	0.0010929	GO:0016020
ENSG000002	AC004865.1	zinc finger p	2.3844	0.00011313	0.0010944	
ENSG000002	CEBPD	CCAAT enha	-1.2092	0.00011696	0.0011284	GO:0005634
ENSG000001	HIST1H4C	histone clus	1.9171	0.00011884	0.0011444	GO:0003723
ENSG000001	RTN2	reticulon 2	-1.2002	0.0001199	0.0011537	GO:0016020
ENSG000002	DIAPH3-AS2	DIAPH3 anti	1.9816	0.0001207	0.0011608	
ENSG000002	RPS3AP14	ribosomal p	4.137	0.00012128	0.001166	
Novel.27428	-	-	2.5143	0.0001222	0.0011736	
ENSG000001	STX19	syntaxin 19	1.8899	0.00012311	0.0011807	GO:0005737

ENSG000002	AC114402.2	SNF7 domai	2.1532	0.00012364	0.0011849	
ENSG000001	ARC	activity regu	-2.6547	0.00012659	0.0012093	GO:0003723
ENSG000001	OR2AK2	olfactory rec	3.7757	0.0001276	0.0012186	GO:0016020
ENSG000002	RN7SL4P	RNA, 7SL, cy	1.8547	0.00012764	0.0012187	
ENSG000002	AC027763.1	Spi-C transc	2.5747	0.00012895	0.0012299	
ENSG000002	RF00212	small nuclec	3.1817	0.00013254	0.0012616	
ENSG000002	AL049539.1	novel transc	2.3046	0.00013311	0.0012665	
ENSG000002	AC037487.1	novel transc	2.2034	0.00013486	0.0012822	
ENSG000002	AL139039.2	ribosomal p	2.909	0.00013523	0.0012847	
ENSG000002	SETP5	SET pseudog	1.8915	0.00013639	0.0012943	
ENSG000002	RPS27AP12	ribosomal p	1.8109	0.00013716	0.0013009	
ENSG000001	PTPRN2	protein tyro	-1.4752	0.00013779	0.0013058	GO:0016020
ENSG000002	AL133370.1	novel transc	1.7501	0.00013812	0.0013083	
ENSG000002	RSF1-IT2	RSF1 introni	2.4782	0.00014118	0.001334	
Novel.27395	-	-	-1.3113	0.0001414	0.0013354	
ENSG000002	AC005343.1	novel transc	1.254	0.0001426	0.0013457	
ENSG000002	KRR1P1	KRR1, small	1.7005	0.00014349	0.0013527	
ENSG000002	CDC42EP3P1	CDC42 effec	4.1588	0.00014427	0.001359	
ENSG000002	EEF1A1P25	eukaryotic t	2.3054	0.00014467	0.001362	
ENSG000000	ROGDI	rogdi homo	-1.0706	0.00014471	0.001362	GO:0005634
ENSG000001	PDZK1	PDZ domair	1.1821	0.00014565	0.0013698	GO:0016020
ENSG000002	AC011379.1	novel transc	1.9	0.0001468	0.0013785	
ENSG000002	AL359094.1	novel transc	2.0652	0.00014918	0.001399	
ENSG000001	CRYAB	crystallin alp	-1.5468	0.00014949	0.0014011	GO:0005634
ENSG000002	ATP1B1P1	ATPase Na+	2.8948	0.00014966	0.0014024	
ENSG000001	DUSP9	dual specific	-1.5672	0.00015012	0.0014057	GO:0005737
ENSG000002	AL731563.3	novel transc	1.1397	0.00015013	0.0014057	
ENSG000002	AC021205.1	ribosomal p	1.9075	0.00015021	0.0014061	
ENSG000002	AC008507.2	novel transc	2.2421	0.00015214	0.0014226	
ENSG000001	LRRN3	leucine rich	1.7933	0.00015326	0.001432	GO:0016020
Novel.27422	-	-	1.5222	0.00015334	0.001432	
ENSG000001	MLPH	melanophili	-1.1798	0.00015518	0.0014454	GO:0005622
ENSG000002	AC091925.2	TEC	3.0186	0.00015518	0.0014454	
ENSG000002	AC005921.3	TEC	1.1861	0.0001561	0.0014521	
ENSG000002	AC005692.1	novel transc	5.8393	0.00016145	0.0014952	
ENSG000001	RNF113A	ring finger p	-1.1266	0.00016191	0.0014984	GO:0046872
ENSG000002	AC073575.1	novel transc	1.7375	0.00016213	0.0014996	
ENSG000001	GPR183	G protein-cc	2.0496	0.0001621	0.0014996	GO:0016020
ENSG000002	AC027627.1	novel transc	1.4141	0.00016506	0.001522	
ENSG000002	USP3-AS1	USP3 antise	1.2901	0.00016535	0.0015243	
ENSG000002	HSD17B8	hydroxyster	-1.138	0.00016819	0.0015477	GO:0016491
ENSG000002	AL831737.1	ribosomal p	2.6355	0.00016957	0.0015576	
ENSG000002	RNU4-9P	RNA, U4 sm	2.5526	0.00017803	0.001629	
ENSG000002	ATP5PDP4	ATP synthas	1.3524	0.00018048	0.0016484	
ENSG000002	AL080317.1	novel transc	1.3646	0.0001817	0.0016575	
ENSG000002	MAPT-IT1	MAPT intror	2.2465	0.00018279	0.0016661	
ENSG000002	AL139128.1	proteasome	1.8921	0.00018324	0.0016682	
ENSG000002	AL449283.1	GAJ protein	1.6367	0.00018404	0.0016741	

ENSG000002	AL158801.1	ribosomal p	1.6903	0.00018449	0.0016779	
ENSG000002	AL031668.1	novel transc	2.1577	0.00018693	0.0016957	
ENSG000002	RNU1-106P	RNA, U1 sm	1.0689	0.00019255	0.0017431	
ENSG000001	EVA1B	eva-1 homo	-1.0239	0.00019481	0.0017601	GO:0016020
ENSG000002	AC068620.3	novel Myb/S	1.7941	0.00019913	0.0017946	
ENSG000002	AC090833.1	uncharacteri	-1.1016	0.00019929	0.0017955	
ENSG000002	AL133370.2	zinc finger p	2.2819	0.00020107	0.0018089	
ENSG000002	AL079343.1	novel transc	1.2818	0.00020119	0.0018095	
ENSG000002	RPL9P32	ribosomal p	2.339	0.00020213	0.0018175	
ENSG000001	ITGAX	integrin sub	-1.4708	0.00020234	0.001819	GO:0016020
ENSG000002	AL513523.1	adipose diff	1.423	0.00020372	0.00183	
ENSG000002	AC245884.8	novel transc	2.0931	0.00020391	0.0018312	
ENSG000002	AC092120.1	novel transc	2.7104	0.00020684	0.0018543	
ENSG000002	RF00017	-	4.0325	0.00020885	0.0018704	
ENSG000002	AC011939.2	novel transc	2.6744	0.0002102	0.0018811	
ENSG000002	KCTD9P5	potassium c	2.6644	0.00021147	0.001892	
ENSG000002	PTPN2P1	protein tyro	1.8654	0.00021296	0.0019048	
ENSG000002	AC092364.2	cyclin Y like	1.5994	0.00021362	0.0019093	
ENSG000002	AC009464.1	novel transc	3.0426	0.00021434	0.0019153	
ENSG000002	GK3P	glycerol kina	3.462	0.00021637	0.001932	GO:0005737
ENSG000002	NPY6R	neuropeptic	2.4421	0.00021715	0.001937	
ENSG000001	MDFI	MyoD family	-1.0734	0.00021745	0.0019387	GO:0005634
ENSG000002	BNIP3P42	BCL2 interac	2.1325	0.0002196	0.0019564	
ENSG000001	DLL1	delta like ca	-1.0514	0.00022025	0.0019618	GO:0045944
ENSG000002	MIR548K	microRNA 5	3.4924	0.00022119	0.0019696	
ENSG000001	CBR3	carbonyl rec	-1.2412	0.00022134	0.0019704	GO:0005737
ENSG000002	AC134349.1	novel transc	2.7192	0.00022372	0.0019881	
ENSG000000	CBARP	CACN subur	-1.2304	0.00022423	0.0019917	GO:0016020
ENSG000001	OTUD4P1	OTUD4 pseu	3.1912	0.00022728	0.0020148	
ENSG000002	RF00066	-	2.2174	0.00023011	0.0020369	
ENSG000002	AC104984.6	novel transc	1.5298	0.00023027	0.0020378	
ENSG000001	MMP24	matrix meta	1.3004	0.0002319	0.0020497	GO:0016020
ENSG000002	RNU7-175P	RNA, U7 sm	5.6249	0.00023256	0.002055	
ENSG000002	CYP2T1P	cytochrome	1.1665	0.00023706	0.0020927	
ENSG000002	HNRNPDLP:	heterogene	3.3862	0.00023771	0.002098	
ENSG000002	AC007731.4	novel transc	1.7003	0.00023918	0.0021098	
ENSG000002	RF00019	-	3.0963	0.00023927	0.0021101	
ENSG000002	AC002558.1	ribosomal p	1.0569	0.00023964	0.0021129	
ENSG000002	DANT2	DXZ4 associ	1.3542	0.00024181	0.0021294	
ENSG000002	RHEBP3	RHEB pseud	2.2928	0.00024582	0.0021605	
ENSG000002	MAGI1-IT1	MAGI1 intro	2.1829	0.00024965	0.0021892	
ENSG000002	AC027796.5	novel transc	2.1231	0.00025367	0.0022202	
ENSG000002	AC139451.1	PHD finger p	2.0703	0.00025452	0.0022271	
ENSG000002	AC073529.1	novel transc	1.3195	0.00025549	0.0022344	
ENSG000002	AC008649.2	novel transc	1.9606	0.00025565	0.0022354	
ENSG000002	AC109597.1	uncharacteri	1.7387	0.00025778	0.0022523	
ENSG000002	MIR548I2	microRNA 5	2.5867	0.00026331	0.0022934	
ENSG000001	DLEU2L	deleted in ly	1.3488	0.00026551	0.0023108	

ENSG000002	PFN1P1	profilin 1 ps	-1.546	0.00026792	0.0023296	
ENSG000002	RF00017	-	1.6719	0.00026851	0.0023341	
ENSG000002	RTL1	retrotranspo	1.0783	0.0002688	0.0023355	GO:0016020
ENSG000002	HIST1H2AH	histone clus	2.1895	0.00026886	0.0023355	GO:0005634
ENSG000002	AC132068.1	novel transc	3.1793	0.00026924	0.0023382	
ENSG000002	PSG8-AS1	PSG8 antise	1.663	0.00027041	0.0023467	
ENSG000002	AL139022.1	uncharacteri	1.1034	0.00027744	0.0024036	
ENSG000002	AC090971.3	Leo1, Paf1/F	1.432	0.00027808	0.0024079	
ENSG000002	COX6CP13	cytochrome	5.1603	0.00027907	0.0024159	
ENSG000002	AC107982.2	novel transc	2.3923	0.00028015	0.0024225	
Novel.28377	-	-	3.973	0.00028084	0.0024277	
ENSG000002	AL162253.1	novel transc	5.5799	0.0002836	0.0024486	
ENSG000000	GP6	glycoprotein	1.4555	0.00028457	0.0024558	GO:0016020
ENSG000002	PCDHGB5	protocadher	1.7807	0.00029158	0.0025112	GO:0016020
ENSG000002	AC022080.3	mitochondri	2.1149	0.00029318	0.002521	
ENSG000002	AL359697.1	novel transc	2.8051	0.00029513	0.0025341	
ENSG000002	MTND5P2	MT-ND5 pse	3.4314	0.00029759	0.0025546	
ENSG000002	AC023090.2	TEC	1.7003	0.00029785	0.0025562	
ENSG000002	Z95152.1	nudix (nucle	1.1374	0.0002984	0.0025604	
ENSG000002	SCARNA9	small Cajal b	1.6669	0.00029879	0.0025625	
ENSG000002	AC093567.1	novel transc	2.7425	0.00030201	0.002587	
ENSG000002	AGGF1P3	angiogenic f	2.6221	0.00030393	0.0026023	
ENSG000002	LARGE-AS1	LARGE antis	1.6421	0.00030442	0.002604	
ENSG000002	AC087893.1	novel transc	1.8367	0.00031115	0.0026564	
ENSG000002	AC024451.4	novel transc	1.2572	0.00031337	0.0026745	
ENSG000001	CYP2S1	cytochrome	-1.7908	0.00031463	0.0026829	GO:0016020
ENSG000002	AL590627.1	forkhead bo	1.1021	0.00031523	0.0026874	
ENSG000002	AC092747.2	liprin-beta-	2.3205	0.00031677	0.0026986	
ENSG000001	LRRC4B	leucine rich	-1.1231	0.00031861	0.002713	GO:0016020
ENSG000001	C20orf144	chromosom	2.1784	0.00032048	0.0027257	
ENSG000002	KIRREL3-AS	KIRREL3 ant	1.5036	0.00032237	0.0027378	
ENSG000002	ELOCP33	elongin C ps	2.6469	0.00032363	0.0027473	
ENSG000002	SNRPGP10	small nuclea	-1.7008	0.00032455	0.0027544	
ENSG000002	AL590762.3	MADP-1 pro	1.8515	0.00032557	0.0027611	
ENSG000002	AC096564.1	uncharacteri	2.321	0.0003273	0.0027745	
ENSG000002	CEACAMP7	carcinoembr	5.5877	0.00032822	0.0027809	
ENSG000002	WASHC5-AS	WASHC5 an	1.2159	0.00033086	0.0028013	
ENSG000002	RN7SL568P	RNA, 7SL, cy	2.4447	0.00033358	0.002821	
ENSG000002	LINC01085	long interge	-1.0854	0.00033572	0.0028378	
ENSG000002	AC024267.4	uncharacteri	2.0276	0.0003399	0.002871	
ENSG000002	AC100763.1	novel transc	2.0131	0.00035411	0.0029806	
ENSG000002	LINC01537	long interge	-1.7461	0.00035492	0.0029867	
ENSG000002	RNU6-1322I	RNA, U6 sm	2.8307	0.00035753	0.0030065	
ENSG000001	CCDC38	coiled-coil c	2.3765	0.00035844	0.0030128	GO:0005737
ENSG000002	RNU1-87P	RNA, U1 sm	2.3421	0.00036051	0.0030287	
ENSG000002	C6orf163	chromosom	1.0495	0.00036148	0.0030354	GO:0003674
ENSG000002	AC007496.1	novel transc	2.077	0.00036303	0.0030458	
ENSG000002	RF00019	-	1.734	0.00036629	0.0030701	

ENSG000002	UBE2FP1	UBE2F pseu	1.5519	0.00036862	0.003086	
ENSG000002	PCDHA4	protocadher	1.4065	0.00037128	0.0031047	GO:0016020
ENSG000002	AC090953.1	THAP doma	2.1449	0.00037164	0.003107	
ENSG000001	KLC3	kinesin light	-1.2207	0.00037249	0.0031133	GO:0003777
ENSG000002	LINC01004	long interge	1.2968	0.00037346	0.0031195	
ENSG000002	AP003108.4	novel transc	1.8568	0.00037957	0.0031666	
ENSG000002	AC063960.2	novel transc	2.3406	0.00038361	0.0031958	
ENSG000002	AL162171.3	novel transc	1.4462	0.0003854	0.0032092	
ENSG000002	ETF1P2	eukaryotic t	1.4459	0.00039293	0.0032697	
ENSG000002	AL096869.2	uncharacteri	2.7638	0.00039401	0.0032771	
ENSG000002	AC015971.1	novel transc	1.573	0.00039521	0.0032841	
ENSG000002	AC022079.1	novel transc	1.9458	0.00039575	0.003287	
ENSG000002	COL6A6	collagen typ	2.0385	0.00039875	0.0033104	GO:0007155
ENSG000002	AC093225.1	novel zinc fi	2.3286	0.00039923	0.0033128	
ENSG000002	AL591623.1	novel transc	1.7891	0.00039959	0.0033147	
ENSG000002	AC087521.2	novel transc	1.3446	0.00040134	0.0033273	
ENSG000002	AC001226.2	novel protei	1.6584	0.00040805	0.0033781	GO:0005764
ENSG000002	LINC01943	long interge	1.8118	0.00041025	0.0033933	
ENSG000002	AC093591.2	novel transc	1.7203	0.0004128	0.0034121	
ENSG000002	RNU6-595P	RNA, U6 sm	3.1398	0.00041507	0.0034276	
ENSG000002	AC024451.1	ribosomal p	1.9759	0.00041653	0.0034373	
ENSG000002	SARNP	SAP domain	1.2375	0.00041738	0.0034436	GO:0003723
ENSG000002	POU5F1P5	POU class 5	1.5436	0.00041804	0.0034481	
ENSG000002	AC022872.1	novel transc	1.9725	0.00042188	0.0034743	
ENSG000002	AL512604.1	ribosomal p	2.0216	0.00042417	0.0034915	
ENSG000002	AL590556.1	ribosomal p	3.3428	0.00042517	0.0034982	
Novel.26203	-	-	-2.3316	0.00042528	0.0034983	
ENSG000002	AL589986.2	novel transc	2.4878	0.00042941	0.003529	
ENSG000002	AC009054.1	novel transc	1.5161	0.00042932	0.003529	
ENSG000001	FAM186A	family with s	1.2453	0.0004365	0.0035782	
ENSG000002	AC068587.2	40S ribosom	2.042	0.00043944	0.0035982	
ENSG000001	HLX	H2.0 like ho	-1.0456	0.00044632	0.0036487	GO:0005634
ENSG000002	RF00017	-	2.5254	0.00044767	0.0036589	
ENSG000001	FAM156B	family with s	2.4352	0.00045664	0.0037254	GO:0035064
ENSG000002	HNRNPA1P	heterogeneo	1.7693	0.00045773	0.0037309	
ENSG000001	ESR2	estrogen rec	1.2812	0.00045956	0.0037441	GO:0005634
ENSG000002	AL031705.1	novel transc	2.3897	0.00046094	0.0037536	
ENSG000002	AC009269.2	novel transc	1.6777	0.00046259	0.0037627	
ENSG000001	TTYH1	tweety famil	1.2278	0.00046268	0.0037627	GO:0016020
ENSG000002	H2AFZP1	H2A histone	2.0656	0.00046482	0.0037766	
ENSG000002	MANEA-DT	MANEA dive	2.466	0.00046691	0.0037927	
ENSG000001	ESPNL	espin like	-1.9416	0.00046719	0.0037942	GO:0003779
ENSG000002	AC138866.1	glucuronida	1.1963	0.0004676	0.0037967	
ENSG000002	SND1-IT1	SND1 intron	1.1857	0.00046824	0.003801	
ENSG000002	AL136984.1	novel transc	1.6488	0.00046922	0.0038081	
ENSG000002	MRTFA-AS1	MRTFA antis	1.2256	0.00047193	0.0038266	
ENSG000000	PKD2L2	polycystin 2	1.6199	0.00047395	0.0038404	GO:0016020
ENSG000002	AL121978.1	microtubule	2.9536	0.00047479	0.0038463	

ENSG000002	ODCP	ornithine de	1.4776	0.00047773	0.003864	
ENSG000002	AC032044.2	TEC	1.8982	0.0004779	0.0038645	
ENSG000002	AC092653.2	TEC	2.4428	0.00048122	0.0038879	
ENSG000002	TATDN1P1	TatD DNase	1.982	0.00048749	0.0039322	
ENSG000002	SRRM1P3	serine/argin	1.3033	0.00048776	0.0039327	
ENSG000002	AC016727.1	novel transc	1.1653	0.00049352	0.0039747	
ENSG000001	ACSBG2	acyl-CoA sy	1.0646	0.00049762	0.0040041	GO:0003824
ENSG000002	AC079601.2	fragile X me	1.7352	0.00050322	0.0040446	
ENSG000001	LRRC70	leucine rich	3.1367	0.00050673	0.0040692	GO:0016020
ENSG000001	SLC4A5	solute carrie	1.8719	0.00050733	0.0040731	GO:0016020
ENSG000002	HIST1H2AJ	histone clus	2.0251	0.00051031	0.0040952	GO:0005634
ENSG000002	AIMP1P2	aminoacyl tl	1.6702	0.00051094	0.0040993	
ENSG000002	AL139120.1	novel transc	2.1524	0.00051352	0.0041173	
ENSG000002	MTND1P8	MT-ND1 pse	1.3062	0.00051791	0.0041469	
ENSG000002	AC010480.1	TEC	2.6972	0.00052137	0.0041718	
ENSG000001	PTGIR	prostagland	-1.0103	0.00052491	0.0041954	GO:0048662
ENSG000002	AGAP1-IT1	AGAP1 intrc	1.4589	0.00053468	0.004264	
ENSG000002	AC067817.1	novel transc	2.0355	0.00053788	0.0042885	
ENSG000002	RGS5	regulator of	1.1242	0.00053942	0.0042989	
ENSG000002	AC009716.2	TEC	4.4149	0.00054366	0.0043279	
ENSG000002	SNORA46	small nuclec	1.7561	0.00054835	0.0043633	
ENSG000002	TIMM9P2	TIMM9 pseu	2.1701	0.00055214	0.0043896	
ENSG000002	HDAC2-AS2	HDAC2 and	1.3055	0.00055846	0.0044299	
ENSG000002	RPS3AP44	ribosomal p	4.4068	0.00056158	0.0044518	
ENSG000002	AC016493.1	uncharacteri	2.3187	0.00056288	0.0044601	
ENSG000002	AC092338.1	novel transc	2.2031	0.00056931	0.0045041	
ENSG000002	TTLL7-IT1	TTLL7 intror	2.2921	0.00057072	0.0045141	
ENSG000002	WWOX-AS1	WWOX antis	2.4919	0.00057105	0.0045141	
ENSG000002	AC004943.3	novel transc	2.2462	0.00057209	0.0045211	
ENSG000002	AC233702.7	novel transc	1.8797	0.00057601	0.0045471	
ENSG000002	AC083806.2	novel transc	1.8442	0.00057783	0.0045594	
ENSG000002	AC097638.1	ribosomal p	3.2717	0.00058405	0.0046044	
ENSG000002	AC138028.2	uncharacteri	1.165	0.0005886	0.0046321	
ENSG000002	BNIP3P37	BCL2 interac	1.9472	0.00059564	0.0046814	
ENSG000002	AC016542.3	TEC	2.1274	0.00060722	0.0047629	
ENSG000002	RNU6-875P	RNA, U6 sm	1.2032	0.00061031	0.0047809	
ENSG000002	HSPE1P27	heat shock p	1.6091	0.00061446	0.0048102	
ENSG000000	KCNH2	potassium v	-1.1926	0.00061703	0.0048283	GO:0048471
ENSG000002	RAD17P2	RAD17 checl	1.9592	0.00062306	0.0048691	
ENSG000002	KRT18P31	keratin 18 p:	1.5751	0.00062602	0.004888	
ENSG000002	AC004637.1	novel transc	2.322	0.00062869	0.0049066	
ENSG000002	AC023389.2	RNA termin	1.3738	0.0006312	0.0049252	
ENSG000002	AC146507.2	ribosomal p	2.8219	0.00063511	0.0049524	
ENSG000002	AC023590.1	uncharacteri	2.3481	0.0006435	0.0050114	
ENSG000001	HES6	hes family b	-1.2039	0.00065308	0.0050804	GO:0005634
ENSG000002	RN7SL502P	RNA, 7SL, cy	2.5789	0.00065424	0.0050872	
ENSG000002	RF00100	-	2.884	0.00065439	0.0050873	
ENSG000001	RNA5SP93	RNA, 5S ribc	3.2588	0.00066217	0.0051433	

ENSG000002	AC253536.3	novel transc	1.369	0.00066743	0.0051785	
ENSG000001	HSD11B1L	hydroxyster	-1.3481	0.00067055	0.0052016	GO:0043231
ENSG000002	RN7SL329P	RNA, 7SL, cy	1.3979	0.00067265	0.0052141	
ENSG000001	RF00019	-	3.5197	0.00067675	0.0052416	
ENSG000002	AL445305.1	adaptor-rela	2.4406	0.00068902	0.0053218	
ENSG000001	LHFPL1	LHFPL tetras	5.3796	0.00069531	0.0053647	GO:0016020
ENSG000002	RWDD4P1	RWD domai	1.0968	0.00069547	0.0053647	
ENSG000002	AC096741.1	novel transc	1.7313	0.00069611	0.0053662	
ENSG000002	AC010632.2	novel transc	2.2792	0.00070007	0.0053932	
ENSG000002	RBMS3-AS2	RBMS3 antis	1.4844	0.00070189	0.0054015	
ENSG000001	HIST1H1A	histone clus	2.8625	0.00070429	0.0054188	GO:0005634
ENSG000000	FGF22	fibroblast gr	2.1342	0.00070648	0.0054321	GO:0008083
ENSG000001	RNA5SP195	RNA, 5S ribo	1.7019	0.00070698	0.0054325	
ENSG000002	WASF3-AS1	WASF3 antis	2.2164	0.00070873	0.0054424	
ENSG000002	AC007064.2	novel transc	1.7759	0.00070958	0.0054477	
ENSG000002	AL354943.1	transmembr	2.2708	0.00071177	0.0054623	
ENSG000002	AC009549.1	novel transc	-1.1758	0.00071178	0.0054623	
ENSG000002	AP000560.1	TEC	1.2262	0.00071206	0.0054633	
ENSG000002	AC090559.2	novel transc	1.4189	0.00071429	0.0054783	
ENSG000002	AC007161.1	ribosomal p	2.7615	0.00071815	0.0055041	
ENSG000002	AC007319.1	novel transc	1.1196	0.00071895	0.0055081	
ENSG000002	AC120349.1	novel transc	2.3456	0.00071907	0.0055081	
ENSG000002	AL136304.1	novel transc	2.4816	0.00072024	0.0055143	
ENSG000002	RPSAP13	ribosomal p	1.729	0.00072084	0.0055177	
ENSG000002	AC007038.1	novel transc	1.4743	0.00072767	0.005564	
ENSG000002	AL117379.1	novel transc	1.4269	0.00073041	0.0055806	
ENSG000002	AC110048.2	novel transc	1.0458	0.00073605	0.0056197	
ENSG000002	AL354890.1	ACN9 homo	3.5294	0.00074269	0.005668	
ENSG000002	AC090617.7	novel transc	2.1845	0.00074315	0.0056703	
ENSG000002	AC017002.3	uncharacteri	2.4375	0.00075759	0.0057677	
ENSG000002	AC000120.1	novel transc	1.4343	0.00076462	0.0058155	
ENSG000002	MIR648	microRNA 6	2.0754	0.00076512	0.005818	
ENSG000002	AP003696.1	novel transc	2.1512	0.00076689	0.0058289	
ENSG000002	AC104819.1	trypsin dom	1.4837	0.00076882	0.0058391	
ENSG000002	AC019226.1	TEC	1.9064	0.00077841	0.0059016	
ENSG000002	RN7SL220P	RNA, 7SL, cy	1.4663	0.00077982	0.0059098	
ENSG000002	AL022323.2	novel transc	1.6611	0.0007806	0.0059132	
ENSG000002	AC113155.1	NSA2 ribosc	3.0965	0.00078643	0.005951	
ENSG000002	UBE2CP1	ubiquitin co	1.93	0.00078722	0.0059552	
ENSG000001	MAPK10	mitogen-ac	1.0585	0.00079284	0.0059911	GO:0005622
rylation;tran	hsa:5602	hsa01522;hs	Endocrine resistance ;MA		59.83	28.63
ENSG000002	SNX6P1	sorting nexi	1.8885	0.00079927	0.0060342	
ENSG000002	AL353662.1	ribosomal p	1.8876	0.0008011	0.0060455	
ENSG000001	DEPP1	DEPP1, auto	-1.2536	0.00080617	0.0060812	GO:0005737
ENSG000002	AL137779.3	nucleophos	4.3391	0.00080672	0.006084	
ENSG000002	MTATP6P19	MT-ATP6 ps	2.725	0.00080838	0.0060897	

ENSG000002	DLGAP4-AS	DLGAP4 ant	1.5752	0.00080849	0.0060897	
ENSG000002	RF00019	-	1.8897	0.00080904	0.0060925	
ENSG000001	CCNB3	cyclin B3	1.9391	0.00081279	0.0061161	GO:0005634
ENSG000002	AC104046.1	fatty acid bi	2.1609	0.00081285	0.0061161	
ENSG000002	AC010442.1	mitochondr	2.3827	0.00081677	0.0061404	
ENSG000002	AC006460.1	novel transc	3.7663	0.00081878	0.0061517	
ENSG000002	AC129502.1	TEC	2.0029	0.00082241	0.0061763	
ENSG000002	AC104791.1	novel transc	3.2396	0.00083655	0.0062734	
ENSG000002	MIR2052HG	MIR2052 ho:	5.4234	0.00083729	0.0062769	
ENSG000002	HYMAI	hydatidiforr	1.0731	0.00083877	0.006286	
ENSG000001	MFSD3	major facilit	-1.0123	0.00084212	0.0063058	GO:0016020
ENSG000001	WNT4	Wnt family r	1.6607	0.00084802	0.0063435	GO:0045669
ENSG000002	AC007849.1	novel transc	2.4716	0.00084823	0.0063435	
ENSG000002	RPL26P19	ribosomal p	-1.1415	0.00084819	0.0063435	
ENSG000002	AL365436.1	ribosomal p	2.3911	0.00084979	0.006351	
ENSG000002	AC244093.4	novel transc	1.8591	0.00084991	0.006351	
ENSG000002	AC103769.1	novel transc	1.5751	0.00085623	0.0063928	
ENSG000002	ASTN2-AS1	ASTN2 antis	2.5616	0.00085831	0.006407	
ENSG000002	AC079193.2	novel transc	2.1301	0.00086338	0.0064409	
ENSG000002	AC022173.1	uncharacteri	1.7329	0.00086593	0.0064585	
ENSG000001	SCG2	secretogran	1.1162	0.0008726	0.0065042	GO:2000352
ENSG000002	AL109613.1	novel transc	1.0677	0.00088961	0.0066214	
ENSG000002	KIRREL1-IT1	KIRREL1 intr	1.4932	0.000905	0.0067179	
ENSG000002	AC092120.2	cytidine mo	1.5981	0.00090497	0.0067179	
Novel.26501	-	-	-3.0927	0.00090659	0.0067281	
ENSG000002	KRT18P60	keratin 18 p:	2.975	0.00090773	0.0067338	
ENSG000002	TNPO1P1	transportin	1.7096	0.00090991	0.0067446	
ENSG000001	GNG13	G protein su	1.4774	0.00091902	0.0068049	GO:0016020
ENSG000002	ALOX12P2	arachidonat	1.0055	0.00092556	0.0068476	
ENSG000002	RNU6-787P	RNA, U6 sm	5.3194	0.0009329	0.0068963	
ENSG000002	GAS6-DT	GAS6 diverg	-1.7252	0.00093283	0.0068963	
ENSG000002	KRT18P18	keratin 18 p:	5.3007	0.00093682	0.0069181	
ENSG000002	AC008592.2	novel transc	3.8149	0.00093726	0.0069199	
ENSG000001	RANBP3L	RAN bindin	1.5108	0.00093852	0.0069278	GO:0005634
ENSG000001	NME9	NME/NM23	1.3449	0.00093891	0.0069293	GO:0005737
ENSG000002	AC131934.1	novel transc	1.5085	0.0009398	0.0069344	
ENSG000002	AL356057.1	LSM14A, SC	2.0049	0.00094356	0.0069607	
ENSG000002	AP003110.1	uncharacteri	2.5891	0.00094741	0.0069862	
ENSG000002	RPS2P55	ribosomal p	-1.9019	0.00094933	0.0069975	
ENSG000002	AP006259.1	novel transc	1.5987	0.00095591	0.0070373	
ENSG000002	AC106873.1	novel transc	1.7265	0.00095944	0.0070604	
ENSG000002	AC019072.1	protein tyro	2.9088	0.00097542	0.0071603	
ENSG000002	AC090971.5	novel transc	3.4152	0.00098247	0.0072047	
ENSG000002	RLIMP1	ring finger p	2.3678	0.0009847	0.0072167	
ENSG000002	AC079753.1	TEC	1.3161	0.00098689	0.0072312	
ENSG000002	FP236383.2	novel transc	3.6063	0.00099556	0.0072901	
ENSG000002	RF00019	-	2.4046	0.00099694	0.0072959	
ENSG000002	SERPINH1P1	serpin family	2.171	0.0010067	0.0073581	

ENSG000002	AC135782.3	novel transc	2.0725	0.0010164	0.0074197	
ENSG000002	AL391421.1	novel transc	1.926	0.0010219	0.0074569	
ENSG000002	AC023509.5	zinc finger p	1.9284	0.0010234	0.0074654	
ENSG000002	AC003989.1	pseudogene	2.5484	0.0010284	0.0074989	
ENSG000001	HIST4H4	histone clus	1.7224	0.0010317	0.0075207	GO:0003723
ENSG000001	MYOT	myotilin	1.3839	0.0010329	0.007527	GO:0030018
ENSG000002	RNU6-789P	RNA, U6 sm	2.4247	0.0010387	0.0075676	
ENSG000002	COX6CP1	cytochrome	1.3446	0.0010506	0.0076431	
ENSG000002	AC080188.2	TEC	2.1537	0.0010618	0.0077201	
ENSG000002	ATP11A-AS	ATP11A ant	1.8204	0.0010683	0.0077564	
ENSG000002	DIO1	iodothyroni	1.2865	0.0010699	0.0077647	GO:0016491
ENSG000002	AL583856.1	spermine sy	-1.4758	0.001072	0.0077768	
ENSG000002	TPM3P1	tropomyosi	2.486	0.0010838	0.0078592	
ENSG000002	POC1B-AS1	POC1B antis	1.1419	0.0010852	0.0078675	
ENSG000002	CBWD4P	COBW dom	1.3305	0.001092	0.007914	
ENSG000002	RNU6-1023I	RNA, U6 sm	5.3517	0.0010936	0.0079242	
ENSG000000	CASS4	Cas scaffold	-1.0226	0.0011017	0.0079778	GO:0005737
ENSG000002	AC005042.1	ribosomal p	2.1098	0.0011038	0.0079868	
ENSG000002	AC080013.1	novel transc	1.1372	0.0011041	0.0079868	
ENSG000002	MIR548U	microRNA 5	2.5028	0.0011053	0.0079941	
ENSG000001	CHRNB2	cholinergic r	2.1547	0.0011234	0.0081139	GO:0016020
ENSG000000	CRLF1	cytokine rec	-1.0723	0.0011304	0.0081629	GO:0008284
ENSG000002	AC016245.1	uncharacteri	1.885	0.0011322	0.0081742	
ENSG000002	AL445435.1	sorting nexi	2.1679	0.0011364	0.0081994	
ENSG000002	ITPK1-AS1	ITPK1 antise	1.6655	0.0011387	0.0082143	
ENSG000001	RF00019	-	3.3537	0.0011651	0.0083927	
ENSG000001	COL10A1	collagen typ	1.9331	0.0011674	0.0084053	GO:0046872
ENSG000002	DCDC2B	doublecorti	1.5018	0.0011683	0.0084088	GO:0035556
ENSG000002	PCDHGA12	protocadhe	1.7897	0.0011714	0.008428	GO:0016020
ENSG000002	ATP2A1-AS	ATP2A1 ant	-1.4019	0.0011746	0.0084409	
ENSG000002	AC007151.1	developmer	2.1413	0.0011771	0.0084573	
ENSG000002	AL022097.1	uncharacteri	1.8383	0.0011904	0.0085422	
ENSG000001	FAM83A	family with s	-1.32	0.0012016	0.0086145	GO:0005737
ENSG000002	Z98885.3	novel transc	1.4429	0.001219	0.0087256	
ENSG000002	AC090220.1	novel transc	2.4004	0.0012199	0.0087295	
ENSG000002	AL121652.1	novel transc	3.1455	0.001235	0.0088269	
ENSG000002	EIPR1-IT1	EIPR1 intron	2.2685	0.001243	0.0088805	
ENSG000002	IQCJ-SCHIP1	IQCJ-SCHIP1	2.5892	0.0012456	0.008896	
ENSG000002	RNU4-54P	RNA, U4 sm	3.3699	0.0012474	0.0089068	
ENSG000002	AC022080.1	TAF10 RNA	2.1427	0.0012561	0.008961	
ENSG000002	RGS17P1	regulator of	5.2534	0.0012576	0.0089671	
ENSG000002	AC068580.4	novel protei	1.9895	0.0012628	0.008999	GO:0016020
ENSG000002	C8orf17	chromosom	1.7048	0.0012655	0.0090128	
ENSG000002	PTPRJ-AS1	PTPRJ antise	1.6837	0.0012671	0.009022	
ENSG000002	MPRIP-AS1	MPRIP antis	2.0056	0.0012769	0.0090852	
ENSG000002	AL020995.1	novel transc	1.9202	0.0012779	0.0090905	
ENSG000002	FTH1P4	ferritin heav	1.6008	0.0012814	0.0091096	
ENSG000002	AC009269.5	novel transc	1.4091	0.0012814	0.0091096	

ENSG000001	ZNF29P	zinc finger p	2.3732	0.0012824	0.0091149	
ENSG000001	PTPRQ	protein tyro	1.1455	0.001284	0.0091247	GO:0016787
ENSG000002	AC002543.1	novel pseuc	1.8329	0.0012846	0.009127	
ENSG000002	PCDHGB9P	protocadher	1.6752	0.0012855	0.00913	
ENSG000002	TVP23CP1	TVP23C pseu	2.6402	0.0012869	0.0091345	
ENSG000002	AC005520.1	novel transc	2.0086	0.0012868	0.0091345	
ENSG000002	AC004158.1	novel transc	1.2103	0.0012965	0.0091936	
ENSG000001	CMYA5	cardiomyop	1.7717	0.0013005	0.0092161	GO:0005634
ENSG000002	AC020763.3	novel transc	1.1317	0.0013013	0.0092201	
ENSG000001	SNORC	secondary o	1.3746	0.0013052	0.0092443	GO:0005737
ENSG000002	RAD51AP1P	RAD51 asso	2.4616	0.0013092	0.0092686	
ENSG000002	AC079140.1	novel pseuc	2.9049	0.0013119	0.0092842	
ENSG000002	TSEN15P2	tRNA splicin	2.3869	0.0013212	0.0093427	
ENSG000002	AC092127.2	novel transc	2.4622	0.0013224	0.0093489	
ENSG000001	AMZ1	archaelysin	1.3799	0.0013281	0.00938	GO:0008270
ENSG000002	RPS10P3	ribosomal p	-2.4504	0.0013421	0.0094699	
ENSG000002	AC007014.1	novel transc	2.2808	0.001351	0.0095254	
ENSG000002	AP004289.1	aconitase 2,	2.7783	0.0013759	0.0096763	
ENSG000002	CYCSP29	cytochrome	5.2173	0.0013767	0.0096798	
ENSG000001	CRYM-AS1	CRYM antise	1.1837	0.0013792	0.0096952	
ENSG000002	AC114878.1	pseudogene	4.1479	0.0013891	0.0097497	
ENSG000002	CDC42-IT1	CDC42 intro	1.4188	0.0013935	0.0097738	
ENSG000002	AC012513.2	heterogene	1.4459	0.0014133	0.0099	
ENSG000002	ENPP7P12	ectonucleot	1.8571	0.0014193	0.00993	
ENSG000000	PTGS2	prostagland	-1.2859	0.0014209	0.0099376	GO:0090050
ENSG000002	AC004461.2	DiGeorge sy	1.763	0.0014289	0.0099861	
ENSG000002	AL356599.1	uncharacter	1.0349	0.0014461	0.0101	
ENSG000001	PPL	periplakin	1.2135	0.0014515	0.010132	GO:0005634
ENSG000002	ATP6V0E1P1	ATPase H+ t	2.3717	0.0014584	0.010174	
ENSG000002	TYRO3P	TYRO3P pro	1.5997	0.0014739	0.010274	
ENSG000002	SMG6-IT1	SMG6 intror	2.5965	0.0014763	0.010289	
ENSG000002	RPL23AP53	ribosomal p	1.1379	0.0014769	0.010291	
ENSG000002	PCCA-AS1	PCCA antise	1.6563	0.0014777	0.010294	
ENSG000002	RPL23AP23	ribosomal p	2.2106	0.001486	0.010347	
ENSG000002	AC100850.1	clathrin, ligh	3.6716	0.0014898	0.010365	
ENSG000002	RF00019	-	3.6398	0.0014915	0.010375	
ENSG000002	PHACTR2-A	PHACTR2 ar	1.6289	0.001497	0.010409	
ENSG000002	AC009163.3	novel transc	1.504	0.0015034	0.01045	
ENSG000001	HIST1H2BL	histone clus	1.5134	0.0015065	0.010465	GO:0005634
ENSG000002	AL357552.2	novel transc	2.076	0.001507	0.010467	
ENSG000002	AC008764.1(	TEC	1.6399	0.0015081	0.010472	
ENSG000002	LATS2-AS1	LATS2 antise	1.4374	0.0015244	0.010579	
ENSG000001	FZD8	frizzled class	-1.0228	0.0015308	0.010615	GO:0016020
ENSG000002	RPSAP21	ribosomal p	3.6453	0.0015404	0.010667	
ENSG000002	AC073592.7	novel transc	2.2381	0.0015406	0.010667	
ENSG000001	ATXN7	ataxin 7	1.0524	0.0015438	0.010686	GO:0005634
ENSG000002	AGGF1P1	angiogenic	1.3813	0.001556	0.010759	
ENSG000001	LRFN1	leucine rich	-1.4251	0.0015593	0.010779	GO:0016020

ENSG000001	COL18A1-A	COL18A1 ar	1.3678	0.0015645	0.010809	
ENSG000002	AC124916.2	ribosomal p	1.3849	0.0015719	0.010854	
ENSG000002	AL031770.1	novel transc	1.5057	0.0015786	0.010889	
ENSG000002	BACH1-IT2	BACH1 intrc	1.3672	0.0016051	0.011055	
ENSG000002	MIR454	microRNA 4	1.648	0.0016101	0.011088	GO:0005615
ENSG000001	C9orf131	chromosom	1.9255	0.0016187	0.011136	
ENSG000002	VPS26BP1	VPS26B pseu	1.4935	0.00163	0.011201	
ENSG000001	BDKRB1	bradykinin r	-1.0435	0.0016301	0.011201	GO:0016020
ENSG000002	PRH1	proline rich	1.3978	0.0016522	0.011336	
ENSG000002	RF00410	-	1.5299	0.0016537	0.011342	
ENSG000001	PPP1R35	protein pho	-1.0725	0.0016621	0.01139	GO:0032515
ENSG000002	AL592310.1	novel pseuc	2.3276	0.0016642	0.011401	
ENSG000002	AC005696.4	novel transc	-2.9986	0.0016842	0.011525	
ENSG000002	LINC00412	long interge	2.7418	0.0016886	0.01155	
ENSG000002	PKP4-AS1	PKP4 antise	1.1336	0.0016943	0.011585	
ENSG000002	RF00019	-	2.5234	0.0016965	0.011595	
ENSG000000	FOXP3	forkhead bc	1.3066	0.0017067	0.011657	GO:0045944
ENSG000002	RF00017	-	2.4329	0.0017213	0.011745	
ENSG000002	GUSBP9	glucuronida	1.2113	0.0017329	0.011814	
ENSG000002	BACH1-AS1	BACH1 antis	1.6648	0.0017331	0.011814	
ENSG000002	DPYD-AS1	DPYD antise	1.8691	0.0017387	0.011847	
ENSG000002	AL049835.1	novel transc	1.5378	0.001743	0.011871	
ENSG000002	AC009927.1	protein tyro	1.5564	0.0017441	0.011875	
Novel.27454	-	-	1.6249	0.0017444	0.011875	
ENSG000002	AC023051.1	novel transc	1.8121	0.001752	0.011923	
ENSG000002	AC003989.2	pseudogene	2.8991	0.0017553	0.011941	
ENSG000002	SMYD3-IT1	SMYD3 intrc	1.4434	0.0017573	0.011952	
ENSG000002	XIAP-AS1	XIAP antiser	2.1945	0.0017582	0.011953	
ENSG000002	AC005838.2	novel transc	1.029	0.0017615	0.011974	
ENSG000002	AC093690.1	uncharacteri	1.4264	0.0017682	0.012011	
ENSG000002	HMGB3P14	high mobilit	2.9337	0.0017709	0.012024	
ENSG000002	RN7SKP9	RNA, 7SK sn	2.9163	0.0017762	0.012058	
ENSG000002	AC138832.1	sterol carrier	2.5139	0.00179	0.012138	
ENSG000002	ZNF425	zinc finger p	-1.348	0.001801	0.01221	GO:0003676
ENSG000002	PCDHGA9	protocadher	1.5412	0.0018093	0.012252	GO:0003723
ENSG000001	ABHD12B	abhydrolase	1.5068	0.0018275	0.012364	GO:0016787
ENSG000002	RF00019	-	1.4225	0.001844	0.012464	
ENSG000002	AC091805.1	ribosomal p	4.1279	0.0018484	0.012477	
ENSG000002	GAPDHP51	glyceraldehy	5.2124	0.0018493	0.012481	
ENSG000002	AC022973.2	novel transc	1.4773	0.0018538	0.012504	
ENSG000002	RPL12P29	ribosomal p	3.0957	0.0018535	0.012504	
ENSG000002	TDGF1P6	teratocarcin	2.7578	0.001874	0.012621	
ENSG000002	AC007256.1	SCY1-like 2	2.0466	0.0019064	0.012811	
ENSG000001	CATSPERD	cation chanı	2.455	0.0019112	0.012833	GO:0016020
ENSG000002	INTS9-AS1	INTS9 antise	1.8511	0.001912	0.012836	
ENSG000002	DIAPH2-AS1	DIAPH2 anti	2.2762	0.0019128	0.012839	
ENSG000002	AC091196.1	novel transc	1.6589	0.0019171	0.01286	
ENSG000002	RN7SL426P	RNA, 7SL, cy	1.5812	0.0019273	0.012915	

ENSG000002	AC025031.4	novel transc	1.6021	0.0019337	0.012953	
ENSG000002	RF00004	-	1.318	0.0019445	0.013022	
ENSG000002	AC005692.3	novel transc	1.9591	0.00195	0.013057	
ENSG000002	AC022306.3	novel transc	5.1363	0.001973	0.013184	
ENSG000002	MIR5739	microRNA 5	3.2691	0.0019953	0.013321	
ENSG000002	BNIP3P27	BCL2 interac	1.4822	0.0019972	0.013331	
ENSG000002	AC099313.1	novel transc	2.2625	0.0020077	0.013388	
ENSG000002	AL391863.1	novel transc	2.5593	0.0020121	0.013412	
ENSG000002	AC006116.5	novel transc	2.566	0.0020264	0.013496	
ENSG000001	Z99496.1	bromodoma	2.5998	0.0020407	0.013578	
ENSG000002	AC011939.1	DnaJ (Hsp40	1.8998	0.0020468	0.013611	
ENSG000002	C9orf147	chromosom	1.4996	0.0020534	0.013642	
ENSG000002	RN7SL2	RNA, 7SL, cy	1.067	0.0020564	0.01366	
ENSG000002	RF00019	-	4.0534	0.0020574	0.013664	
ENSG000002	PCDHA8	protocadher	2.3935	0.0020656	0.013711	GO:0016020
ENSG000002	AL031772.1	novel transc	1.5829	0.0020669	0.013717	
ENSG000002	HMGN2P28	high mobilit	4.0752	0.0020682	0.013721	
ENSG000002	AC002044.2	novel transc	1.6459	0.0020822	0.013798	
ENSG000002	AC015853.1	novel transc	2.9911	0.0020901	0.013837	
ENSG000002	AGGF1P2	angiogenic	1.0608	0.0020939	0.01386	
ENSG000001	FGF13	fibroblast gr	-1.8737	0.0021077	0.013939	GO:0005737
ENSG000002	BX640514.2	novel transc	-2.9753	0.0021216	0.014025	
ENSG000002	AC009137.2	novel transc	2.1137	0.002124	0.014036	
ENSG000002	AC022080.2	ankyrin repe	1.6343	0.0021608	0.014258	
ENSG000002	TNRC18P1	trinucleotide	2.1458	0.0021644	0.014279	
ENSG000002	AC004951.1	novel transc	1.2021	0.0021873	0.014406	
ENSG000002	RNU6-341P	RNA, U6 sm	2.1419	0.0021999	0.014484	
ENSG000001	RF00019	-	1.6883	0.0022386	0.01472	
ENSG000002	AC084782.2	novel transc	3.0931	0.0022491	0.014772	
ENSG000001	RNA5SP319	RNA, 5S ribo	3.2639	0.0022513	0.014784	
ENSG000002	AC103705.1	protein tyro	1.5077	0.0022637	0.014859	
ENSG000002	SNRPGP18	small nuclea	1.8723	0.0022639	0.014859	
ENSG000002	DDX18P5	DEAD-box h	2.2704	0.0022743	0.014924	
ENSG000002	MIR5699	microRNA 5	2.8133	0.0022803	0.014955	
ENSG000002	TDGF1P5	teratocarcin	1.6742	0.0023058	0.015103	
ENSG000002	TTC34	tetratricopep	2.1156	0.0023131	0.015143	
ENSG000002	AC006441.1	novel transc	1.5865	0.0023205	0.015174	
ENSG000002	AL451047.1	novel transc	2.2016	0.0023281	0.015208	
ENSG000002	AC137932.2	uncharacteri	1.8181	0.0023297	0.015215	
ENSG000001	PLN	phospholam	2.7586	0.0023317	0.015226	GO:0048471
ENSG000002	AC098650.1	novel transc	2.8414	0.0023328	0.01523	GO:0008270
ENSG000002	RPS3AP12	ribosomal p	2.2909	0.0023853	0.015536	
Novel.17006	-	-	1.2383	0.0023868	0.015543	
ENSG000002	AL049552.1	novel transc	1.3672	0.0023952	0.015584	
ENSG000002	RNU6-1282I	RNA, U6 sm	4.0165	0.0024036	0.01563	
ENSG000001	RPSAP19	ribosomal p	-1.3142	0.0024066	0.015644	
ENSG000002	AP000753.3	ATPase, H+	2.4593	0.0024066	0.015644	
ENSG000002	RF00019	-	2.9644	0.0024362	0.015804	

ENSG000001	ACKR4	atypical che	1.2205	0.0024536	0.015909	GO:0016020
ENSG000002	Z86062.2	novel transc	3.5047	0.002457	0.015928	
ENSG000001	MAMDC2	MAM doma	2.2358	0.0024597	0.015943	GO:0016020
ENSG000002	AC064799.2	novel transc	2.3327	0.0024714	0.015998	
ENSG000002	MCUR1P1	MCUR1 pseu	1.5498	0.0024714	0.015998	
ENSG000002	MTOR-AS1	MTOR antis	3.0982	0.0024788	0.016041	
ENSG000002	AL450992.3	novel transc	1.4319	0.0024789	0.016041	
ENSG000002	AC091057.7	Serine/threo	2.1608	0.0025047	0.01619	
ENSG000002	H2AFZP4	H2A histone	1.9277	0.0025069	0.016202	
ENSG000001	HIST2H2AC	histone clus	1.5677	0.0025103	0.01622	GO:0005634
ENSG000002	AC099811.5	novel transc	1.4401	0.002523	0.01629	
ENSG000002	AC009303.2	novel transc	1.5686	0.0025433	0.016407	
ENSG000002	LINC02340	long interge	2.2436	0.0025519	0.016457	
ENSG000000	IL12RB2	interleukin 1	1.2598	0.0025595	0.0165	GO:0016020
ENSG000002	AC074117.2	ribosomal p	5.135	0.0025705	0.016561	
ENSG000002	AC010168.2	novel transc	1.3003	0.0025865	0.01665	
ENSG000001	PCSK1	proprotein c	1.4135	0.0025985	0.016711	GO:0005615
ENSG000001	RNU6-859P	RNA, U6 sm	3.4938	0.0026044	0.016744	
ENSG000001	PHLDA2	pleckstrin ho	-1.1741	0.0026055	0.016748	GO:0005737
ENSG000002	AC010638.1	novel transc	3.1945	0.0026074	0.016757	
ENSG000002	AC114763.1	novel transc	1.1821	0.0026153	0.016793	
ENSG000002	AC005842.1	uncharacteri	2.0251	0.0026442	0.016951	
ENSG000002	SMIM5	small integra	1.2411	0.0026753	0.017126	GO:0016020
ENSG000002	AL391095.1	novel transc	2.0827	0.0026924	0.017209	
Novel.16998	-	-	2.1086	0.0026965	0.017231	
ENSG000002	RPL12P44	ribosomal p	2.9864	0.0026973	0.017233	
ENSG000002	SLC10A5	solute carrie	1.9777	0.002722	0.017369	GO:0016020
ENSG000001	RAB24	RAB24, mem	-1.0587	0.0027374	0.017442	GO:0005737
ENSG000002	AC067940.1	PHD finger p	2.5841	0.0027511	0.017517	
ENSG000002	AL445187.1	vacuolar pro	1.7028	0.00276	0.017565	
ENSG000002	MIR1205	microRNA 1	1.346	0.0027623	0.017576	
ENSG000002	UBE2V1P13	ubiquitin co	3.5155	0.0027697	0.017617	
ENSG000002	TXNP6	thioredoxin	1.8323	0.0027812	0.017681	
ENSG000002	AC022336.3	TEC	2.1557	0.0027879	0.017714	
ENSG000002	KLF7-IT1	KLF7 introni	1.4355	0.0027955	0.017759	
ENSG000002	RNU7-79P	RNA, U7 sm	3.0287	0.002804	0.017803	
ENSG000002	RNU6-509P	RNA, U6 sm	5.0871	0.0028141	0.017857	
ENSG000002	RF00019	-	1.8768	0.0028167	0.017868	
ENSG000001	SSTR1	somatostati	-1.8948	0.0028189	0.017879	GO:0016020
ENSG000002	AL359918.2	novel transc	2.4061	0.0028459	0.018031	
Novel.17005	-	-	1.4322	0.0028526	0.018064	
ENSG000001	FAM87B	family with s	2.4559	0.0028553	0.018075	
ENSG000002	RNU6-83P	RNA, U6 sm	5.0317	0.002859	0.018089	
ENSG000002	OR4F14P	olfactory rec	5.0317	0.002859	0.018089	
ENSG000002	BANF1P1	barrier to au	1.2132	0.0028633	0.018112	
ENSG000001	CCDC54	coiled-coil c	2.7246	0.0028848	0.018229	
ENSG000002	SEPT7P6	septin 7 pse	1.636	0.0028859	0.018233	
ENSG000002	AC025031.5	TEC	1.8213	0.0028909	0.018261	

ENSG000002	AC055733.1	cysteine and	2.5317	0.0028996	0.01831	
ENSG000002	AC011676.3	novel transc	2.061	0.0029197	0.018414	
ENSG000002	FTLP6	ferritin light	1.918	0.0029557	0.018595	
Novel.27456	-	-	3.1464	0.0029637	0.018639	
ENSG000001	FOXP2	forkhead bo	1.145	0.0029716	0.018686	GO:0005634
ENSG000002	AC068491.2	novel transc	2.0813	0.0029727	0.018689	
ENSG000002	RNU6-522P	RNA, U6 sm	4.5417	0.0029762	0.018708	
ENSG000002	AL807757.1	novel transc	2.3934	0.0029992	0.018846	
ENSG000002	MIR135A1	microRNA 1	2.9377	0.0030087	0.018885	GO:1903231
ENSG000002	XKR9	XK related 9	2.0413	0.0030155	0.018921	GO:0016020
ENSG000002	AC020910.3	small nuclea	3.4835	0.0030272	0.018986	
ENSG000001	FAM217A	family with s	1.7405	0.0030343	0.01902	
ENSG000002	OR2L9P	olfactory rec	3.1571	0.0030492	0.019103	
ENSG000002	AC008871.1	novel transc	2.4854	0.0030699	0.019216	
ENSG000002	STX16-NPEF	STX16-NPEF	1.4398	0.003081	0.019275	GO:0006886
ENSG000002	AC023830.1	novel transc	1.4986	0.0030984	0.019374	
ENSG000002	AP000897.2	novel transc	2.2554	0.0031246	0.019517	
ENSG000002	HIGD1AP17	HIG1 hypoxi	3.9801	0.0031374	0.019586	
ENSG000001	CAMK2N1	calcium/calr	-1.1749	0.003151	0.019665	GO:0016020
ENSG000002	FAM185BP	family with s	1.0253	0.0031636	0.019713	
ENSG000002	AC010240.2	eukaryotic t	1.2228	0.0031856	0.019833	
ENSG000002	AL390774.2	novel transc	2.2812	0.0031915	0.019852	
ENSG000001	NKPD1	NTPase KAP	1.4816	0.0032108	0.019955	GO:0016020
ENSG000002	RN7SL735P	RNA, 7SL, cy	3.0124	0.0032126	0.019962	
ENSG000002	FP671120.4	novel transc	1.5945	0.0032738	0.020305	
ENSG000002	FAM96AP2	family with s	2.6429	0.003289	0.020374	
ENSG000002	G2E3-AS1	G2E3 antiser	1.5272	0.0032917	0.020383	
ENSG000002	AL162724.1	novel transc	1.566	0.0032943	0.020389	
ENSG000002	RNU4-87P	RNA, U4 sm	4.534	0.0033151	0.020496	
ENSG000001	SH3RF2	SH3 domain	1.0988	0.0033163	0.020501	GO:0005634
ENSG000001	GHRL	ghrelin and	1.1428	0.0033241	0.020535	GO:0010469
ENSG000002	RPS7P15	ribosomal p	2.7634	0.0033304	0.02057	
ENSG000001	KLRB1	killer cell lec	1.1018	0.0033399	0.020618	GO:0016020
ENSG000002	AC118555.1	novel transc	1.958	0.0033534	0.020687	
ENSG000002	MTND5P11	MT-ND5 pse	1.2432	0.0033586	0.020716	
ENSG000002	AC007114.2	novel transc	1.2668	0.0033806	0.020841	
ENSG000001	CACNG6	calcium volt	-1.5534	0.0033847	0.020859	GO:0016020
ENSG000002	AL591721.1	novel transc	1.7494	0.0033996	0.020936	
ENSG000002	RF00019	-	3.4397	0.0033991	0.020936	
ENSG000002	MIR3144	microRNA 3	2.8074	0.0034006	0.020938	
ENSG000002	AC011239.2	TEC	2.2498	0.0034033	0.020951	
ENSG000002	RN7SL370P	RNA, 7SL, cy	3.9205	0.0034363	0.02114	
ENSG000000	SPTB	spectrin bet	1.3995	0.0034416	0.021169	GO:0005737
ENSG000002	AC090286.2	RAN bindin	1.4609	0.0034439	0.02118	
ENSG000000	PER3	period circa	1.0366	0.0034546	0.02122	GO:0032922
ENSG000002	RN7SL733P	RNA, 7SL, cy	4.5175	0.0034537	0.02122	
ENSG000002	AC006270.1	novel transc	2.6324	0.0034668	0.021287	
ENSG000002	RF00019	-	2.8004	0.0034822	0.021375	

ENSG000002	RN7SKP158	RNA, 7SK sn	2.6807	0.0034923	0.021422	
Novel.11445	-	-	2.6816	0.0034924	0.021422	
ENSG000002	HSPE1P13	heat shock p	1.912	0.0035079	0.021514	
ENSG000002	AL356776.1	nucleoporin	2.085	0.0035143	0.021546	
ENSG000002	AC048380.2	TEC	1.1018	0.0035399	0.021685	
ENSG000002	AC104982.1	novel transc	1.2878	0.003552	0.021751	
ENSG000001	C6orf141	chromosom	1.5023	0.003554	0.021759	
ENSG000002	YPEL5P2	YPEL5 pseu	1.6167	0.0035566	0.021768	
ENSG000002	RPSAP36	ribosomal p	2.2337	0.0035662	0.021816	
ENSG000002	SPTLC1P1	serine palmi	2.2358	0.0035674	0.021819	
ENSG000002	RNA5SP78	RNA, 5S ribo	1.8482	0.0035699	0.021831	
ENSG000002	AL354726.1	novel transc	1.602	0.0035742	0.021849	
ENSG000002	ETF1P1	eukaryotic t	2.5975	0.0035759	0.021856	
ENSG000002	AC011676.1	novel transc	2.1334	0.0035777	0.02186	
ENSG000002	AC027544.2	novel transc	1.8766	0.0035775	0.02186	
ENSG000002	AL589666.2	TEC	2.9656	0.0035799	0.02187	
ENSG000002	AC007684.1	novel transc	2.4173	0.0035931	0.021947	
ENSG000002	ARAP1-AS2	ARAP1 antis	1.3787	0.003598	0.021969	
ENSG000002	AL136379.1	novel transc	2.5996	0.003603	0.021996	
ENSG000002	RNY1P13	RNA, Ro-ass	1.8548	0.0036187	0.02208	
ENSG000002	AC131212.2	novel transc	1.6018	0.0036267	0.022121	
ENSG000001	CHRM5	cholinergic r	3.4038	0.0036318	0.022147	GO:0016020
ENSG000002	AL158801.3	novel transc	1.5973	0.0036329	0.022148	
ENSG000001	HSPE1P11	heat shock p	2.3638	0.0036367	0.022163	
ENSG000001	NR4A2	nuclear rece	-1.1249	0.0036374	0.022164	GO:0005634
ENSG000001	DMKN	dermokine	-4.2106	0.0036491	0.022224	GO:0005515
ENSG000002	AL513365.2	uncharacteri	1.9737	0.003661	0.022293	
ENSG000002	AC022079.2	novel transc	1.601	0.0036639	0.022303	
ENSG000002	AL365436.2	novel transc	1.8948	0.0036662	0.022313	
ENSG000002	PCDHA7	protocadher	1.7597	0.003668	0.02232	GO:0016020
ENSG000002	RPL36AP41	ribosomal p	1.7881	0.0036689	0.022322	
ENSG000002	RPL37P18	ribosomal p	2.2684	0.0036716	0.022327	
ENSG000002	BNIP3P16	BCL2 interac	1.2427	0.0036717	0.022327	
ENSG000002	AL139174.1	methylmalo	3.1374	0.0036849	0.022393	
ENSG000002	ENPP7P4	ectonucleoti	1.2136	0.0037003	0.022475	
ENSG000002	FAM53B-AS	FAM53B ant	2.7438	0.0036999	0.022475	
ENSG000002	AL357054.1	novel serine	1.7043	0.0037309	0.022634	
ENSG000002	RNU4ATAC1	RNA, U4atac	1.8497	0.0037515	0.022739	
ENSG000002	MAGI2-AS1	MAGI2 antis	3.1316	0.0037778	0.022868	
ENSG000002	BNIP3P24	BCL2 interac	1.5613	0.0037787	0.022869	
ENSG000002	RF00554	-	3.9097	0.0037845	0.022889	
ENSG000002	AC211433.1	uncharacteri	1.1171	0.003784	0.022889	
ENSG000002	AC022540.1	uncharacteri	1.239	0.003792	0.022931	
Novel.19108	-	-	1.2867	0.00386	0.023318	
ENSG000002	AC092798.1	ribosomal p	1.9567	0.0038685	0.023354	
ENSG000002	RPL23AP35	ribosomal p	4.985	0.0039193	0.023624	
ENSG000002	RNU6-198P	RNA, U6 sm	3.4027	0.0039395	0.023722	
ENSG000001	RAB40A	RAB40A, me	1.0575	0.0039622	0.023839	GO:0005622

ENSG000002	AL121900.1	uncharacter	1.7474	0.0039652	0.023846	
ENSG000002	RPL10P12	ribosomal p	1.4841	0.0039718	0.023878	
ENSG000001	ADGRG2	adhesion G	1.4811	0.003975	0.023888	GO:0016020
ENSG000002	ARL4AP5	ADP ribosyl	1.7867	0.0040157	0.024104	
ENSG000002	AP002453.1	adenosine c	2.5826	0.0040253	0.024138	
ENSG000002	AL592437.2	DEP domain	1.672	0.0040526	0.02426	
ENSG000002	RSL24D1P8	ribosomal L	1.6821	0.0040518	0.02426	
ENSG000002	AC097716.1	novel transc	4.9185	0.0040543	0.024267	
ENSG000002	ANKRD33B-	ANKRD33B	1.8327	0.0040672	0.02434	
ENSG000001	LGALS2	galectin 2	2.9653	0.0040704	0.024351	GO:0030246
ENSG000002	AL590723.1	novel transc	1.9213	0.0040759	0.02438	
ENSG000002	RPS12P16	ribosomal p	1.4518	0.0040997	0.024514	
ENSG000002	RAB5CP1	RAB5C, men	1.7402	0.004111	0.024573	
ENSG000002	ZMYM4-AS	ZMYM4 anti	2.0344	0.004131	0.024681	
ENSG000002	RN7SL57P	RNA, 7SL, cy	3.8882	0.0041408	0.024727	
ENSG000002	CBX5P1	chromobox	1.7242	0.0041446	0.024745	
ENSG000002	AC067931.1	TEC	1.2857	0.0041671	0.024871	
ENSG000002	CDC42P1	cell division	2.9156	0.0041712	0.024891	
ENSG000001	SPDYE16	speedy/RIN	1.6581	0.004173	0.024898	GO:0019901
ENSG000002	AC027682.4	novel transc	2.092	0.0041873	0.024975	
ENSG000000	MATK	megakaryoc	-2.3915	0.0042086	0.025077	GO:0005737
ENSG000002	AC011944.2	tec	2.0698	0.0042107	0.025086	
Novel.17771	-	-	1.7111	0.0042256	0.02517	
ENSG000001	RNASEK-C1	RNASEK-C1	4.9669	0.0042341	0.025212	GO:0016020
ENSG000002	AC073863.1	novel transc	4.581	0.0042602	0.025343	
ENSG000002	AL390957.1	novel transc	2.9366	0.0042749	0.025414	
ENSG000001	BCL2L14	BCL2 like 14	1.629	0.0042992	0.025523	GO:0005737
ENSG000001	KCTD4	potassium c	2.2567	0.0043448	0.02577	GO:0051260
ENSG000002	RNU6-1003I	RNA, U6 sm	1.9375	0.0043551	0.025826	
ENSG000002	AL445523.1	karyopherin	1.9092	0.0043633	0.025866	
ENSG000002	AC091132.5	novel transc	1.5319	0.004392	0.026019	
ENSG000001	NUTM2B	NUT family	1.3054	0.0044015	0.026071	
ENSG000002	AC012464.2	novel transc	2.3278	0.0044084	0.026103	
ENSG000002	AC005520.3	novel transc	1.3995	0.0044108	0.026113	
ENSG000002	AC091769.2	novel transc	1.9738	0.0044446	0.026274	
ENSG000002	YRDCP2	yrdC N(6)-tl	2.2709	0.0044603	0.026358	
ENSG000002	AL080317.2	novel transc	1.1022	0.0044647	0.026376	
ENSG000002	MTND6P5	MT-ND6 pse	3.3403	0.0045141	0.026634	
ENSG000002	AC073530.2	TEC	4.4184	0.0045225	0.026668	
ENSG000002	AL353754.1	mitochondr	2.121	0.0045248	0.026673	
ENSG000001	IRX3	iroquois hor	-4.5031	0.0045357	0.026733	GO:0005634
ENSG000002	HNRNPRP1	heterogene	1.3819	0.0045428	0.026766	
ENSG000002	OR2L6P	olfactory re	3.0473	0.0045488	0.026797	
ENSG000001	CASP1	caspase 1	-1.5659	0.0045658	0.026888	GO:0016787
ENSG000001	TMPRSS5	transmembr	1.2965	0.0045736	0.026925	GO:0016020
ENSG000002	AC022613.3	novel transc	2.039	0.0045772	0.026938	
ENSG000002	AC022819.1	novel transc	1.8144	0.0045901	0.026996	
ENSG000002	AC133106.1	novel transc	1.9801	0.0045995	0.027038	

ENSG000002	AC004217.2	DnaJ (Hsp40	2.7083	0.0046191	0.02714	
ENSG000001	SULF2	sulfatase 2	2.3252	0.0046253	0.027163	GO:0040037
ENSG000002	THSD4-AS1	THSD4 antis	2.105	0.0046505	0.027293	
ENSG000002	RNU4-31P	RNA, U4 sm	3.1243	0.0046639	0.027354	
ENSG000002	AC024267.5	novel transc	1.7816	0.004667	0.027366	
ENSG000002	RPL21P136	ribosomal p	1.9823	0.0046715	0.027384	
ENSG000002	AC090644.1	-	4.9189	0.0047074	0.027563	
ENSG000002	AP003086.3	novel transc	2.1536	0.0047102	0.027571	
ENSG000002	PCDHA3	protocadher	1.5268	0.0047457	0.027751	GO:0016020
ENSG000002	AC092332.1	novel transc	3.0968	0.0047544	0.027793	
ENSG000002	AC073592.6	novel transc	1.7894	0.0047781	0.027918	
ENSG000002	PKIA-AS1	PKIA antiser	2.0931	0.0047816	0.027929	
ENSG000002	KIZ-AS1	KIZ antisens	1.321	0.0048	0.028018	
ENSG000002	RF01210	-	3.4015	0.0048296	0.028178	
ENSG000002	RNU6-1305I	RNA, U6 sm	2.8972	0.0048671	0.028387	
ENSG000002	RF00598	-	2.1989	0.0048699	0.028399	
ENSG000002	AL035693.1	novel transc	2.2608	0.0048738	0.028417	
ENSG000002	AL390318.1	TEC	2.7509	0.0048948	0.028535	
ENSG000002	PDHA1P1	pyruvate de	1.5315	0.0049282	0.028711	
ENSG000002	AC068790.2	novel transc	1.1318	0.0049382	0.028765	
ENSG000002	AL118556.2	zinc finger p	2.3087	0.0049425	0.028785	
ENSG000002	AL138963.1	novel transc	1.4794	0.0049567	0.028854	
ENSG000002	RNU6-1112I	RNA, U6 sm	3.4302	0.0049631	0.028881	
ENSG000001	RNF113B	ring finger p	2.3458	0.0049674	0.028902	GO:0046872
ENSG000002	AC245052.2	leukocyte in	4.4529	0.0049951	0.029039	
ENSG000002	MTND2P40	MT-ND2 pse	1.1533	0.0050075	0.029097	
ENSG000002	AC083875.1	ribosomal p	3.5947	0.005022	0.029172	
ENSG000002	AC009754.1	novel transc	3.5081	0.0050234	0.029175	
ENSG000002	AP001011.1	novel transc	3.1322	0.0050286	0.029198	
ENSG000002	AC021106.2	proteasome	2.2453	0.0050313	0.029202	
ENSG000001	CLEC4F	C-type lectir	-4.4981	0.0050367	0.029229	GO:0016020
ENSG000002	DNM1P47	dynamin 1 p	1.377	0.0050688	0.029386	
ENSG000002	NAIP	NLR family a	1.1308	0.0050792	0.029442	GO:0005737
ENSG000002	AC091132.2	novel transc	1.186	0.0050828	0.029458	
ENSG000002	MIR640	microRNA 6	2.0787	0.0050959	0.029526	
ENSG000001	LRRC9	leucine rich	3.0319	0.005121	0.029661	
ENSG000001	TRIM72	tripartite mo	4.4183	0.0051235	0.02967	GO:0016020
ENSG000002	FCF1P5	FCF1 pseudo	2.6522	0.0051259	0.029675	
ENSG000002	RPL23AP17	ribosomal p	1.5963	0.0051362	0.029725	
ENSG000002	AC099336.1	FK506 bindi	4.4791	0.0051506	0.029803	
ENSG000002	AP005899.1	novel transc	1.477	0.0051558	0.029823	
ENSG000002	RF00019	-	2.1546	0.005158	0.029832	
ENSG000001	KLHL35	kelch like fa	1.5821	0.0051652	0.029868	GO:0005515
ENSG000001	GPR34	G protein-co	2.3468	0.0051867	0.029973	GO:0016020
ENSG000002	AL031281.1	M-phase ph	1.1993	0.005212	0.030115	
ENSG000002	RPL19P14	ribosomal p	3.3877	0.0052312	0.030215	
ENSG000002	RNA5SP282	RNA, 5S ribo	2.5942	0.0052521	0.030317	
ENSG000001	GNG3	G protein su	1.1772	0.0052799	0.030463	GO:0016020

ENSG000002	FO393419.2	novel transc	1.8017	0.0052832	0.030477	
ENSG000002	AC006449.1	novel transc	4.3834	0.0052964	0.030528	
ENSG000002	OR7E126P	olfactory rec	1.5581	0.005314	0.03061	
ENSG000002	SCARNA3	small Cajal b	2.3406	0.0053231	0.030647	
ENSG000001	UPK1B	uroplakin 1E	2.2442	0.0053286	0.030675	GO:0016020
ENSG000002	AL157932.1	novel transc	4.3655	0.0053333	0.030696	
ENSG000002	ATP8A2P2	ATPase pho	4.3747	0.0053397	0.030728	
ENSG000002	RF00139	-	1.733	0.0053485	0.03077	
ENSG000001	MIRLET7BH(	MIRLET7B h	1.1777	0.0053607	0.030821	
ENSG000002	ARHGDIG	Rho GDP dis	-1.1147	0.0053788	0.030904	GO:0005737
ENSG000002	AC093422.2	novel transc	3.3209	0.0053919	0.030972	
ENSG000002	KRT8P33	keratin 8 pse	1.0292	0.0053932	0.030972	
ENSG000002	AC118553.2	novel protei	1.41	0.0054356	0.03117	GO:0016020
ENSG000002	AL445489.1	novel transc	2.4346	0.005437	0.03117	
ENSG000002	B3GALNT1P	beta-1,3-N-	1.1764	0.0054483	0.031219	
ENSG000002	PDCD4-AS1	PDCD4 antis	-1.8497	0.0055018	0.031469	
ENSG000002	AC009242.1	novel transc	1.2657	0.0055481	0.031719	
ENSG000002	AL353583.1	TEC	2.5145	0.0055541	0.031728	
ENSG000002	AL162724.2	novel transc	1.6522	0.0055784	0.031862	
ENSG000002	AC046158.1	novel transc	2.4971	0.0056079	0.031989	
ENSG000002	AL162431.4	cell division	3.8166	0.0056321	0.032112	
ENSG000002	AC087286.1	novel transc	2.7984	0.0056621	0.032273	
ENSG000002	AC004134.1	transmembr	3.3405	0.0056698	0.032296	
ENSG000001	RILP	Rab interact	-1.234	0.0056743	0.032317	GO:0046983
ENSG000002	SERBP1P6	SERPINE1 m	1.4807	0.0056753	0.032317	
ENSG000002	BNIP3P38	BCL2 interac	1.8811	0.0056994	0.032449	
ENSG000002	HSP90AB2P	heat shock p	2.3652	0.0057075	0.032485	
ENSG000002	AC245060.2	novel transc	1.5706	0.0057719	0.032815	
ENSG000002	AL359762.3	novel transc	1.4684	0.0058491	0.033212	
ENSG000002	AC100774.1	novel transc	2.3644	0.0058529	0.033223	
ENSG000002	AC024619.3	novel transc	2.8362	0.0058558	0.033224	
ENSG000002	RF00019	-	2.1965	0.0058899	0.03339	
ENSG000002	AL449214.1	-	1.9613	0.0059165	0.033531	
ENSG000002	AC114763.2	novel transc	1.3889	0.0059178	0.033532	
ENSG000002	AC036103.1	novel transc	1.9645	0.0059474	0.033679	
ENSG000002	MTND6P4	MT-ND6 pse	2.2965	0.0059521	0.03369	
ENSG000002	EIF4E2P1	eukaryotic t	4.822	0.005958	0.033713	
ENSG000002	AC016542.1	novel transc	1.8109	0.005968	0.033753	
ENSG000002	RF00019	-	1.3753	0.0059671	0.033753	
ENSG000002	RPL31P60	ribosomal p	4.8938	0.0059763	0.033784	
ENSG000002	BMP2KL	BMP2 induc	3.7685	0.0060011	0.033908	
ENSG000002	RPL9P30	ribosomal p	1.6533	0.006026	0.034016	
ENSG000002	KLF3P1	Kruppel like	1.901	0.0060472	0.034131	
ENSG000002	AL133368.1	ribosomal p	1.7833	0.006053	0.034158	
ENSG000002	AL451062.1	uncharacteri	2.0052	0.006083	0.034296	
ENSG000002	AC105402.3	novel transc	1.3456	0.0061082	0.034427	
ENSG000002	AL022323.3	novel transc	1.7096	0.0061145	0.034457	
ENSG000002	AC055811.1	novel transc	1.4026	0.0061162	0.034461	

ENSG000002	AC006116.2	NADH dehy	2.6066	0.006135	0.034528	
ENSG000002	RF00601	-	1.5285	0.0061465	0.034588	
ENSG000002	AP003086.1	uncharacteri	1.7855	0.006158	0.034642	
ENSG000002	RNU1-91P	RNA, U1 sm	2.8153	0.0061718	0.034714	
ENSG000002	KLF4P1	Kruppel like	4.3911	0.0061815	0.034763	
ENSG000002	MRC1	mannose re	4.8089	0.0061825	0.034763	GO:0016020
ENSG000001	ACRV1	acrosomal v	1.6042	0.0061908	0.034804	GO:0031410
ENSG000002	AL121890.4	novel transc	1.7838	0.0061957	0.034821	
ENSG000002	AC114878.2	CTAGE fami	2.38	0.0061979	0.034828	
ENSG000002	PCDHA13	protocadher	2.778	0.0062022	0.034841	GO:0016020
ENSG000002	AL513523.2	immunoglol	2.2246	0.0062118	0.034886	
ENSG000002	AC007790.1	ribosomal p	1.841	0.0062604	0.03508	
ENSG000002	AL162386.2	novel transc	1.5618	0.0062794	0.035174	
ENSG000002	AC027644.1	pseudogene	1.3058	0.0062864	0.035193	
ENSG000002	ADH5P3	alcohol deh	1.4641	0.0062913	0.035215	
ENSG000002	HSPA8P19	heat shock p	1.5307	0.0063279	0.035368	
ENSG000001	RN7SKP95	RNA, 7SK sn	4.8427	0.0063524	0.035479	
ENSG000002	RN7SKP271	RNA, 7SK sn	2.0625	0.0063918	0.035665	
ENSG000002	AL162390.1	novel transc	1.8323	0.006407	0.035734	
ENSG000001	GPR171	G protein-co	2.0804	0.0064309	0.035856	GO:0016020
ENSG000002	AL391863.2	novel transc	2.7557	0.0065556	0.036483	
ENSG000001	C1QL1	complement	-1.0618	0.0065725	0.036566	GO:0005515
ENSG000002	RF00019	-	4.9165	0.0065821	0.036608	
ENSG000002	NDUFA8P1	NADH:ubiqu	2.9824	0.0066022	0.036709	
ENSG000002	AL122013.1	ribosomal p	1.8489	0.0066024	0.036709	
ENSG000002	MTCYBP3	MT-CYB pse	2.3473	0.0066068	0.036722	
ENSG000002	AC004672.1	novel transc	1.3897	0.0066123	0.036747	
ENSG000002	RN7SL5P	RNA, 7SL, cy	2.0077	0.0066167	0.036749	
ENSG000002	AC026894.1	novel transc	1.967	0.0066245	0.036781	
ENSG000002	AC006116.9	novel transc	1.8842	0.0066459	0.036888	
ENSG000001	FGL2	fibrinogen l	1.7662	0.0066473	0.03689	GO:0005576
ENSG000001	SLC3A1	solute carrie	2.0964	0.0066648	0.03697	GO:0016020
ENSG000002	AC073367.1	novel transc	1.3627	0.006673	0.036993	
ENSG000001	CXCR6	C-X-C motif	1.4381	0.0066806	0.037028	GO:0005622
ENSG000002	RF00019	-	1.9898	0.0066933	0.037082	
ENSG000002	AC010531.4	uncharacteri	2.1527	0.0067051	0.037136	
ENSG000002	AC120349.3	TEC	2.4456	0.0067192	0.037203	
ENSG000002	HIST1H4D	histone clus	1.9525	0.0067233	0.03722	GO:0003723
ENSG000002	HIST1H4A	histone clus	1.9652	0.0067299	0.037245	GO:0003723
ENSG000002	AL513412.1	novel transc	2.0898	0.0067386	0.037287	
ENSG000002	FSBP	fibrinogen s	2.9582	0.0067553	0.037356	GO:0005634
ENSG000002	AC002310.6	novel transc	-1.1933	0.00677	0.03742	
Novel.1414	-	-	1.1495	0.006782	0.037475	
ENSG000002	AL139156.1	transformer	2.062	0.0067921	0.037525	
ENSG000002	ADPGK-AS1	ADPGK antis	1.3913	0.0068062	0.03758	
ENSG000002	RPSAP6	ribosomal p	2.357	0.0068107	0.037599	
ENSG000002	BRWD1-IT1	BRWD1 intro	2.871	0.0068239	0.037666	
ENSG000002	AL359504.1	thioredoxin	1.8591	0.0068324	0.037684	

ENSG000002	AC116158.1	novel transc	1.5155	0.0068318	0.037684	
ENSG000002	NDUFB4P3	NADH:ubiqu	4.3019	0.0068369	0.037691	
ENSG000002	AC124856.1	ribosomal p	1.2845	0.0068713	0.037869	
ENSG000002	RF02271	-	2.9953	0.0069049	0.038031	
ENSG000001	SMCO3	single-pass	3.7449	0.0069074	0.038039	GO:0016020
ENSG000002	OR52I1	olfactory rec	4.3115	0.0069152	0.038076	GO:0016020
ENSG000001	TENT5C	terminal nuc	-2.9723	0.0069197	0.038095	GO:0003723
ENSG000002	RF00019	-	1.9968	0.0069207	0.038095	
ENSG000002	AL450263.2	density-regu	4.7691	0.0069346	0.038148	
ENSG000002	RPS24P16	ribosomal p	1.8725	0.0069424	0.038179	
ENSG000002	AC090236.2	novel transc	2.3115	0.0069466	0.038196	
ENSG000002	AL445248.1	novel transc	1.1737	0.006948	0.038198	
ENSG000002	AL109628.1	ribosomal p	3.2758	0.0069553	0.038226	
ENSG000002	RN7SL65P	RNA, 7SL, cy	3.7411	0.0069607	0.03825	
ENSG000002	PCDHGA5	protocadher	2.3379	0.0069724	0.038303	GO:0016020
ENSG000002	BIRC6-AS1	BIRC6 antise	2.0173	0.0069812	0.038345	
ENSG000002	AC145207.9	TEC	1.887	0.006997	0.03842	
ENSG000002	AC011405.1	novel transc	1.1894	0.007008	0.038469	
ENSG000002	RNU6-1297I	RNA, U6 sm	4.3808	0.007018	0.038512	
ENSG000002	KIF28P	kinesin fami	1.0858	0.0070341	0.038588	
ENSG000002	AC139792.1	novel transc	2.5042	0.0070452	0.038643	
ENSG000002	PLCE1-AS2	PLCE1 antise	2.9703	0.0070507	0.038668	
ENSG000002	AL357153.2	novel transc	3.3493	0.007066	0.038734	
ENSG000002	AC016542.2	TEC	1.6402	0.0070812	0.038806	
ENSG000002	AL135999.2	TEC	2.2448	0.0071316	0.039052	
ENSG000002	SAMM50P1	SAMM50 so	2.2196	0.0071443	0.039073	
ENSG000001	FOLR3	folate recep	-1.7987	0.0071462	0.039078	GO:0005542
ENSG000002	RN7SL623P	RNA, 7SL, cy	4.7961	0.0071771	0.039223	
ENSG000002	RPL7AP11	ribosomal p	1.4252	0.0071915	0.039295	
ENSG000002	AL354696.2	uncharacteri	1.455	0.0071967	0.039318	
ENSG000002	AC023051.2	Enoyl-CoA I	4.7559	0.0072205	0.039424	
ENSG000001	OR4F15	olfactory rec	2.6142	0.0072333	0.03947	GO:0016020
ENSG000002	AC092368.3	novel transc	-1.4084	0.0072691	0.039653	
ENSG000002	AC007000.2	pseudogene	1.5837	0.0072917	0.039758	
ENSG000002	BTBD9-AS1	BTBD9 antis	1.9508	0.0072954	0.039772	
ENSG000002	AC092910.2	riboflavin ki	2.7609	0.0073194	0.039885	
ENSG000002	RN7SL254P	RNA, 7SL, cy	3.3234	0.007338	0.039968	
ENSG000001	HTR5BP	5-hydroxytr	1.6914	0.007349	0.040022	
ENSG000002	SMARCE1P1	SWI/SNF rel	2.5752	0.0073577	0.040062	
ENSG000002	AL590762.1	pseudogene	1.1834	0.0073672	0.040097	
ENSG000002	CYS1	cystin 1	-1.2602	0.0073762	0.040139	GO:0005737
Novel.10233	-	-	2.5023	0.0074213	0.04036	
ENSG000001	TAS2R3	taste 2 recep	1.8297	0.0074561	0.040525	GO:0016020
ENSG000002	MRPL50P2	mitochondri	4.3131	0.00746	0.04054	
ENSG000001	DIRAS3	DIRAS family	-1.6747	0.0074768	0.040599	GO:0005622
ENSG000002	SPDYE21P	speedy/RING	1.0583	0.0075063	0.040718	
ENSG000002	C17orf78	chromosom	1.2236	0.0075219	0.040787	GO:0016020
ENSG000002	AC087683.2	novel transc	2.3216	0.0075226	0.040787	

ENSG000002	AD000813.1	TEC	1.3273	0.0075555	0.040953	
ENSG000002	DCTN1-AS1	DCTN1 antis	1.8189	0.0075602	0.040973	
ENSG000002	PTGES3P2	prostagland	2.3905	0.0075946	0.041147	
ENSG000001	SDS	serine dehy	1.5262	0.0076183	0.04125	GO:0005737
ENSG000002	RF00019	-	2.5452	0.0076261	0.04128	
ENSG000002	LINC00370	long interge	2.9658	0.007644	0.041351	
ENSG000002	AC008937.2	novel transc	2.6351	0.0076594	0.041416	
ENSG000002	AC104024.2	novel transc	-4.3469	0.0076637	0.041433	
ENSG000002	HIST1H4F	histone clus	1.5445	0.0076656	0.041437	GO:0003723
ENSG000002	AC104115.2	zinc finger p	1.0842	0.0076779	0.041479	
ENSG000001	DOK7	docking pro	2.249	0.0076826	0.041498	GO:0016020
ENSG000002	AC025171.3	novel transc	1.0649	0.0076878	0.041513	
ENSG000002	HLCS-IT1	HLCS intron	1.8677	0.0076912	0.041526	
ENSG000002	AC124283.3	novel transc	1.6114	0.0076964	0.041547	
ENSG000002	AC006539.2	BCL2/adenc	1.302	0.0077181	0.041639	
ENSG000002	AC017071.1	novel transc	1.7159	0.007722	0.041654	
ENSG000002	AC025186.1	ribosomal p	1.0437	0.0077573	0.041813	
ENSG000002	Z98749.1	novel transc	1.7859	0.0077639	0.041842	
ENSG000002	AC002044.3	novel transc	1.6403	0.0077858	0.041954	
ENSG000002	NCOA7-AS1	NCOA7 anti	4.2741	0.0078064	0.042046	
ENSG000002	AC011411.1	transient rec	1.4678	0.0078338	0.042169	
ENSG000002	PPIAP6	peptidylpro	2.6054	0.0078447	0.042208	
ENSG000002	MAGOH3P	mago homc	4.2451	0.0078582	0.042268	
ENSG000002	AC022960.2	novel transc	2.6056	0.0078659	0.042303	
ENSG000000	LAMC2	laminin sub	-1.028	0.0078851	0.0424	GO:0008284
ENSG000002	AC079035.1	novel transc	1.566	0.0078902	0.042415	
ENSG000002	PIGFP2	phosphatidy	2.7686	0.0079314	0.042604	
ENSG000002	AC004893.1	ring finger p	1.6532	0.0079336	0.042609	
ENSG000002	AL451063.1	tRNA nucle	3.6966	0.0079362	0.042617	
ENSG000002	AC090543.1	ribosomal p	1.7037	0.0079376	0.042618	
ENSG000002	RN7SL239P	RNA, 7SL, cy	1.6174	0.007947	0.042656	
ENSG000002	RNU6-780P	RNA, U6 sm	1.475	0.0080813	0.043312	
ENSG000001	TRIML1	tripartite mc	1.5017	0.0081209	0.043511	GO:0046872
ENSG000001	COL4A3	collagen typ	1.1724	0.0081222	0.043512	GO:0043231
ENSG000002	RNU6-141P	RNA, U6 sm	1.6976	0.0081337	0.04356	
ENSG000002	AC103739.2	novel transc	1.6114	0.0081328	0.04356	
ENSG000002	RN7SL246P	RNA, 7SL, cy	1.9955	0.0081362	0.043567	
ENSG000002	AL080276.2	novel transc	2.9055	0.0081668	0.043705	
ENSG000002	KRT18P61	keratin 18 p	2.2855	0.0081687	0.043708	
ENSG000002	AC007365.1	novel transc	2.2459	0.0081734	0.043727	
ENSG000002	FTH1P1	ferritin heav	2.4273	0.0081749	0.043728	
ENSG000002	AC008953.1	ribosomal p	1.5022	0.0081854	0.043778	
ENSG000002	AC008813.1	poly(A) binc	4.7833	0.0081968	0.043832	
ENSG000002	AC004974.1	novel transc	2.4023	0.0082041	0.043859	
ENSG000002	PCA3	prostate car	1.4584	0.0082166	0.043906	
ENSG000001	SPIN2A	spindlin fam	1.9135	0.008238	0.044	GO:0005634
ENSG000002	AC012066.1	nuclear distr	1.3681	0.0082457	0.044035	
ENSG000002	SMC2-AS1	SMC2 antise	4.2449	0.0082575	0.044078	

ENSG000002	AP002961.1	novel transc	2.1053	0.0082618	0.044095	
ENSG000002	AP000907.3	ATP synthas	4.7281	0.0082665	0.044113	
ENSG000002	OPA1-AS1	OPA1 antise	1.7367	0.0082775	0.044152	
ENSG000002	ZNF582-AS1	ZNF582 anti	1.6506	0.0083228	0.044367	
ENSG000002	EEF1B2P1	eukaryotic t	2.5974	0.0083292	0.044388	
ENSG000002	LINC01521	long interge	-1.2838	0.0083336	0.044405	
ENSG000002	AC110792.2	novel transc	2.1792	0.008364	0.044527	
ENSG000002	RPS16P5	ribosomal p	1.3963	0.0084037	0.044725	
ENSG000002	AL050343.2	novel transc	3.0954	0.0084183	0.04479	
ENSG000001	ALPK2	alpha kinase	1.037	0.0084294	0.044842	GO:0004674
ENSG000001	RN7SKP74	RNA, 7SK sn	1.3795	0.0084349	0.044864	
ENSG000002	LINC01794	long interge	2.7213	0.008478	0.045042	
ENSG000002	AC006059.1	novel transc	1.0984	0.0084775	0.045042	
ENSG000002	AC063944.1	novel transc	1.4619	0.0084766	0.045042	
ENSG000002	AC008937.3	novel transc	1.1087	0.0084764	0.045042	
ENSG000002	AL513327.2	uncharacteri	2.7404	0.0084954	0.045126	
ENSG000002	KRT18P7	keratin 18 ps	1.5667	0.0084989	0.045131	
Novel.5428	-	-	-1.0773	0.0085258	0.045241	
ENSG000001	IDI2	isopentenyl	1.9669	0.008556	0.045381	GO:0046872
ENSG000002	ZNF92P3	zinc finger p	1.2609	0.0085749	0.045445	
ENSG000001	IGFN1	immunoglol	-3.8935	0.008584	0.045475	GO:0005634
ENSG000002	RNY4P36	RNA, Ro-ass	2.6097	0.0086025	0.045547	
ENSG000002	AC104763.2	H3 histone,	3.0013	0.0086157	0.045583	
ENSG000002	AC009041.4	novel transc	1.3441	0.0086257	0.045622	
ENSG000002	Z84478.1	tRNA nucleo	2.085	0.0086424	0.045697	
ENSG000002	AL034550.2	uncharacteri	3.6481	0.0087121	0.046031	
ENSG000002	AC002400.1	cyclin Y-like	1.647	0.0087173	0.046045	
ENSG000002	CHCHD4P5	coiled-coil-l	1.7722	0.0087333	0.046123	
ENSG000002	AP000786.1	novel transc	1.246	0.0087356	0.046128	
ENSG000002	RNA5SP48	RNA, 5S ribo	1.9964	0.0087503	0.046193	
ENSG000001	MMP17	matrix meta	-1.1786	0.0087701	0.04627	GO:0016020
ENSG000002	PABPC1P1	poly(A) binc	1.231	0.008796	0.046379	
ENSG000000	MPP4	membrane p	-1.1253	0.0088078	0.046434	GO:0005737
ENSG000002	AL162431.3	pseudogene	1.3986	0.0088132	0.046456	
ENSG000002	C21orf62-AS	C21orf62 an	2.2193	0.0088316	0.046533	
ENSG000002	RN7SL459P	RNA, 7SL, cy	2.6213	0.0088567	0.04663	
ENSG000002	AC009303.1	nuclear facto	2.9361	0.008859	0.046636	
ENSG000002	HNRNPA1P(	heterogeneo	3.6254	0.0088811	0.046725	
ENSG000002	LINC01845	long interge	4.7281	0.0089184	0.046886	
ENSG000001	FNDC11	fibronectin t	2.552	0.008945	0.047013	GO:0005515
Novel.6200	-	-	-1.473	0.008963	0.047079	
ENSG000002	AC005856.1	novel transc	2.674	0.0089805	0.047158	
ENSG000002	TPM1-AS	TPM1 antise	1.672	0.0090415	0.047457	
ENSG000002	AL031663.3	novel transc	4.7141	0.0090811	0.047644	
ENSG000002	C1orf220	chromosom	1.2053	0.0091057	0.047752	
ENSG000002	ARL4AP4	ADP ribosyl	1.52	0.0091092	0.047756	
ENSG000002	RN7SL530P	RNA, 7SL, cy	1.6576	0.0091128	0.047768	
ENSG000002	DPYD-AS2	DPYD antise	1.4987	0.0091241	0.047821	

ENSG000002	MIR3173	microRNA 3	4.1958	0.0091813	0.048106	GO:0005615
ENSG000002	ZNF101P2	zinc finger p	2.7581	0.0091922	0.048133	
ENSG000002	RCC2P4	regulator of	1.7141	0.0091923	0.048133	
ENSG000002	RNU6-1214I	RNA, U6 sm	2.3965	0.0091944	0.048133	
ENSG000002	MT-TE	mitochondr	1.6113	0.0092358	0.048328	
ENSG000002	AC019070.1	Ubiquitin ca	2.5358	0.0092845	0.048526	
ENSG000002	MAGI1-AS1	MAGI1 antis	2.1861	0.0092871	0.048533	
ENSG000002	EEF1A1P3	eukaryotic t	1.2977	0.0093468	0.048816	
ENSG000002	AL591719.1	chromosom	2.3866	0.0093779	0.048964	
ENSG000002	AC130324.1	novel transc	1.37	0.0094165	0.049108	
ENSG000001	DENND2D	DENN doma	2.8083	0.0094564	0.049295	GO:0005737
ENSG000002	CCNB1IP1P1	cyclin B1 int	2.6121	0.0094994	0.049478	
ENSG000002	RPAP2P1	RNA polyme	2.0056	0.0094962	0.049478	
ENSG000002	PPIAP51	peptidylpro	1.2227	0.0095011	0.049478	
ENSG000002	AL356805.1	novel transc	3.2285	0.0095249	0.04958	
ENSG000002	MIR3679	microRNA 3	2.9373	0.0095492	0.04969	
ENSG000002	AP001469.1	novel transc	1.4776	0.0095569	0.049718	
ENSG000001	MYRIP	myosin VIIA	2.5322	0.0095668	0.049762	GO:0005737
ENSG000002	AC027130.1	uncharacteri	2.2141	0.0095835	0.049842	
ENSG000002	RPS3AP47	ribosomal p	-1.4644	0.009608	0.049955	

GO annotati	KO ID	pathway ID	pathway nar	TF family	siPDIA-1 co	siPDIA-nc count
RNA bindin	hsa:2923	hsa04141;hs	Protein processing in		572.93	3264.89
nucleus;cytc	hsa:3939	hsa00010;hs	Glycolysis / Gluconec		11273.35	23163.17
positive reg	hsa:595	hsa01522;hs	Endocrine resistance		817.46	3200.14
cytoplasm;c	hsa:3925	hsa04010;hs	MAPK signaling path		1572.6	4316.43
					17809.28	8305.02
positive reg	hsa:3688	hsa04015;hs	Rap1 signaling pathw		5666.27	11810.33
nucleus;nucleus;cytoplasm;cytosol;perinuclear region of cy					1646.78	3581.96
membrane;i	hsa:3675	hsa04151;hs	PI3K-Akt signaling pa		2496.67	5822.59
nucleus;cytoplasm;nucleus;cytosol;fibrillar center;cytoskele					1349.73	2955
negative reg	hsa:5621	hsa04216;hs	Ferroptosis ;Prion dis		1071.1	2408.64
nucleus;mer	hsa:7416	hsa04020;hs	Calcium signaling pa		1711.14	3440.64
positive reg	hsa:960	hsa04512;hs	ECM-receptor interac		2342.46	4992.7
membrane;i	hsa:6382	hsa04512;hs	ECM-receptor interac		599.7	1521.23
cell migratic	hsa:5420	hsa05132	Salmonella infection		1807.54	3758.23
nucleus;nuc	hsa:1017	hsa04068;hs	FoxO signaling pathv		1001.58	2193.99
Golgi apparatus;Golgi apparatus;protein binding;extracellu					1851.55	3892.76
positive regulation of transcription by RNA polyr				HMGA	3451.9	7355.08
nucleus;nucleolus;nucleus;cytoplasm;nucleus;nucleolus;pr					695.48	1714.39
					766.79	222.16
nucleus;nucleus;cytoplasm;cytoplasm;cytoplasm;membran					392.67	1059.06
membrane;i	hsa:81537	hsa00600;hs	Sphingolipid metabo		885.52	1936.22
extracellular	hsa:1292	hsa04151;hs	PI3K-Akt signaling pa		2375.13	5076.31
extracellular	hsa:5054	hsa04066;hs	HIF-1 signaling pathv		641.59	1659.81
gap junctior	hsa:2697	hsa04540;hs	Gap junction ;Arrhyth		316.88	937.95
nucleus;nuc	hsa:6472	hsa00260;hs	Glycine, serine and th		1901.58	4031.05
					2205.6	955.43
nucleus;cytoplasm;cytoplasm;membrane;integral compone					6014.4	2845.61
membrane;i	hsa:4267	hsa04514;hs	Cell adhesion molecu		816.54	1688.9
positive reg	hsa:3572	hsa04060;hs	Cytokine-cytokine re		1493.92	3096.81
nucleus;nuc	hsa:8061	hsa04310;hs	Wnt signalir	TF_bZIP	654.51	1789.07
cytoplasm;n	hsa:2182	hsa00061;hs	Fatty acid biosynthes		976.25	1983
nucleus;DNA binding;protein heterodimerization activity;p					867.84	1757.04
cardiac mus	hsa:7273	hsa05410;hs	Hypertrophic cardion		869.76	326.47
nucleus;cell	hsa:900	hsa04115;hs	p53 signaling pathwa		1059.36	2133.02
positive reg	hsa:4067	hsa04062;hs	Chemokine signaling		340.99	869.08
					901.05	332.26
					1328.12	546.32
apoptotic process;autophagy;death domain binding;negat					611.11	1296.28
membrane;integral component of membrane;endoplasmic					318.26	814.4
membrane;integral component of membrane;plasma mem					649.86	1335.34
RNA bindin	hsa:1728	hsa00130;hs	Ubiquinone and othe		958.12	2164.14
cytosol;nucl	hsa:4907	hsa00230;hs	Purine metabolism ;P		544.92	1240.82
cytoplasm;tı	hsa:6164	hsa03010	Ribosome		715.32	1537.33
					475.68	130.81
membrane;c	hsa:1291	hsa04151;hs	PI3K-Akt signaling pa		2413.04	4885.39
extracellular	hsa:6422	hsa04310	Wnt signaling pathwa		281.17	734

RNA binding;nucleus;nucleus;nucleolus;mitochondrion;nu					544.6	1177.29
nucleus;cytc	hsa:3839	hsa05132	Salmonella infection		719.43	1448.4
mitochondrion;mitochondrion;apoptotic process;mitochor					44.6	286.07
					358.1	84.41
isomerase activity;peptidyl-prolyl cis-trans isomerase activ					593.08	1246.48
nucleus;cytoplasm;membrane;membrane;cytosol;nucleopla					301.26	718.65
metal ion bi	hsa:7052	hsa05016	Huntington disease		105.74	430.21
membrane;integral component of membrane;cell adhesion					588.93	1256.44
negative regulation of endothelial cell proliferatic				RXR-like	464.03	981.22
nucleus;cytc	hsa:10318	hsa05131	Shigellosis		329.41	739.49
nucleus;metal ion binding;transferase activity;protein ubiq					358.79	838.23
negative reg	hsa:7078	hsa05205;hs	Proteoglycans in canc		306.01	721.76
extracellular	hsa:7473	hsa04150;hs	mTOR signaling path		336.86	749.95
					444.17	142.63
					242.67	722.64
cell adhesio	hsa:3914	hsa04151;hs	PI3K-Akt signaling pa		224.63	624
					273.82	72.78
membrane;l	hsa:123	hsa03320	PPAR signaling pathv		488.72	1209.74
cytoplasm;m	hsa:65108	hsa04666;hs	Fc gamma R-mediate		326.83	767.37
membrane;i	hsa:64065	hsa04115	p53 signaling pathwa		267.69	621.49
cytosol;regu	hsa:2730	hsa00270;hs	Cysteine and methior		338.07	798.09
membrane;i	hsa:5329	hsa04610;hs	Complement and coa		279.78	675.28
positive reg	hsa:1490	hsa04371;hs	Apelin signaling path		573.67	1331.38
extracellular space;protein binding;extracellular region;extra					124.93	413.6
cytoplasm;n	hsa:8877	hsa00600;hs	Sphingolipid metabo		169.77	521.31
nucleus;cytc	hsa:27018	hsa04722	Neurotrophin signali		394.07	837.47
protein kinase activity;ATP binding;protein phosphorylatic					375.55	812.35
membrane;integral component of membrane					433.64	869.76
					303.92	81.82
nucleic acid	hsa:91120	hsa05168	Herpes simp	zf-C2H2	521.73	210.72
negative reg	hsa:51129	hsa03320;hs	PPAR signaling pathv		176.31	576.11
membrane;r	hsa:1535	hsa04145;hs	Phagosome ;Osteocla		211.71	583.63
					239.61	591.83
negative reg	hsa:55869	hsa05034;hs	Alcoholism ;Viral carc		698.54	343.74
cytoplasm;tı	hsa:6235	hsa03010	Ribosome		534.23	1173.17
intracellular;	hsa:2250	hsa04010;hs	MAPK signaling path		226.45	549.06
					563.07	258.52
membrane;metal ion binding;calcium ion binding;membra					429.48	911.9
RNA bindin	hsa:6624	hsa05206	MicroRNAs in cancer		418.18	882.39
metal ion binding;methylated histone binding;negative reg					351.38	728.38
					407.98	139.31
					405.65	123.53
cytoplasm;si	hsa:4660	hsa04270;hs	Vascular smooth mus		641.96	314.82
RNA binding;nucleus;nucleus;nucleolus;DNA binding;nucl					361.45	808.94
					331.17	107.15
nucleic acid binding;RNA binding;RNA binding;mRNA pro					210.31	482.59
membrane;i	hsa:3673	hsa04145;hs	Phagosome ;PI3K-Ak		341.79	692.64
membrane;hydrolase activity;plasma membrane;phosphop					155.75	398

cytoplasm;cytoplasm;membrane;cytosol;plasma membrane					222.06	486.53
growth facto	hsa:3589	hsa04060;hs	Cytokine-cytokine re		354.46	820.7
nucleus;cytoplasm;cell redox homeostasis;oxidation-reduc					282.27	583.41
extracellular space;nucleus;cytoplasm;zinc ion binding;DN					354.32	781.5
					284.48	83.72
membrane;r	hsa:11031	hsa04144	Endocytosis		219.75	509.47
transcription, DNA-templated;negative regulation of phos					74.87	239.25
membrane;integral component of membrane;proteolysis;n					186.34	52.37
					131.11	24.53
RNA binding;nucleus;DNA binding;chromatin DNA bindin					150.62	24.49
nucleus;cytoplasm;cytosol;nucleoplasm;cytosol;protein bin					278.25	569.95
negative regulation of canonical Wnt signaling pathway;ex					248.03	513.47
methyltrans	hsa:2593	hsa00260;hs	Glycine, serine and th		86.72	265.84
membrane;integral component of membrane;transporter a					308.32	645.99
					149.36	34.7
cytoplasm;membrane;protein transport;nucleotide binding					135.36	326.29
membrane;i	hsa:1949	hsa04360	Axon guidance		138.62	344.89
					55.82	192.63
					221.14	70.17
nucleotide binding;GTPase activity;GTP binding;intracellula					192.82	60.51
RNA binding;translation;ribosome;mitochondrion;mitocho					185.51	413.43
					295.27	112.27
metal ion bi	hsa:27445	hsa04911	Insulin secretion		192.19	59.25
					392.76	192.32
					87.42	13.23
nucleic acid binding;RNA binding;zinc ion binding;metal ic					144.18	337.42
cell adhesion;extracellular region;collagen-containing extra					84.82	11.04
cytoplasm;n	hsa:5737	hsa04020;hs	Calcium signaling pa		137.71	326.27
cytoplasm;c	hsa:127281	hsa00590;hs	Arachidonic acid met		100.25	262.72
					399.48	196.49
					162.67	41.71
					243.01	98.52
					272.95	107.89
cytoplasm;h	hsa:1849	hsa04010	MAPK signaling path		215.53	453.15
membrane;i	hsa:290	hsa00480;hs	Glutathione metaboli		171.17	391.64
nucleic acid binding;regulation of transcription, D				zf-C2H2	244.58	100.19
nucleus;cytoplasm;metal ion binding;calcium ion binding;r					151.29	43.57
					294.84	118.3
					303.38	137.7
extracellular space;growth factor activity;regulation of signa					144.65	41.06
					304.98	139.32
membrane;plasma membrane;nervous system developmen					29.14	130.49
membrane;i	hsa:8829	hsa04360;hs	Axon guidance ;Hum		126.6	293.01
hydrolase activity;DNA binding;chromosome;meiotic cell c					223.89	86.46
					113.98	26.92
nucleotide binding;protein kinase activity;protein serine/th					99.54	244.59
intracellular	hsa:200916	hsa03010	Ribosome		139.26	309.18
calcium ion binding;extracellular region;collagen binding;c					100.51	21.36

membrane;integral component of membrane;plasma mem				123.64	28.97
				103.09	20.8
RNA binding;nucleus;nucleus;DNA binding;chromatin DN/				339.73	136.99
cytoplasm;c	hsa:8835	hsa04630;hs	JAK-STAT signaling p	81.45	217.64
nucleic acid	hsa:158431	hsa05168	Herpes simp zf-C2H2	255.06	109.6
nucleus;cytc	hsa:4502	hsa04978	Mineral absorption	169.23	365.69
calcium ion binding;cell adhesion;extracellular region;extra				142.29	311.4
negative regulation of apoptotic process;membrane;integr				183.49	401.91
nucleus;nuc	hsa:440689	hsa05034;hs	Alcoholism ;Viral carc	133.55	28.1
				470.42	219
				143.03	28.93
membrane;integral component of membrane;intracellular r				144.46	324.88
membrane;i	hsa:3679	hsa04151;hs	PI3K-Akt signaling pa	261.97	555.05
extracellular	hsa:1839	hsa01522;hs	Endocrine resistance	114.45	270.75
				93.84	242.74
cytoplasm;membrane;plasma membrane;plasma membran				106.49	295.1
membrane;integral component of membrane;membrane;ir				204.89	78.55
membrane;metal ion binding;calcium ion binding;protein I				205.6	416.31
nucleus;nucleus;metal ion binding;nucleoplasm;transcripti				176.18	355.98
nucleic acid binding;nucleus;nucleus;cytoplasm;m ZBTB				454.78	223.63
nucleus;nucleus;cytoplasm;hydrolase activity;protein homc				153.87	322.12
cytoplasm;m	hsa:3357	hsa04020;hs	Calcium signaling pa	66.15	8.71
membrane;integral component of membrane;intrinsic com				46.36	152.1
				338.62	167.06
membrane;integral component of membrane;transmembra				164.13	59.17
membrane;integral component of membrane;integral comp				150.31	318.29
extracellular region;heparin binding;extracellular matrix;cell				168.23	60.54
nucleus;cytoplasm;cytosol;nucleoplasm;protein binding				167.32	341.44
				108.47	24.84
				116.7	31.72
transcriptior	hsa:57761	hsa04931	Insulin resistance	251.34	528.58
intracellular;membrane;integral component of membrane;(				116.73	264.48
				294.08	135.35
membrane;r	hsa:1160	hsa00330;hs	Arginine and proline	130.3	38.57
membrane;integral component of membrane;plasma mem				82.73	16
membrane;integral component of membrane;cytoskeleton				241.4	108.6
membrane;i	hsa:2043	hsa04360	Axon guidance	315.86	149.64
metal ion binding;hydrolase activity;lyase activity;peptidas				183.4	68.78
				100.47	26.38
				213.07	83.91
				114.08	31.79
cytosol;nucleoplasm;protein dimerization activity; bHLH				219.24	438.78
cytoplasm;cytoplasm				282.61	130.43
nucleus;nuc	hsa:8349	hsa05034;hs	Alcoholism ;Viral carc	198.25	74.08
				117.47	28.72
				215.44	82.59
				78.05	15.39
				67.12	10.74

membrane;i	hsa:93183	hsa00563;hs	Glycosylphosphatidy	122.14	264.83
				172.46	59.94
				95.59	23.38
				80.41	17.52
cytoplasm;c	hsa:3945	hsa00010;hs	Glycolysis / Gluconec	145.63	305.83
membrane;integral component of membrane;peptide bind				13.12	83.69
				269.51	115.67
cytoplasm;n	hsa:11343	hsa00561;hs	Glycerolipid metaboli	68.04	180.83
				85.86	20.7
positive regulation of transcription by RNA polym			Fork_hea	112.5	255.96
protein binding;protein binding;protein binding				364.63	168.57
nucleic acid binding;nucleus;metal ion binding;DI			zf-C2H2	256.95	116.67
growth facto	hsa:6387	hsa04060;hs	Cytokine-cytokine re	157.79	344.43
endoplasmi	hsa:7869	hsa04360	Axon guidance	133.59	278.98
nucleus;cytoplasm;metal ion binding;protein binding;nucle				82.31	214.14
				116.42	34.47
				222.95	92.8
nucleus;hyd	hsa:1847	hsa04010	MAPK signaling path	63.97	166.34
RNA binding;nucleus;nucleus;DNA binding;chromatin DN/				326.87	56.89
				77.28	17.28
				95.7	26.23
membrane;integral component of membrane;cell adhesion				141.37	52.2
				122100.82	25993.06
nucleus;cytoplasm;zinc ion binding;metal ion binding;cyto				139.77	280.11
membrane;integral component of membrane;actin binding				80.57	19.56
				79.62	14.9
RNA bindin	hsa:8367	hsa05034;hs	Alcoholism ;Viral carc	144.91	35.32
				147.78	55.54
				227.84	109.81
cytoplasm;cytoplasm;membrane;protein binding;protein b				40.57	123.13
				194.18	88.14
				83.31	21.55
nucleotide binding;ATP binding;nucleotide binding;ATP bi				225.04	109.97
				135.26	50.07
				252455.63	80973.19
membrane;cell adhesion;plasma membrane;plasma membr				142.17	53.99
				143.05	54.49
nucleus;nuc	hsa:4616	hsa04010;hs	MAPK signaling path	126.89	271.54
nucleus;nuc	hsa:3017	hsa05034;hs	Alcoholism ;Viral carc	281.46	130.71
extracellular	hsa:5360	hsa03320;hs	PPAR signaling pathv	121.77	244.69
protein binding;cytoplasmic microtubule;cytoplasmic micro				140.92	290.1
				50.5	7.25
nucleus;nucleus;regulation of transcription, DNA-			TF_bZIP	220.94	450.09
nucleus;regulation of transcription, DNA-templat			Pou	77.85	20.36
RNA binding;nucleus;nucleus;chromatin organization;DNA				124.67	33.27
				219	102.27
				106.66	222.47
				149.11	62.66

serine-type	hsa:5328	hsa04064;hs	NF-kappa B signalin		75.35	183.57
nucleic acid binding;metal ion binding;regulation				zf-C2H2	190.72	90.61
nucleus;nuc	hsa:2118	hsa05202	Transcriptio	ETS	47.48	144.29
					139.52	53.46
structural m	hsa:3885	hsa04915;hs	Estrogen signaling pa		19.07	90.14
positive reg	hsa:3303	hsa03040;hs	Spliceosome ;MAPK s		112.49	247.88
nucleus;nucleus;cytoplasm;cytoplasm;cytosol;mitochondri					228.46	111.63
					116.56	42.67
					73.54	18.72
					109.67	234.38
nucleic acid	hsa:9310	hsa05168	Herpes simp	zf-C2H2	127.91	50.02
					117.32	40.51
nucleus;cytc	hsa:2324	hsa04010;hs	MAPK signaling path		102.37	214.65
nucleus;mer	hsa:489	hsa04020;hs	Calcium signaling pa		58.71	151.15
nucleic acid binding;regulation of transcription, D				zf-C2H2	199.87	96.87
					105.79	36.01
					89.86	23.94
					59.99	12.35
					128727.18	59385.13
					57.84	12.71
					83.93	183.96
					65.12	14.46
cytoplasm;membrane;integral component of membrane;pr					60.56	13.76
cytoplasm;carbohydrate binding;carbohydrate binding;grc					155.42	313.13
positive reg	hsa:3304	hsa03040;hs	Spliceosome ;MAPK s		1432.72	2953.27
cytoplasm;transferase activity;protein ubiquitination;protei					190.76	93.54
nucleus;nuc	hsa:3012	hsa04217;hs	Necroptosis ;Alcoholi		100.62	24.77
					14.53	66.45
membrane;integral component of membrane					101.08	34.55
nucleus;nuc	hsa:1846	hsa04010	MAPK signaling path		39.87	113.25
					62.04	148.52
					56.68	12.53
extracellular region;insulin-like growth factor binding;regu					3.06	42.77
					52.36	11.01
					48.35	9.45
membrane;r	hsa:1577	hsa00140;hs	Steroid hormone bio:		98.43	34.5
positive reg	hsa:6662	hsa04024	cAMP signa	HMG	26.58	93.51
cytoplasm;n	hsa:10125	hsa04010;hs	MAPK signaling path		112.53	42.7
growth facto	hsa:3625	hsa04060;hs	Cytokine-cytokine re		39.3	111.38
					189.25	82.92
extracellular region;extracellular region;molecular_function					36.77	4.35
					60.74	13.33
					44.87	8.23
					130.28	55.36
extracellular region;protein binding;insulin-like growth fac					65.89	149.47
RNA binding;nucleus;nucleus;nucleolus;nucleopla				ARID	82.55	176.79
					42.36	6.41
membrane;integral component of membrane;calcium ion b					55.35	12.83

intracellular;regulation of signaling receptor activity;extrace					44.78	116.65
					68.01	19.9
extracellular region;signaling receptor binding;transmembr					76.22	20.82
					137.47	55.59
					65.75	16.53
negative reg	hsa:3728	hsa05200;hs	Pathways in cancer ;T		58.96	147.5
membrane;(	hsa:2793	hsa04014;hs	Ras signaling pathwa		42.76	111.05
membrane;r	hsa:4729	hsa00190;hs	Oxidative phosphory		80.09	25.31
					140.4	61.25
					98.06	36.6
nucleus;nuc	hsa:8350	hsa05034;hs	Alcoholism ;Shigellos		31.64	3.06
					70.49	20.95
membrane;integral component of membrane;transferase a					79.09	26.49
nucleic acid	hsa:353355	hsa05168	Herpes simp	zf-C2H2	76.99	23.51
nucleus;nuc	hsa:8329	hsa04217;hs	Necroptosis ;Alcoholi		60.86	12.33
					47.92	9.63
nucleus;nuc	hsa:8347	hsa05034;hs	Alcoholism ;Viral carc		84.38	21.61
nucleus;nuc	hsa:8339	hsa05034;hs	Alcoholism ;Viral carc		121.03	36.6
					59.52	14.12
nucleus;nucleus;cytoplasm;cytoplasm;endoplasmic reticulu					145.71	66.77
membrane;plasma membrane;cell junction;synapse;postsy					75.01	23.85
					82.07	22.84
extracellular region;extracellular space;extracellular space;ex					46.34	116.64
					40.63	6.28
					38.03	5.76
					126.3	53.12
					82.03	27.53
nucleus;cyto	hsa:9863	hsa04015;hs	Rap1 signaling pathw		180.66	85.67
					57.73	14.5
					65.62	17.8
					83.53	25.18
membrane;integral component of membrane;integral comp					54.11	12.21
nucleus;nucleus;metal ion binding;transcription b				Others	59.83	14.63
					37.46	6.37
					27.9	85.64
					102.2	40.29
extracellular space;extracellular space;protein binding;extra					76.09	161.29
					85.19	29.99
dephosphor	hsa:30833	hsa00230;hs	Purine metabolism ;P		83.08	168.35
					61.2	17.29
					42.91	8.14
protein binding;extracellular region;leukocyte migration;ex					63.94	17.05
protein binding					78.8	165.37
					37.85	6.41
					87.04	31.37
membrane;i	hsa:10161	hsa04072;hs	Phospholipase D sigr		74.35	23.39
cytoplasm;n	hsa:56171	hsa05014;hs	Amyotrophic lateral s		130.76	59.98
nucleus;nucleus;cytoplasm;cytoskeleton;protein binding;ce					132.07	264.62

nucleus;cytc	hsa:347733	hsa04145;hs	Phagosome ;Gap jun	59.68	141.05
				146.71	71.29
				58.08	129.58
				97.06	34.36
positive reg	hsa:51176	hsa04310;hs	Wnt signalir HMG	20.02	69.91
				72.92	23.11
				42.78	8.76
				98.86	38.41
membrane;integral component of membrane;calcium ion b				63.34	19.58
				31.75	4.33
positive reg	hsa:783	hsa04010;hs	MAPK signaling path	108.52	46.88
				50.48	12.4
				35.1	5.07
				60.45	16.76
				70.99	150.14
				53.67	13.49
				160.95	79.05
				35.51	5.76
growth facto	hsa:2919	hsa04060;hs	Cytokine-cytokine re	8.4	45.33
membrane;i	hsa:3776	hsa04927;hs	Cortisol synthesis anc	47.75	112.65
membrane;i	hsa:8707	hsa00601;hs	Glycosphingolipid bi	59.97	17.58
				168.62	77.18
cytoplasm;cytoplasm;membrane;integral component of membrane;transmembrane transport;i					
				41.43	8.11
membrane;i	hsa:51208	hsa04514;hs	Cell adhesion molecu	76.34	25.06
nucleic acid binding;nucleus;metal ion binding;DN			zf-C2H2	108.73	218.25
				397.98	106.37
				63.35	18.89
				135.54	55.16
				49.21	12.31
cytoplasm;n	hsa:1768	hsa05014;hs	Amyotrophic lateral s	103.99	42.01
				111.53	49.39
				45.95	10.86
positive reg	hsa:8792	hsa04060;hs	Cytokine-cytokine re	56.14	123.25
				35.33	5.93
membrane;integral component of membrane;voltage-gate				44.44	108.11
				81.72	31.09
				55.74	15.45
membrane;integral component of membrane;membrane;ir				132.35	63.56
nucleic acid binding;regulation of transcription, DNA-temp				56.71	15.55
				74.44	26.81
nucleus;nuc	hsa:8352	hsa05034;hs	Alcoholism ;Shigellos	34.01	5.02
nucleus;nuc	hsa:8332	hsa04217;hs	Necroptosis ;Alcoholi	68.77	15.71
				125.64	57.37
				76.1	25.54

					43.03	10.15
					75.88	25.01
					56.05	16.51
nucleic acid	hsa:54753	hsa05168	Herpes simp	zf-C2H2	35.19	91.02
					119.34	51.71
					140.35	69.94
cytoplasm;n	hsa:10846	hsa00230;hs	Purine metabolism ;N		137.54	66.85
membrane;i	hsa:3769	hsa04974	Protein digestion anc		53.54	14.48
cytoplasm;c	hsa:1848	hsa04010;hs	MAPK signaling path		40.42	105.43
					40.71	9.26
					88.14	36.3
cytoplasm;c	hsa:998	hsa04010;hs	MAPK signaling path		74.88	27.53
					70.81	24.84
					26.15	3.03
cytoplasm;actin binding;cytoskeleton;protein binding;actir					111.07	48.53
GTPase regulator activity;regulation of catalytic activity;cyt					83.3	168.82
					7.15	40.07
membrane;i	hsa:259293	hsa04742	Taste transduction		27.49	3.51
membrane;i	hsa:81466	hsa04740	Olfactory transductio		25.51	2.52
GTPase activ	hsa:5874	hsa04972	Pancreatic secretion		42.91	104.36
					66.28	22.09
phosphatid	hsa:3613	hsa00562;hs	Inositol phosphate m		68.32	144.68
nucleus;nuc	hsa:8348	hsa05034;hs	Alcoholism ;Viral carc		46.13	10.69
					58.45	15.26
					71.29	25.91
					74.18	28.13
					34.64	5.96
					107.23	48.36
membrane;integral component of membrane;calcium ion b					38.77	8.69
membrane;integral component of membrane;G protein-co					36.34	7.68
nucleus;nuc	hsa:8345	hsa05034;hs	Alcoholism ;Viral carc		102.41	40.92
kinase activity;phosphorylation;calmodulin binding;phosp					15.95	55.47
					24.58	2.55
membrane;g	hsa:5228	hsa04010;hs	MAPK signaling path		34.92	87.39
					79.77	30.52
					78.11	30.84
nucleus;nuc	hsa:8342	hsa05034;hs	Alcoholism ;Viral carc		40.34	7.41
					118.55	56.93
nucleus;intracellular membrane-bounded organelle;metal					105.5	47.4
					109.14	48.74
intracellular;nucleotide binding;protein kinase activity;prot					60.08	126.57
membrane;r	hsa:54344	hsa00510;hs	N-Glycan biosynthes		43.98	102.18
intracellular	hsa:25759	hsa01521;hs	EGFR tyrosine kinase		49.68	112.09
nucleus;nuc	hsa:8343	hsa05034;hs	Alcoholism ;Viral carc		58.2	12.97
					89.83	37.33
positive regulation of endothelial cell migration;p				Fork_hea	43.63	103.73
					47.77	12.39
extracellular	hsa:3053	hsa04610	Complement and coa		45.12	12.29

membrane;integral component of membrane;integral comp					45.87	104.1
nucleus;metal ion binding;nucleolus;nucleus;metal ion bin					75.69	29.19
					24.25	2.5
protein kina	hsa:729597	hsa04114;hs	Oocyte meiosis ;Prog		109.29	51.88
					90.66	37.11
					33.73	6.93
					38.57	8.33
extracellular region;female pregnancy					31.86	5.88
membrane;i	hsa:259290	hsa04742	Taste transduction		35.19	7.62
					67.56	24.97
					17.54	57.73
RNA bindin	hsa:8365	hsa05034;hs	Alcoholism ;Viral carc		128.9	52.09
positive reg	hsa:1958	hsa04371;hs	Apelin signa	zf-C2H2	134.34	497.94
					35.83	7.61
					33.23	6.46
membrane;i	hsa:338707	hsa00513;hs	Various types of N-g		68.92	138.93
					42.16	10.97
oxidoreduct	hsa:217	hsa00010;hs	Glycolysis / Gluconeo		44.88	102.62
					43.4	11.6
membrane;integral component of membrane;calcium ion b					42.03	9.86
					27.28	3.6
membrane;integral component of membrane;transmembra					106.91	51.39
cytoplasm;cytoskeleton;protein binding;perinuclear region					15.76	54.78
					113.85	51.86
					20.44	1.56
					44.34	100.97
membrane;integral component of membrane;calcium ion b					103.56	47.17
membrane;i	hsa:9934	hsa04080	Neuroactive ligand-r		34.43	6.43
nucleus;nuc	hsa:8346	hsa05034;hs	Alcoholism ;Viral carc		39.88	8.65
membrane;integral component of membrane;calcium ion b					65.77	24.4
nucleus;nucleus;cytoplasm;cytoplasm;nucleoplasm;tRNA p					59.18	124.72
metal ion bi	hsa:4832	hsa00230;hs	Purine metabolism ;P		47.71	108.81
cytoplasm;nucleotide binding;ATP binding;motor activity;a					30.54	81.86
					30.78	4.99
extracellular	hsa:27329	hsa04979	Cholesterol metaboli		54.35	16.91
					73.58	25.11
					36.82	8.68
					50.36	16.08
					94.57	42.4
					96.52	43.57
					67.53	26.14
					28.7	5.12
					27.03	4.55
nucleus;nuc	hsa:8358	hsa05034;hs	Alcoholism ;Shigellos		123.36	21.57
					37.95	9.05
transformin	hsa:2353	hsa01522;hs	Endocrine re	TF_bZIP	55.62	126.3
cytoplasm;cytoskeleton;protein binding;centrosome;micro					57.58	116.16
membrane;c	hsa:6900	hsa04514	Cell adhesion molecu		35.56	8.33

					38.36	8.95
nucleic acid binding;nucleus;metal ion binding;DN				zf-C2H2	43.86	12.84
membrane;i	hsa:64101	hsa04360;hs	Axon guidance ;Cell a		56.35	18.3
protein binding;protein binding;protein binding;protein bi					56.72	116.58
					49.69	15.11
					58.4	19.64
nucleic acid binding;nucleus;DNA binding;actin filament bi					58.26	118.64
					47.82	14.76
					41.02	11.1
					65.56	24.64
					6483.82	1862
nucleic acid	hsa:163050	hsa05168	Herpes simp	zf-C2H2	73.69	28.21
					31.09	6.14
					27.58	5.12
					87.05	36.76
					40.97	10.54
membrane;i	hsa:6554	hsa04976;hs	Bile secretion ;Hepati		42.71	10.21
extracellular region;female pregnancy;extracellular region;f					122.96	58.53
extracellular region;neuropeptide signaling pathway;molec					39.71	93.03
molecular_function;cellular_component;nervous system de					23.82	83.25
extracellular space;metal ion binding;calcium ion binding;s					22.12	2.8
					78.91	31.49
					43.19	11.57
signaling re	hsa:7477	hsa04150;hs	mTOR signaling path		11.12	42.64
					1256.19	100.04
cytoplasm;cytoplasm;cytoplasm					47.18	13.93
					26.82	4.63
endoplasmic reticulum;endoplasmic reticulum;Golgi appara					49.83	15.97
					67.61	25.46
nucleus;nuc	hsa:8354	hsa05034;hs	Alcoholism ;Shigellos		44.17	9.65
					37.42	9.03
membrane;integral component of membrane;plasma mem					38.68	10.26
membrane;integral component of membrane;calcium ion b					36.95	7.45
membrane;integral component of membrane;ion transport					45.38	98.08
protein binc	hsa:284340	hsa04060	Cytokine-cytokine re		34.27	8.24
					59.1	22.26
					31.4	5.77
nucleic acid	hsa:10794	hsa05168	Herpes simp	zf-C2H2	424.05	170.78
nucleus;nucleoplasm;nucleus;nucleus;DNA-binding transc					46.82	102.61
					51.43	15.56
					71.64	29.44
nucleus;nucleoplasm;transcription by RNA polym				HMG	38.61	88.35
					30.89	6.23
					41.46	11.93
protein binding;protein binding;protein binding;protein bi					64.98	25.78
					54.48	122.88
					59.46	21.34
					43.38	12.77

				63.46	22.22
				57.19	19.28
membrane;integral component of membrane;Wnt signalin				22.58	61.33
				61.04	17.32
metal ion bi	hsa:285	hsa04010;hs	MAPK signaling path	28.51	5.85
				33.27	7.7
				62.44	22.6
				24.49	3.33
				65.69	26.75
				49.47	15.97
				36.28	9.7
				32.96	7.07
metal ion binding;2 iron, 2 sulfur cluster binding;intracellul				53.97	109.3
				68.14	28.58
				24.48	4.15
				55.31	18.77
				29.14	5.59
				38.64	10.02
				64.42	25.66
protein binding;extracellular region;response to oxidative s				29.68	6.62
nucleus;cytoplasm;regulation of transcription, DNA-templa				27.72	68.69
				34.59	8.33
				49.7	15.83
nucleic acid binding;RNA binding;nucleic acid binding;RNA				75.41	32.72
				22.05	2.53
				19.76	2.3
				36.35	9.57
membrane;i	hsa:93589	hsa04010;hs	MAPK signaling path	35.83	80.97
				105.97	52.03
				32.55	8.11
				22.88	3.11
				30.57	6.55
				38.71	11.22
RNA bindin	hsa:8366	hsa05034;hs	Alcoholism ;Viral carc	44.15	11.73
nucleus;nuc	hsa:5798	hsa04940	Type I diabetes mellit	37.58	84.84
				85.46	39.4
cytoplasm;c	hsa:9108	hsa00562;hs	Inositol phosphate m	69.16	27.61
nucleus;nucleus;nucleus;cytoplasm;regulation of			HMG	49.02	104
				44.81	14.18
membrane;i	hsa:401494	hsa00062;hs	Fatty acid elongation	38.55	11.23
hydrolase activity;mitochondrion;molecular_function;biolo				45.24	97.21
cytoplasm;n	hsa:23057	hsa00760;hs	Nicotinate and nicoti	35.82	81.23
				23.55	3.86
				35.87	8.86
membrane;membrane;integral component of membrane;m				37.57	82.88
actin filamen	hsa:11151	hsa04145;hs	Phagosome ;Tubercu	23.06	63.11
nucleus;damaged DNA binding;single strand break repair;r				98.55	48.14
				16.26	1.04

membrane;integral component of membrane;calcium ion b				51.83	19.13
				33.88	8.82
				29.89	6.49
				44.1	13.91
extracellular space;positive regulation of ERK1 and ERK2 ca				26.94	67.36
intracellular	hsa:3576	hsa04060;hs	Cytokine-cytokine re	17.63	53.53
				30.87	7.63
				21.12	62.57
				65.1	27.6
				38.58	10.41
				57060.76	810.08
				47.62	16.67
				52.53	108.99
				53.02	18.83
				43.79	14.61
				30.84	7.73
				46.06	15.04
				49.85	16.09
				16.53	1.02
				19.75	2.26
protein binding;intermediate filament;keratin filament;cytc				1.95	22.48
				62.63	25.92
				63.35	25.67
				59.65	23.67
				32.74	7.98
				58.2	23.08
				51.06	17.2
				70.82	28.99
cytoplasm;membrane;nucleotide binding;ATP binding;plas				47.62	99.45
				89314.14	15295.95
				24.88	4.11
				25.6	5.19
				51.08	13.18
hydrolase activity;peptidase activity;proteolysis;extracellula				60.01	23.42
				23.65	4.57
				34.43	8.29
				23.91	4.19
				73.4	31.54
				56.81	22.17
cytoplasm;c	hsa:397	hsa04722;hs	Neurotrophin signali	39.46	83.95
oxidation-re	hsa:2877	hsa00480;hs	Glutathione metaboli	49.3	17.55
				87.87	42.99
				22.55	3.87
				40.6	11.5
				45.63	94.81
membrane;r	hsa:2657	hsa04060	Cytokine-cytokine re	40.69	87.62
				37.15	10.75
				43.8	13.99

				33.7	9.52
				20.61	2.84
				70.98	31.08
				53.6	20.22
				38.39	11.03
membrane;integral component of membrane;membrane;ir				48.17	96.27
				52.51	18.94
nucleus;nuc	hsa:225689	hsa04657	IL-17 signaling pathv	25.02	64.78
intracellular	hsa:8642	hsa04392	Hippo signaling path	49.67	99.99
				26.11	5.62
				36.97	11.14
				27.99	5.84
membrane;integral component of membrane;tRNA proces				63.87	26.49
				21.95	3.61
				20.07	3.1
				8.63	0
				83.84	40
				48.44	17.05
				41.74	13.59
				140749.94	18120
				25.64	4.87
				40.07	12.62
				24.28	4.83
				35.21	9.06
				42.46	14.6
				32.07	8.54
				35.48	9.88
				45.65	15.82
				27.46	6.05
				59.78	24.58
				22.28	4.06
				91728.36	16330.87
				28.61	6.96
				37.26	8.73
cytoplasm;intracellular transport;nucleus;cytoplasm;centros				74.06	31.91
				21.41	3.9
				23.08	4.56
membrane;integral component of membrane;membrane;ir				36	78.83
extracellular	hsa:3976	hsa04060;hs	Cytokine-cytokine re	53.74	108.26
				25.78	5.93
				60.35	24.95
				14.09	0.77
				27.48	6.38
				24.34	5.38
membrane;integral component of membrane				51.73	17.61
				28.98	7.45
				29.22	7.39
				19.43	3.02

	hsa:79178	hsa00730;hs	Thiamine metabolism	50.53	19.98
				38.86	11.61
protein ubiquitination;intracellular signal transduction;cytc				60.69	22.54
				35.73	11.02
nucleus;endoplasmic reticulum;protein deubiquitination;ul				14.18	0.51
				52.66	21.07
				47.02	17.18
				37.41	9.36
				17	1.84
				51.68	20.18
				29.66	7.95
				19.81	3.11
				59.8	24.04
				50.27	17.41
				23.06	4.3
				22.02	4.36
				39.25	82.66
				27.36	6.49
				47.99	15.82
cytoplasm;extracellular region;extracellular negative regula				57.11	24.51
				32.58	8.21
				25.88	5.87
metal ion binding;endoplasmic reticulum;hydrolase activity				43.67	90.66
				21.02	3.51
growth facto	hsa:83729	hsa04060;hs	Cytokine-cytokine re	26.56	62.97
				22.34	4.39
				20.73	3.83
cytoplasm;calcium ion binding;cell division;extracellular reg				38.57	13
				48.25	17.71
				32.79	9.28
				30.38	8.12
				28.38	7.13
				22.55	4.37
				42.15	13.72
cytosol;nucl	hsa:79798	hsa04934	Cushing syndrome	41.06	84.88
				54.97	23.22
				40.29	12.21
protein binding;protein binding;protein binding;protein bi				32.15	8.65
				51.02	19.61
membrane;protein transport;Golgi apparatus;intracellular r				15.76	45.35
				21.17	4.09
nucleus;nucleoplasm;regulation of transcription, I	TF_bZIP			30.29	71.44
RNA bindin	hsa:8364	hsa05034;hs	Alcoholism ;Viral carc	232.26	61.53
membrane;integral component of membrane;endoplasmic				30.5	70.56
				29.55	7.47
				13.57	0.8
				20.73	3.66
cytoplasm;n	hsa:415117	hsa04130	SNARE interactions ir	32.35	8.83

				31.88	7.11
RNA bindin	hsa:23237	hsa05031	Amphetamine addict	4.32	25.17
membrane;i	hsa:391191	hsa04740	Olfactory transductio	13.76	0.98
				30.85	8.52
				20	3.36
				16.24	1.76
				21.66	4.39
				22.39	4.84
				19.22	2.5
				29.3	7.95
				32.37	9.19
membrane;i	hsa:5799	hsa04940	Type I diabetes mellit	17.01	47.9
				31.02	9.18
				19.85	3.51
				23.44	58.68
				54.24	22.62
				33.9	10.42
				13.55	0.71
				24.38	4.86
nucleus;membrane;plasma membrane;cell projection;cell ju				39.58	81.79
membrane;plasma membrane;plasma membrane;protein b				61.68	27.4
				29.29	7.92
				29.3	7.05
nucleus;nuc	hsa:1410	hsa04141;hs	Protein processing in	14.71	42.57
				17.25	2.37
cytoplasm;h	hsa:1852	hsa04010;hs	MAPK signaling path	14.7	43.23
				71.85	32.79
				29.05	7.67
				22.77	4.8
membrane;integral component of membrane;membrane;ir				33.07	9.45
				41.21	14.47
intracellular;intracellular protein transport;Rab GTPase binc				31.14	69.42
				16.46	2.03
				60.58	26.77
				8.08	0
metal ion binding;protein ubiquitination;protein binding;u				34.53	75.82
				31.76	9.55
membrane;integral component of membrane;G protein-co				24.39	5.89
				46.14	17.43
				49.51	20.27
oxidoreduct	hsa:7923	hsa00061;hs	Fatty acid biosynthes	32.79	71.11
				17.53	2.83
				18.13	3.1
				47.62	18.66
				48.11	18.53
				21.65	4.54
				29.61	7.87
				35.02	11.19

				35.63	11.11
				23.01	5.15
				69.07	33.05
membrane;integral component of membrane;protein bindi				46.78	94.21
				30.33	8.78
				33.14	71.65
				23.76	4.8
				53.9	21.99
				20.59	4.08
membrane;r	hsa:3687	hsa04610;hs	Complement and coa	15.57	43.37
				45.88	17.04
				23.98	5.6
				16.83	2.58
				12.76	0.8
				17.95	2.81
				17.52	2.74
				27.73	7.62
				37.32	12.25
				14.83	1.83
cytoplasm;membrane;nucleotide binding;ATP binding;tran				13.95	1.28
				21.05	3.94
nucleus;nucleus;cytoplasm;protein binding;multicellular or				33.89	71.03
				23.38	5.3
positive reg	hsa:28514	hsa01522;hs	Endocrine resistance	35.24	73.03
				14.29	1.21
cytoplasm;c	hsa:874	hsa00590;hs	Arachidonic acid met	25.51	60.29
				16.78	2.52
membrane;integral component of membrane;plasma mem				24.29	56.88
				13.99	1.54
				22.79	4.82
				37.54	13.13
membrane;i	hsa:10893	hsa04928	Parathyroid hormone	48.13	19.53
				7.01	0
				58.2	25.93
				13.36	1.27
				30.8	9.4
				17.69	2.09
				77.52	37.45
				50.61	19.58
				21.57	4.39
				20.86	4.59
				22.35	5.18
				22.67	5.42
				45.35	18.05
				24.7	6.32
				31.87	9.58
				16.87	2.83
				56.04	21.97

				13.45	38.82
				33.47	10.62
membrane;integral component of membrane;multicellular				70.83	33.52
nucleus;DN/	hsa:85235	hsa04217;hs	Necroptosis ;Alcoholi	27.14	6.01
				14.1	1.56
				31.72	10.02
				68.01	31.72
				40.91	15.05
				9.83	0.24
				18.83	3.54
				12.11	0.77
				6.72	0
membrane;i	hsa:51206	hsa04512;hs	ECM-receptor interac	42.92	15.65
membrane;integral component of membrane;calcium ion b				31.22	9.06
				22.17	5.12
				16.23	2.32
				13.97	1.28
				31.55	9.72
				60.4	27.58
				33.55	10.51
				15.3	2.27
				17.05	2.76
				32.14	10.32
				27.08	7.54
				47.51	19.99
membrane;r	hsa:29785	hsa00830;hs	Retinol metabolism ;I	8.99	31.02
				61.01	28.34
				18.95	3.78
membrane;i	hsa:94030	hsa04514	Cell adhesion molecu	30.34	66.47
				22.26	4.97
				41.61	14.5
				15.95	2.56
				11.03	36.62
				26.88	7.48
				20.46	4.05
				6.76	0
				50.63	21.76
				18.07	3.35
				31.29	66.52
				25.21	6.23
				22.91	5.68
				9.78	32.79
				14.51	2.03
cytoplasm;cytoskeleton;centrosome;microtubule organizin				19.86	3.83
				18.13	3.54
molecular_function;biological_process;cellular_component				62.72	30.33
				24.79	5.81
				35.43	10.75

					35.75	12.15
membrane;integral component of membrane;metal ion bir					38.51	14.57
					21.38	4.8
microtubule	hsa:147700	hsa05010;hs	Alzheimer disease ;Pa		23.14	53.65
					47.64	19.28
					27	7.46
					18.19	3.57
					40.75	14.83
					40.17	14.69
					15.74	2.3
					32.68	10.93
					25.69	6.73
cell adhesio	hsa:131873	hsa04151;hs	PI3K-Akt signaling pa		22.18	5.36
					19.43	3.93
					25.55	7.38
					41.96	16.59
lysosome;lysosome;lysosome;lysosome					29.21	9.26
					28.88	8.33
					29.14	8.88
					13.39	1.52
					22.07	5.62
RNA binding;RNA binding;mRNA transport;nucleus;nucleu					53.3	22.57
					39.08	13.6
					25.27	6.52
					25.86	6.42
					13.03	1.28
					4.5	21.77
					17.39	3.11
					33.72	11.71
					52.39	22.36
					24.91	5.96
nucleus;regulation of transcription, DNA-templat				Homeob	33.49	69.74
					16.18	2.82
methylated histone binding;methylated histone binding;m					25.85	4.82
					26.25	7.69
nucleus;zinc	hsa:2100	hsa01522;hs	Endocrine re	ESR-like	43.67	17.98
					17.57	3.38
					29.29	9.14
membrane;integral component of membrane;ion transport					48.13	20.55
					22.62	5.49
					17.12	3.13
actin binding;cell projection;cytoplasm;actin filament bindi					7.13	27.61
					53.58	23.4
					48.68	21.33
					29.73	9.44
					51.33	21.96
membrane;integral component of membrane;integral comp					31.81	10.33
					13.77	1.75

				38.3	13.8
				25.08	6.76
				18.08	3.4
				25.45	6.34
				46.61	19.1
				56.44	24.93
catalytic acti	hsa:81616	hsa00061;hs	Fatty acid biosynthes	61.5	29.54
				27.27	8.2
membrane;integral component of membrane				13.73	1.6
membrane;i	hsa:57835	hsa04976	Bile secretion	24.01	6.53
nucleus;nuc	hsa:8331	hsa04217;hs	Necroptosis ;Alcoholi	33.83	8.16
				29.88	9.41
				19.33	4.39
				42.94	17.37
				14.82	2.32
negative reg	hsa:5739	hsa04080;hs	Neuroactive ligand-r	34.75	70.62
				35.15	12.75
				22.23	5.35
				69.77	32.02
				10.79	0.51
				28.75	8.48
				19.86	4.39
				43.84	17.63
				11	0.53
				17.66	3.53
				20.3	4.45
				20.41	4.16
				15.77	2.79
				18.16	3.85
				23.75	6.4
				26.11	7.29
				12.33	1.28
				50.12	22.36
				22.8	5.98
				20.02	4.63
				48.01	20.77
				29.52	9.79
perinuclear region of cytoplasm;regulation of ventricular ca				22.57	52
				23.89	6.14
				32.3	10.78
				16.64	3.34
				37.27	14.4
				14.29	2
				16.88	3.28
nucleus;pro	hsa:55502	hsa05165	Human papi bHLH	21.79	50.02
				15.05	2.51
				13.31	1.82
				12.25	1.26

				36.11	14.05
intracellular	hsa:374875	hsa00140;hs	Steroid hormone bio:	16.93	43.13
				41.39	15.54
				11.79	1.02
				16.51	3.02
membrane;integral component of membrane				5.84	0
				57.4	26.95
				26.18	7.97
				18.79	3.86
				33.58	11.92
nucleus;DNA binding;chromatin DNA binding;nucleosome				13.04	1.77
growth facto	hsa:27006	hsa04010;hs	MAPK signaling path	19.04	4.36
				33.55	10.43
				19.17	4.06
				24.43	7.1
				18.61	3.8
				22.85	52.24
				44.24	18.92
				34.12	12.83
				13.9	2.08
				53.66	24.59
				16.78	3.26
				15.53	2.77
				26.09	7.95
				35.87	12.82
				40.47	14.89
				72.76	35.49
				11.71	1.04
				18.41	4
				15.16	2.81
				36.73	13.67
				19.53	4.62
				19.47	4.44
				38.77	13.84
				23.15	6.19
				33.98	12.34
				27.44	8.65
				13.24	1.5
				21.65	5.69
intracellular;nucleotide binding;protein kinase activity;protein serine/threonine kinase activity;					
				22.53	6.11
				22.58	6.08
cytoplasm;cytoplasm;mitochondrion;mitochondrion;autop				22.12	53.75
				10.32	0.53
				13.59	2.03

				29.34	9.84
				24.55	6.57
nucleus;nuc	hsa:85417	hsa04068;hs	FoxO signaling pathv	21.52	5.63
				19.78	4.37
				15.8	3.04
				10.39	0.74
				20.51	5.05
				12.3	1.32
				6.08	0
				53.98	25.63
membrane;integral component of membrane;transmembra				35.29	71.93
positive reg	hsa:54361	hsa04150;hs	mTOR signaling path	27.61	8.63
				15.44	2.74
				23.92	52.69
				16.1	3.03
				22.39	6.17
				29.12	9.72
				16.79	2.9
				19.2	4.37
				25.9	7.84
negative regulation of endothelial cell apoptotic process;re				56.6	26.18
				53.12	25.41
				31.86	11.23
				27.07	8.98
				1.71	14.54
				12.05	1.56
				25.12	7.7
membrane;(	hsa:51764	hsa04014;hs	Ras signaling pathwa	31.9	11.36
				74.48	37.14
				5.66	0
				8.95	29.51
				5.56	0
				10.65	0.74
nucleus;cytoplasm;intracellular transport;nucleus;cytoplasr				29.8	10.54
cytoplasm;cell redox homeostasis;cytoskeleton;nucleoside				34.95	13.73
				39.64	14
				19.57	4.9
				13.82	2.27
				6.3	23.75
				28.05	9.32
				24.62	7.41
				13.47	1.74
				11.04	1.07
				18.39	3.46
				36.36	14.59
				7758.48	637.02
				17.89	3.31
				18.55	4.07

	18.18	4.32
	20.49	5.41
	21.37	5.66
	16.1	2.7
RNA bindin hsa:121504 hsa05034;hs Alcoholism ;Viral carc	26.95	8.08
Z disc;cytoplasm;membrane;actin binding;plasma membrai	36.23	13.84
	14.92	2.79
	36.09	14.36
	18.46	4.14
	21.79	6.2
oxidoreduct hsa:1733 hsa04919 Thyroid hormone sig	46.29	19.09
	12.97	36.4
	17.23	3.03
	50.49	23
	46.9	18.45
	5.69	0
cytoplasm;cell adhesion;cytoskeleton;cell junction;focal adl	28.7	58.05
	20.13	4.66
	48.93	22.07
	14.61	2.55
membrane;i hsa:1141 hsa04080;hs Neuroactive ligand-r	18.46	4.13
positive regulation of cell proliferation;positive regulation	28.33	58.86
	20.97	5.65
	17.49	3.9
	28.36	9.09
	10.52	1.06
metal ion bi hsa:1300 hsa04974 Protein digestion anc	20.26	5.34
intracellular signal transduction;protein binding;microtubu	28.74	10.14
membrane;integral component of membrane;calcium ion b	23.67	6.96
	13.52	36.5
	16.82	3.83
	22.14	6.11
cytoplasm;protein binding;protein kinase binding;protein l	15.68	38.64
	30.56	11.17
	15.85	2.97
	11.37	1.27
	18.39	3.81
	13.82	2.32
	10.61	1.04
	16.92	3.85
	5.33	0
membrane;integral component of membrane;hydrolase ac	19.37	4.94
	23.95	7.43
	26.54	8.32
	19.61	4.86
	25.35	6.68
	26.37	8.79
	33.23	12.52

				15.83	2.99
hydrolase activity;phosphoprotein phosphatase activity;pe				47.06	21.3
				27.29	7.75
				24.38	7.6
				14.1	2.25
				18.49	4.57
				45.77	19.82
nucleus;cytoplasm;protein binding;perinuclear region of cy				22.78	6.66
				49.84	22.61
cytoplasm;membrane;integral component of membrane;ex				34.56	13.43
				14.14	2.58
				11.5	1.54
				14.61	2.81
				14.04	2.55
zinc ion binding;metal ion binding;hydrolase activity;pepti				33.8	13.09
				3.24	18.31
				17.68	3.67
				12.35	1.77
				5.29	0
				41.53	18.27
				9.19	0.53
				30.79	11.51
				29.21	10.66
				20.5	5.68
positive reg	hsa:5743	hsa00590;hs	Arachidonic acid met	21.86	54.11
				24.11	7.03
				52.79	25.76
nucleus;cytoplasm;membrane;mitochondrion;plasma mem				40.24	17.39
				15.58	2.97
				25.58	8.53
				13.87	2.26
				45.1	20.45
				26.82	8.43
				17.44	3.75
				9.8	0.74
				9.65	0.77
				25.02	8.12
				32.58	11.37
nucleus;nuc	hsa:8340	hsa05034;hs	Alcoholism ;Viral carc	35.15	12.12
				17.29	4.14
				26.67	8.54
				31.45	11.62
membrane;i	hsa:8325	hsa04150;hs	mTOR signaling path	28.93	59.16
				9.69	0.77
				18.31	3.89
nucleus;nucleus;cytoplasm;cytosol;nucleoplasm;cytoskelet				53.61	25.77
				42.54	16.32
membrane;integral component of membrane;plasma mem				15.85	41.9

					32.64	12.59
					31.26	11.9
					28.79	10.04
					35.29	13.69
extracellular space					24.41	7.82
					24.82	6.49
					28.14	9.93
membrane;i	hsa:623	hsa04020;hs	Calcium signaling pa		30.81	63.26
	hsa:5554	hsa04970	Salivary secretion		32.68	12.5
					26.8	9.27
negative regulation of phosphoprotein phosphatase activi					30.28	62.73
					14.19	2.83
					1.76	13.73
					12.1	1.82
					43.01	19.57
					15.61	2.7
positive reg	hsa:50943	hsa04659;hs	Th17 cell dif	Fork_hea	33.51	13.59
					13.86	2.61
					41.01	17.63
					25.58	8.06
					20.64	5.59
					27.74	9.47
					30.45	10.27
					24.1	7.84
					19.71	5.59
					11.37	1.48
					28.91	10.66
					15.2	3.34
					57.21	28.24
					31.59	11.65
					11.82	1.58
					11.36	1.47
					14.89	2.59
nucleic acid	hsa:155054	hsa05168	Herpes simp	zf-C2H2	15.08	37.49
RNA binding;membrane;integral component of membrane					32.27	10.96
hydrolase activity;hydrolase activity;hydrolase activity;hydr					28.69	10.14
					31.97	11.8
					9.01	0.5
					5.14	0
					29.36	10.44
					11.1	1.3
					12.18	1.84
					16.79	4.07
membrane;integral component of membrane;CatSper com					14.19	2.61
					20.75	5.79
					15.09	3.09
					24.13	7.66
					25.27	8.43

				25.83	8.59
				45.48	18.17
				18.64	4.81
				4.91	0
				9.87	1.06
				29.06	10.49
				14.54	3.05
				12.06	2.03
				15.23	2.53
				12.53	2.03
				23.31	6.34
				27.75	9.71
				3869.54	1846.77
				8.44	0.51
membrane;integral component of membrane;calcium ion b				13.25	2.53
				26.43	8.85
				8.58	0.5
				22.29	7.16
				10.1	1.27
				48.95	23.27
cytoplasm;nucleus;nucleus;cytoplasm;cytosol;growth facto				5.83	21.67
				1.62	13.19
				15.44	3.54
				24.07	7.72
				15.74	3.6
				44.78	19.23
				15.91	3.61
				22.89	7.22
				10.65	1.27
				9.64	0.98
				26.05	9.21
				19.66	5.42
				17.65	3.6
				10.71	1.5
				23.59	7.48
				15.68	3.61
				23.85	7.87
				15.02	3.25
				19.74	5.69
perinuclear	hsa:5350	hsa04020;hs	Calcium signaling pa	12.16	1.79
zinc ion binding;metal ion binding				11.24	1.56
				13.87	2.83
				35.13	14.92
				32.4	12.5
				8.3	0.51
				13.9	34.79
				14.1	2.61
				10.02	1.27

membrane;i	hsa:51554	hsa04060;hs	Cytokine-cytokine re	36.6	15.59
				8.77	0.77
membrane;extracellular region;endoplasmic reticulum				17.15	3.69
				13.99	2.76
				24.52	8.45
				10.91	1.22
				27.26	10.11
				18.7	4.22
				17.33	4.6
nucleus;nuc	hsa:8338	hsa04217;hs	Necroptosis ;Alcoholi	27.93	9.24
				27.16	10.07
				24.54	8.22
				14.32	3.05
membrane;i	hsa:3595	hsa04060;hs	Cytokine-cytokine re	33.81	14.12
				4.92	0
				34.6	14.1
extracellular space;hydrolase activity;peptidase activity;pro				27.25	10.25
				8.67	0.77
cytoplasm;membrane;apoptotic process;cytoplasm;membra				23.19	51.95
				9.36	1.03
				35.88	15.77
				15.72	3.84
membrane;integral component of membrane;membrane;ir				33.93	14.33
				14.97	3.54
				18.44	4.26
				10.05	1.27
membrane;integral component of membrane;symporter ac				17.04	4.38
cytoplasm;membrane;protein transport;nucleotide binding				23.17	48.44
				12.58	2.1
				21.02	6.41
				39.33	15.51
				8.81	0.79
				18.17	5.12
				15.83	3.63
				29.15	10.84
				10.24	1.22
				4.83	0
				17.88	4.89
membrane;i	hsa:6751	hsa04024;hs	cAMP signaling pathw	5.37	20.33
				12.19	2.32
				30.17	11.1
				12.9	2.35
				4.59	0
				4.59	0
				36.69	15.79
				11.54	1.73
				23.46	7.46
				20.05	5.59

					11.72	2.03
					14.84	3.54
					16.51	4.34
					9.14	1.04
nucleus;metal ion binding;regulation of transcript				Fork_hea	42.52	19.36
					15.08	3.52
					6.4	0.24
					12.15	2.3
mRNA bindi	hsa:406925	hsa05206	MicroRNAs in cancer		9.74	1.27
membrane;integral component of membrane;membrane;ir					15.66	3.8
					8.48	0.74
					21.4	6.33
					9.03	1.03
					11.4	2.04
intracellular protein transport;vesicle-mediated transport;n					28.97	10.58
					24.72	8.71
					13.31	2.75
					8.17	0.51
membrane;plasma membrane;cell junction;synapse;postsy					17.98	40.92
					46.7	22.88
					36.14	15.4
					13.77	2.89
membrane;integral component of membrane;membrane;ir					24.96	8.88
					10.21	1.21
					21.61	7.1
					10.98	1.77
					29.16	10.23
					21.93	7.37
					6.35	0.27
nucleus;nucleus;metal ion binding;nucleoplasm;transferase					44.63	20.73
regulation c	hsa:51738	hsa04024;hs	cAMP signaling pathv		40.98	18.34
					10.49	1.53
membrane;i	hsa:3820	hsa05144	Malaria		39.63	18.56
					18.84	4.94
					34.17	14.49
					30.6	12.69
membrane;i	hsa:59285	hsa04010;hs	MAPK signaling path		8.98	26.97
					18.76	5.62
					8.39	0.77
					10.65	1.51
					14.39	3.05
					7.69	0.5
cytoplasm;Golgi apparatus;cytosol;actin binding;actin filam					27.74	10.52
					27.4	10.06
circadian re	hsa:8863	hsa04710;hs	Circadian rhythm ;Cir		42.78	20.87
					6.26	0.27
					11.09	1.74
					10.67	1.53

					11.22	1.73
					11.61	1.79
					17.68	4.69
					17.86	4.22
					44.16	20.68
					30.78	12.65
					27.08	9.64
					20.45	6.69
					15.35	3.17
					14.81	3.14
					16.46	4.57
					21.76	7.09
					10.84	1.82
					15.74	3.5
					17.02	4.67
					9.78	1.25
					12.29	2.31
					28.06	10.67
					10.85	1.82
					17.67	4.82
					20.9	6.96
membrane;i	hsa:1133	hsa04020;hs	Calcium signaling pa		8.11	0.77
					22.98	7.63
					11.86	2.32
nucleus;nuc	hsa:4929	hsa04925;hs	Aldosterone	NGFIB-li	18.63	40.72
protein binding;extracellular region;extracellular space;corr					0.37	8.56
					16.33	4.14
					21.01	6.92
					16.23	4.38
membrane;integral component of membrane;metal ion bir					20.27	5.97
					18.7	5.39
					13.45	2.79
					32.07	13.64
					9.09	1.04
					38.65	16.58
					10.34	1.55
					21.64	6.77
					17.66	4.92
					9.14	1.07
					21.88	7.47
					7.64	0.51
					42.15	19.3
					32.45	13.79
					29.61	12.2
					17.22	4.43
					4.4	0
					8.21	0.79
intracellular;membrane;nucleotide binding;protein ubiquit					40.34	19.39

				20.56	6.17
				25.17	9.03
membrane;integral component of membrane;G protein-co				27.1	9.52
				18.84	5.39
				10.57	1.74
				23.42	7.31
				19.66	6.18
				4.27	0
				16.41	4.58
carbohydrate binding;protein binding;galactoside binding				10.22	1.32
				17.32	4.58
				26.23	9.69
				18.57	5.62
				14.65	3.61
				7.46	0.5
				19.58	5.83
				30.16	12.48
				9.5	1.26
protein kina	hsa:1027235	hsa04114;hs	Oocyte meiosis ;Prog	21.18	6.78
				14.01	3.25
cytoplasm;n	hsa:4145	hsa04722	Neurotrophin signali	2.6	13.44
				14.06	3.31
				24.71	7.53
membrane;integral component of membrane;cytosol;nucle				4.46	0
				6.74	0.26
				9.72	1.3
cytoplasm;membrane;cytosol;regulation of apoptotic proc				20.87	6.76
protein homooligomerization;protein binding				12.2	2.57
				15.58	4.01
				16.47	4.32
				25.31	8.68
				28.7	11.57
				12.88	2.61
				26.58	10.03
				14.93	3.77
				12.18	2.52
				38.64	17.98
				10.23	0.95
				5.84	0.27
				14.6	3.43
nucleus;regulation of transcription, DNA-templat			Homeob	0	5.37
				26.86	10.27
				8.44	1.03
hydrolase ac	hsa:834	hsa04217;hs	Necroptosis ;NOD-lik	8.06	24.57
membrane;integral component of membrane;hydrolase ac				28.27	11.49
				14.9	3.61
				18.38	5.18
				17.35	4.36

				10.16	1.55
negative regulation of fibroblast growth factor receptor sig				12.66	2.46
				13.12	3.05
				8.94	1.07
				17.68	5.06
				15.13	3.79
				4.26	0
				16.7	3.72
membrane;integral component of membrane;calcium ion b				25.05	8.79
				8.82	1.02
				16.69	4.86
				13.94	3.29
				31.49	12.51
				8.06	0.79
				9.7	1.31
				12.99	2.82
				12.19	2.55
				10.38	1.58
				20.53	7.13
				35.98	16.51
				12.62	2.55
				21.87	7.85
				8.46	0.77
metal ion binding;snoRNA splicing;U2-type spliceosomal c				11.48	2.27
				6.02	0.26
				36.18	16.18
				9.28	0.77
				8.39	0.71
				9.19	1.07
				13.28	2.73
membrane;integral component of membrane;carbohydrate				0	5.35
				25.62	9.96
cytoplasm;n	hsa:4671	hsa04621;hs	NOD-like receptor si	34.79	15.83
				36.05	15.75
				13.03	3.07
				8.34	1.04
membrane;zinc ion binding;metal ion binding;intracellular;				5.83	0.26
				9.59	1.54
				19.34	6.37
				6.16	0.27
				27.58	9.9
				12.67	2.84
protein binding				22.58	7.57
membrane;integral component of membrane;G protein-co				11.72	2.34
				30.66	13.29
				8.07	0.78
				10.57	1.71
membrane;(	hsa:2785	hsa04014;hs	Ras signaling pathwa	31.06	13.7

				17.26	4.93
				5.8	0.26
				19.61	6.67
				11.59	2.32
membrane;integral component of membrane;membrane;ir				12.06	2.52
				5.71	0.27
				5.75	0.26
				20.71	6.2
				47.28	20.63
cytoplasm;re	hsa:398	hsa04722;hs	Neurotrophin signali	19.09	41.38
				7.64	0.77
				39.54	19.48
membrane;integral component of membrane;Golgi membr				23.83	8.91
				10.93	2.06
				32.31	14.21
				5.05	18.37
				28.09	11.65
				10.14	1.77
				21.96	7.08
				11.41	1.97
				7.23	0.52
				8.9	1.29
				7.7	0.74
protein dim	hsa:83547	hsa04145;hs	Phagosome ;Salmone	13.29	31.7
				21.27	7.69
				15.03	4.05
				12.11	2.38
				23.67	8.04
				22.54	8.12
				11.68	2.26
				9.37	1.33
				11.63	2.56
				14.06	3.57
				25.56	9.63
				15.13	3.83
				11.41	2.31
				4.04	0
				16.28	4.59
				27.49	10.7
				4.16	0
				6.96	0.5
				19.25	6.16
				15.49	4.12
				16.7	4.83
				13.45	3.34
				28.93	11.47
				18.64	5.71
				25	9.4

				9.31	1.53
				19.99	6.96
				17.6	5.21
				8.9	1.25
				5.69	0.27
membrane;i	hsa:4360	hsa04145;hs	Phagosome ;Tubercu	3.99	0
cytoplasmic vesicle;acrosomal vesicle;multicellular organisr				18.49	6.09
				15.75	4.63
				10.53	2.01
membrane;integral component of membrane;calcium ion b				8.66	1.27
				11.72	2.49
				15.69	4.35
				19.86	6.66
				29.85	12.11
				21.64	7.81
				20.07	6.98
				3.98	0
				15.33	3.6
				16.08	4.47
membrane;integral component of membrane;integral comp				13.02	3.12
				8.62	1.27
protein binding;extracellular region;collagen trimer;molecu				28.86	59.53
				4.37	0
				8.21	1.06
				14.94	4.13
				11.94	2.3
				22.71	8.72
				25.26	6.17
				14.15	3.64
				15.88	4.32
extracellular region;proteolysis;peptidase activity;extracellu				16.32	4.8
membrane;i	hsa:6519	hsa04974	Protein digestion and	14.06	3.21
				24.52	9.55
intracellular;	hsa:10663	hsa04060;hs	Cytokine-cytokine re	22.83	8.36
				16.54	4.09
				12.28	2.73
				9.73	1.79
RNA bindin	hsa:8360	hsa05034;hs	Alcoholism ;Viral carc	13.87	3.55
RNA bindin	hsa:8359	hsa05034;hs	Alcoholism ;Viral carc	14.11	3.57
				12.89	3.03
nucleus;nuc	hsa:25788	hsa03440	Homologous recomb	7.92	1.04
				13.68	31.21
				31.26	13.96
				13.83	3.22
				24.36	9.16
				10.38	2.04
				9.52	1.32
				15.79	4.42

				19.75	6.89
				5.38	0.26
				29.85	12.39
				8.06	1.04
membrane;integral component of membrane;protein bindi				6.91	0.52
membrane;i	hsa:390037	hsa04740	Olfactory transductio	5.42	0.26
RNA binding;nucleus;nucleus;cytoplasm;cytoplasm;transfe				1.3	10.08
				14.22	3.64
				3.85	0
				14.05	3.84
				11.7	2.35
				29.82	13.17
				7.46	0.8
				6.96	0.54
membrane;integral component of membrane;calcium ion b				10.34	2.03
				13.35	3.31
				15.31	4.13
				30.29	13.26
				5.66	0.26
				35.34	16.67
				10.1	1.79
				8.11	1.06
				7.88	0.77
				17.58	5.63
				10.85	2.31
				12.06	2.61
folic acid bir	hsa:2352	hsa01523;hs	Antifolate resistance	5	17.64
				3.95	0
				21.09	7.85
				24.11	8.89
				3.8	0
membrane;i	hsa:390649	hsa04740	Olfactory transductio	9.4	1.54
				9.49	25.6
				19.54	6.59
				14.79	3.78
				8.52	1.27
				7.85	0.77
				19.13	5.89
				9.03	1.53
				29.38	12.99
cytoplasm;membrane;plasma membrane;cytoskeleton;cell				13.5	32.37
				10.26	1.79
membrane;i	hsa:50831	hsa04742	Taste transduction	14.37	4.02
				5.57	0.24
intracellular;membrane;nucleotide binding;signal transduc				6.36	19.52
				36	17.21
membrane;integral component of membrane;membrane;ir				27.15	11.68
				10.29	2.07

				28.55	11.45
				15.64	4.39
				10.82	2.02
cytoplasm;c	hsa:10993	hsa00260;hs	Glycine, serine and th	24.79	8.57
				8.95	1.53
				8.12	1.07
				9.59	1.58
				0	4.81
RNA binding	hsa:8361	hsa05034;hs	Alcoholism ;Viral carc	19.49	6.64
				33.51	15.83
membrane;nucleus;nucleoplasm;mitochondrion;plasma me				12.49	2.59
				37.95	18.33
				13.81	3.78
				17.93	5.94
				26.9	10.93
				17.69	5.29
				37.75	18.34
				17.01	5
				16.88	5.41
				5.39	0.26
				21.63	7.86
				9.51	1.59
				5.24	0.26
				9.36	1.55
positive reg	hsa:3918	hsa04151;hs	PI3K-Akt signaling pa	19.24	39.56
				19.01	6.47
				8.96	1.33
				18.99	6.01
				6.68	0.51
				15.77	4.83
				18.05	5.85
				20.08	7.18
metal ion binding;transferase activity;protein ubiquitinatio				19.37	6.93
intracellular	hsa:1285	hsa04151;hs	PI3K-Akt signaling pa	28.18	12.48
				17.55	5.29
				19.03	6.17
				13.44	3.39
				7.69	1.04
				11.11	2.31
				11.05	2.31
				9.69	1.83
				18.88	6.65
				3.89	0
				10.99	2.12
				22.59	8.1
nucleus;cytosol;nucleoplasm;cell cycle;gamete generation;r				15.35	4.01
				25.57	10.09
				5.25	0.27

				12.01	2.76
				3.71	0
				17.45	5.21
				17.75	5.64
				9.28	1.54
				11.77	28.9
				13.01	2.85
				20.91	7.95
				8.59	0.98
protein serine/threonine kinase activity;ATP binding;protei				35.86	17.6
				21.96	8.5
				8.54	1.27
				36.42	16.76
				21.68	8
				30.32	14.05
				8.44	1.25
				18.23	6.18
				17.74	38.01
metal ion bi	hsa:91734	hsa00900;hs	Terpenoid backbone	14.24	3.67
				26.02	10.84
nucleus;nucleus;cytoplasm;protein binding;biological_proc				0.42	6.84
				9.37	1.5
				8.06	1.02
				24.85	9.89
				12.74	2.96
				6.35	0.5
				16.89	5.36
				15.52	4.54
				25.85	10.98
				12.3	3.04
membrane;z	hsa:4326	hsa04928	Parathyroid hormone	13.95	32.1
				28.14	11.98
cytoplasm;c	hsa:58538	hsa04530	Tight junction	15.78	33.86
				20.95	7.95
				10.9	2.35
				9.35	1.55
				7.84	1.01
				6.35	0.53
				3.7	0
protein binding;protein binding;protein binding				9.14	1.57
				104.41	289.79
				9.85	1.48
				19.4	5.97
				3.66	0
				29.51	12.6
				18.5	6.46
				17.16	5.41
				21.46	7.53

extracellular space	5.01	0.26
	8.44	1.21
	16.1	4.89
	9.56	1.82
	17.21	5.61
	8.75	1.5
	10.38	2.3
	23.94	9.64
	10.87	2.05
	21.85	8.35
cytoplasm;cytosol;nucleoplasm;protein binding;guanyl-nu	9	1.26
	9.66	1.57
	13.94	3.5
	25.63	11.07
	7.32	0.8
	7.88	1.06
	19.2	6.88
cytoplasm;intracellular protein transport;metal ion binding	8.8	1.53
	10.77	2.27
	7.84	21.3

on channel activity;ion transport;voltage-gated ion channel activity;regulation of ion tr

;ATP binding;protein phosphorylation;transferase activity;MAP kinase activity;kinase act

ansmembrane transport;plasma membrane;plasma membrane;calcium ion transport;calc

:ivity;MAPK cascade;phosphorylation;intracellular;nucleotide binding;protein kinase acti

cium ion transmembrane transport;voltage-gated calcium channel activity;calcium chanr

vity;protein serine/threonine kinase activity;ATP binding;protein phosphorylation;trans

nel activity;calcium ion transmembrane transport;voltage-gated calcium channel comple

ferase activity;MAP kinase activity;kinase activity;MAPK cascade;phosphorylation;intrace

ex;low voltage-gated calcium channel activity;calcium ion import;plasma membrane;volta

ellular;nucleotide binding;protein kinase activity;protein serine/threonine kinase activity;

age-gated calcium channel complex;regulation of membrane potential;calcium ion impc

ATP binding;protein phosphorylation;transferase activity;MAP kinase activity;kinase act

ort;scaffold protein binding;calcium ion import;low voltage-gated calcium channel activit

ivity;MAPK cascade;phosphorylation;nucleus;cytoplasm;cytoplasm;membrane;nucleotid

ty;low voltage-gated calcium channel activity;voltage-gated calcium channel activity inv

e binding;protein kinase activity;protein serine/threonine kinase activity;ATP binding;pr

volved in AV node cell action potential;voltage-gated calcium channel activity involved S

rotein phosphorylation;transferase activity;MAP kinase activity;kinase activity;phosphory

SA node cell action potential;neuronal action potential;positive regulation of calcium ior

ylation;mitochondrion;mitochondrion;plasma membrane;protein binding;rhythmic proc

ı-dependent exocytosis;cardiac muscle cell action potential involved in contraction;

ess;Fc-epsilon receptor signaling pathway;nucleus;cytoplasm;i
