# Supplementary material for: Downregulated PDIA3P1 lncRNA Impairs Trophoblast Phenotype by Regulating Snail and SFRP1 in PE
Source: Anal Cell Pathol (Amst). 2024 Apr 27;2024:8972022. doi: 10.1155/2024/8972022 (PMC11074859; doi:10.1155/2024/8972022)
Supplement: Supplementary 5 — Detailed process of RNA pulldown assays. [file 8972022.f5.docx]

**RNA pulldown assay**

**1.** **Plasmid templates Linearization**

**Prepare reagent supplies：**Sense RNA Plasmid、Antisense RNA Plasmid、Restriction Enzyme Digestion、Ultrapure water、Buffer

1. Thaw the frozen reagents

2. Assemble transcription reaction at room temp

| **Component** | **Amount** |
| --- | --- |
| Ultrapure water | to 50 µL |
| Buffer | 5 µL |
| Plasmid | 1 µg |
| Restriction Enzyme Digestion | 1 µL |

3. Mix thoroughly，Incubate at 37°C, 2 hr

4. Agarose gel electrophoresis confirmed adequate digestion

5. Expand the optimized reaction system

6. Heat inactivation 65°C for 20 min

7. Proteinase K、0.5% SDS for 30 min at 50°C，follow this with phenol/chloroform extraction (using an equal volume).

8. 2 volumes of ethanol precipitation. Mix well and chill at –20°C for at least 15 min.

9. Then pellet the DNA for 15 min in a microcentrifuge at top speed. Remove the supernatant, Resuspend in dH2O or TE buffer at a concentration of 0.5–1 µg/µL.

**2. RNA Transcription in Vitro**

**Prepare reagent supplies：**mMESSAGE mMACHINE® Kit AM1344、DNA template、EP tube

1. Thaw the frozen reagents

Place the RNA Polymerase Enzyme Mix on ice, it is stored in glycerol and will not

be frozen at –20°C.

Vortex the 10X Reaction Buffer and the 2X NTP/CAP until they are completely in solution. Once thawed, store the ribonucleotides (2X NTP/CAP) on ice, but keep the 10X Reaction Buffer at room temperature while assembling the reaction.

All reagents should be microfuged briefly before opening to prevent loss and/or contamination of material that may be present around the rim of the tube.

2. Assemble transcription reaction at room temp

The following amounts are for a single 20 µL reaction. Reactions may be scaled up

or down if desired

| **Component** | **Amount** |
| --- | --- |
| Nuclease-free Water | to 20 µL |
| 2X NTP/CAP | 10 µL |
| 10X Reaction Buffer | 2 µL |
| (optional) [α-32P]UTP as a tracer  linear template DNA† | (1 µL)  0.1–1 µg |
| Enzyme Mix | 2 µL |

3. Mix thoroughly

Gently flick the tube or pipette the mixture up and down gently , and then microfuge tube briefly to collect the reaction mixture at the bottom of the tube.

4. Incubate at 37°C, 2 hr

Typically，80% yield is achieved after a 1 hr incubation. For maximum yield, we recommend a 2 hr incubation. Since SP6 reactions are somewhat slower than T3 and T7 reactions, they especially may benefit from the second hour of incubation.

5. (optional) Add 1 µL TURBO DNase, mix well and incubate 15 min at 37°C

This DNase treatment removes the template DNA. For many applications it may not be necessary because the template DNA will be present at a very low concentration relative to the RNA.

1. Add 1 µL TURBO DNase, and mix well.
2. Incubate at 37°C for 15 min.

**3. Purification for RNA Transcription Reactions**

**Prepare reagent supplies：**MEGAclear™ Kit AM1908

1. Bring the RNA sample to 100 µL with Elution Solution. Mix gently but thoroughly

2. Add 350 µL of Binding Solution Concentrate to the sample. Mix gently by pipetting.

3. Add 250 µL of 100% ethanol to the sample. Mix gently by pipetting.

4. Pipet the RNA mixture onto the Filter Cartridge. Centrifuge for ~15 sec to 1 min, or until

the mixture has passed through the filter. Centrifuge at RCF 10,000–15,000 × g (typically 10,000–14,000 rpm).

5. Wash with 2 × 500 µL Wash Solution.

6. Elute RNA from the filter with 50 µL Elution Solution using one of the methods

described below; they are equivalent in terms of RNA recovery.

a. Pre-heat 110 µL of Elution Solution per sample to 95° C.

b. Apply 50 µL of the pre-heated Elution Solution to the center of the Filter Cartridge, close the cap of the tube and centrifuge for 1 min at room temperature (RCF 10,000–15,000 x g) to elute the RNA.

c. To maximize RNA recovery , repeat this elution procedure with a second pre-

heated 50 µL aliquot of Elution Solution. Collect the eluate into the same

Collection/Elution Tube.

7. (optional) Precipitate with 5 M Ammonium Acetate. To concentrate the RNA,

precipitate as follows:

a. Add 1:10 volume of 5 M Ammonium Acetate (NH4Ac) to the purified RNA.

Note: If the sample was eluted with 100 µL Elution Solution as suggested,

this will be 10 µL of 5 M NH4Ac.

b. Add 2.5 volumes of 100% ethanol (275 µL if the RNA was eluted in 100 µL).

Mix well and incubate at –20°C for 30 min.

c. Microcentrifuge at top speed for 15 min at 4°C or room temperature (RT).

d. Carefully remove and discard the supernatant.

e. Wash the pellet with 500 µL 70% cold ethanol, centrifuge again and remove

the 70% ethanol.

f. To remove the last traces of ethanol, quickly re-spin the tube, and aspirate any residual fluid with a very fine tipped pipette, or with a syringe needle.

g. Air dry the pellet. Resuspend the pellet using the desired solution and volume.

**4. Pierce RNA 3' End Desthiobiotinylation**

**Prepare reagent supplies：**Pierce RNA 3' End Desthiobiotinylation Kit、1-50pmol of RNA for labeling、Heated mixer/chiller for incubation at 37°C/16°C、Chloroform:isoamyl alcohol (24:1)、Nuclease-free pipette tips and tubes、5M NaCl、Ultrapure water、100% ethanol, ice-cold、70% ethanol, ice-cold

1. Thaw all kit components except the PEG 30% and DMSO on ice. Thaw DMSO at room temperature and warm the PEG 30% at 37°C for 5-10 minutes until volume is fluid.

2. Adjust the heating block to 85°C.

3. Transfer 5µL of the Non-labeled RNA Control to a microcentrifuge tube. Heat the RNA for 3-5 minutes at 85°C. Place RNA immediately on ice.

**Note:** The RNA may require heating to relax the secondary structure. Also, heating the RNA in the presence of ~25% DMSO may increase efficiency for RNA with significant secondary structure.

4. Prepare the labeling reaction for the control system or test RNA by adding components in the order listed in Table 1.

**Note:** The last added reagent is PEG 30%. Carefully pipette the PEG 30% into the reaction

mixture. Use a new pipette tip to mix the ligation reaction after the PEG 30% addition.

| **Component** | **Volume (µL)** | **Final Concentration** |
| --- | --- | --- |
| Nuclease-free Water | 3 | --- |
| 10X RNA Ligase Reaction Buffer | 3 | 1X |
| RNase Inhibitor | 1 | 40U |
| Non-labeled RNA Control or Test RNA | 5 | 50pmol |
| Biotinylated Cytidine Bisphosphate | 1 | 1nmol |
| T4 RNA Ligase | 2 | 40U |
| PEG 30% | 15 | 15% |
| **Total** | **30** | --- |

5. Incubate the reactions at 16°C for 2 hours for the control RNA. Ligation may require overnight incubation to increase efficiency.

6. Add 70µL of nuclease-free water to the ligation reaction.

7. Add 100μL of chloroform:isoamyl alcohol to each reaction to extract the RNA ligase. Vortex the mixture briefly, then centrifuge 2-3 minutes at high speed in a microcentrifuge to separate the phases. Carefully remove the top (aqueous) phase and transfer to a nuclease-free tube.

8. Add 10μL of 5M NaCl, 1µL of glycogen and 300μL of ice-cold 100% ethanol. Precipitate for ≥ 1 hour at -20°C.

9. Centrifuge at ≥ 13,000 × g for 15 minutes at 4°C. Carefully remove the supernatant, taking care not to disturb the pellet.

10. Wash the pellet with 300µL of ice-cold 70% ethanol. Carefully remove ethanol and air-dry the pellet (~5 minutes).

11. Resuspend the pellet in 20µL of nuclease-free water or buffer of choice.

**5. Pierce Magnetic RNA-Protein Pull-Down**

**Prepare reagent supplies：**Pierce Magnetic RNA-Protein Pull-Down Kit、Target RNA for labeling、Chloroform:isoamyl alcohol (24:1)、Ethanol, absolute、Cell Lysis Buffer (for preparation of cell lysate)、Magnetic separation stand

**A. Pre-Washing Streptavidin Magnetic Beads (Optional)**

1. Resuspend the beads in the original vial by gentle swirling or rotation.

2. Remove the amount to be treated and transfer to a nuclease-free tube.

3. Place tube on a magnetic stand to collect the beads against the sides of the tube.

4. Wash the beads twice with a 2X volume of 0.1M NaOH, 50mM NaCl (nuclease-free).

5. Wash the beads once in 100mM NaCl.

6. Continue with equilibration of magnetic beads for RNA capture (Section D).

**B. Preparation of Cell Lysate**

1.Cell lysates may be prepared using standard lysis buffers

2. Ensure the cell lysate protein concentration is greater than 2mg/mL, such that there is significant dilution into the Binding Reaction Buffer.

**C. Binding of Labeled RNA to Streptavidin Magnetic Beads**

**Note:** Use a range of 25-100pmol of RNA per 20-50µL of magnetic beads. The instructions below use a scale of 50pmol of RNA to 50µL of beads.

1. Add 50µL of streptavidin magnetic beads to a 1.5mL microcentrifuge tube.

2. Place the tube into a magnetic stand to collect the beads against the side of the tube. Remove and discard the supernatant.

3. Wash with an equal volume of 20mM Tris (pH 7.5). Resuspend beads by pipetting or

vortexing.

4. Repeat Steps 2 and 3.

5. Place the tube into a magnetic stand to collect the beads against the side of the tube. Remove and discard the supernatant.

6. Add an equal volume of 1X RNA Capture Buffer. Resuspend beads by pipetting or vortexing.

7. Add 50pmol of labeled RNA to the beads. Mix gently by pipetting.

8. Incubate for 15-30 minutes at room temperature with agitation.

**D. Binding of RNA-Binding Proteins to RNA**

1. Place the tube into a magnetic stand to collect the beads against the side of the tube. Remove and discard the supernatant.

2. Wash with an equal volume of 20mM Tris (pH 7.5). Resuspend beads by pipetting or vortexing.

3. Repeat Steps 1 and 2.

4. Place the tube into a magnetic stand to collect the beads against the side of the tube. Remove and discard the supernatant.

5. Dilute 10X Protein-RNA Binding Buffer to 1X

(i.e., 10µL into 90µL of ultrapure water for each reaction).

6. Add 100µL of 1X Protein-RNA Binding Buffer to the beads and mix well.

7. Prepare a Master Mix of RNA-Protein Binding Reaction (Table 2).

| **Reagent** | **Volume (µL) per 100µL reaction for control** | **Range** |
| --- | --- | --- |
| 10X Protein-RNA Binding Buffer | 10 | 5-20µL |
| 50% glycerol | 30 | 0-50µL |
| Lysate (protein conc. > 2mg/mL | 1-30 | 20-200µg |
| Nuclease-free water | to 100 | to 100µL |

8. Place the tube into a magnetic stand to collect the beads against the side of the tube. Remove and discard the supernatant.

9. Add 100µL of Master Mix to the RNA-bound beads. Mix by pipetting or gentle vortexing.

10. Incubate 30-60 minutes at 4°C with agitation or rotation.

**E. Washing and Elution of RNA-Binding Protein Complexes**

1. Place the tube into a magnetic stand to collect the beads against the side of the tube. Transfer the supernatant to a tube for later analysis.

2. Wash with equal volume of 1X wash buffer (100µL).

3. Repeat Steps 1 and 2 two additional times. Save wash supernatants for analysis, if desired.

4. Place the tube into a magnetic stand to collect the beads against the side of the tube. Transfer the supernatant to a tube for later analysis.

5. Add 50µL of Elution Buffer to the beads and mix well by vortexing. Incubate 15-30 minutes at 37°C with agitation.

6. Place the tube into a magnetic stand to collect the beads against the side of the tube.

7. Remove supernatant for downstream analysis.

8. If the downstream application is Western blotting, add reducing sample buffer to samples to 1X.

**F. Western Blot Analysis**
