# Supplementary figures and images for: Downregulated PDIA3P1 lncRNA Impairs Trophoblast Phenotype by Regulating Snail and SFRP1 in PE
Source: Anal Cell Pathol (Amst). 2024 Apr 27;2024:8972022. doi: 10.1155/2024/8972022 (PMC11074859; doi:10.1155/2024/8972022)

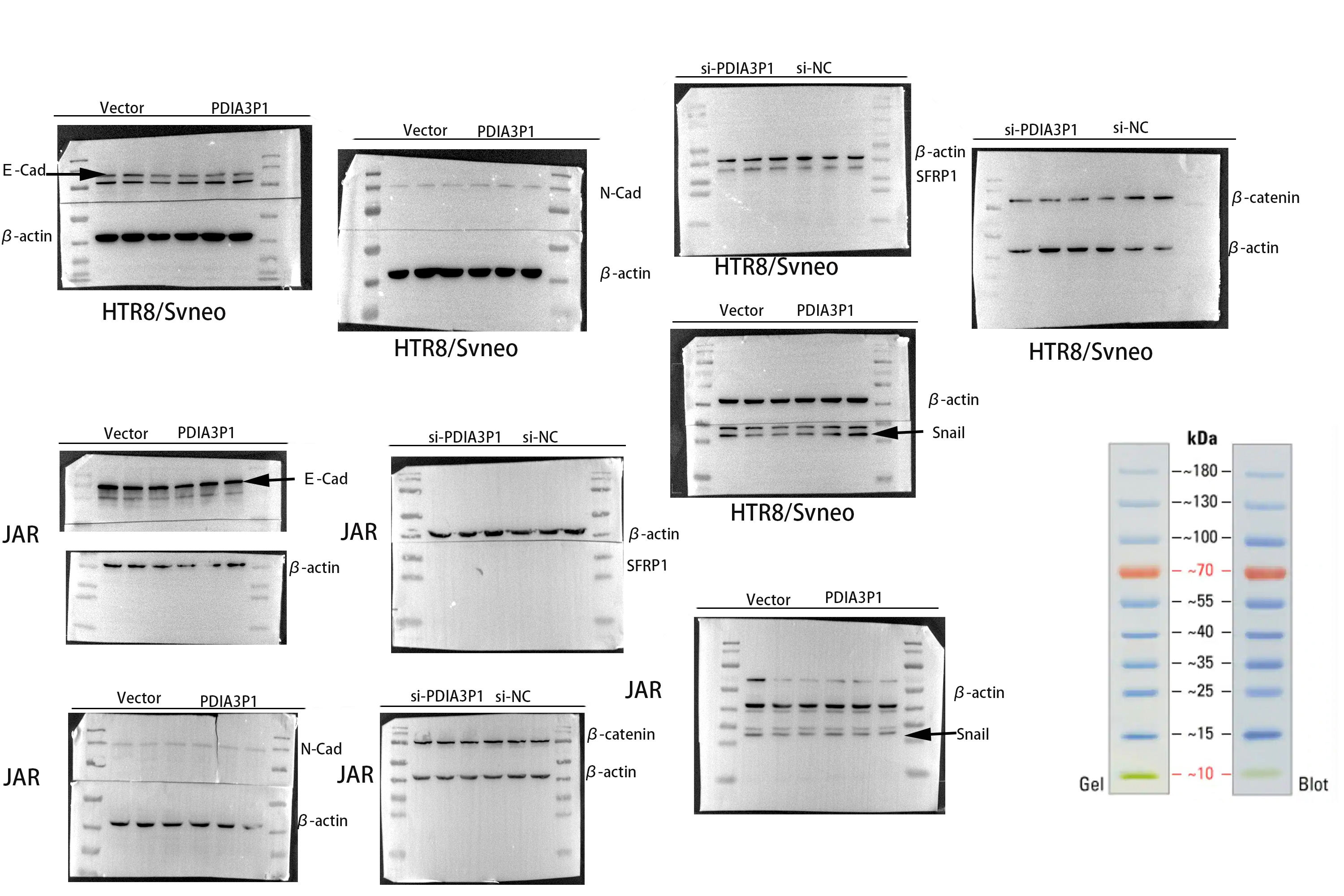

Supplement: Supplementary 6 — Uncut western gel imagines. [file 8972022.f6.jpg]
