# Supplementary material for: Downregulated PDIA3P1 lncRNA Impairs Trophoblast Phenotype by Regulating Snail and SFRP1 in PE
Source: Anal Cell Pathol (Amst). 2024 Apr 27;2024:8972022. doi: 10.1155/2024/8972022 (PMC11074859; doi:10.1155/2024/8972022)
Supplement: Supplementary 7 — Figure S1: ethynyl deoxyuridine (EdU) immunostaining confirmed that PDIA3P1 overexpression significantly promoted HTR-8/SVneo cells proliferation. Figure S2: EdU assay confirmed that PDIA3P1 overexpression significantly promoted JAR cell proliferation. Figure S3: the overexpression of PDIA3P1 in HTR-8/SVneo and JAR cells resulted in a decrease in the percentage of G0 phase of the cell cycle. Figure S4: SFRP1 expression was silenced in HTR-8/SVneo and JAR cells more efficiently by si-SFRP1-1# and si-SFRP1-2# by analyzing quantitative RT-PCR data. Data are reported as mean ± SD. ∗p < 0.05. [file 8972022.f7.docx]

SUPPLEMENTARY FIGURES


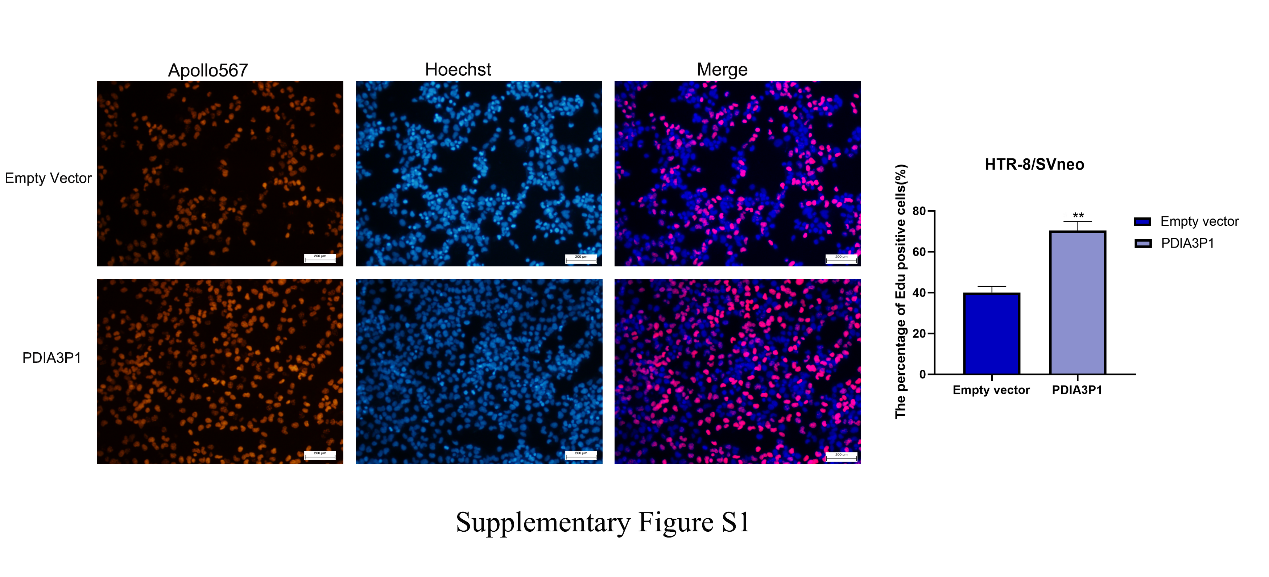


Supplementary Figure 1. Ethynyl deoxyuridine (EdU) immunostaining confirmed that *PDIA3P1* overexpression significantly promoted HTR-8/SVneo cells proliferation.


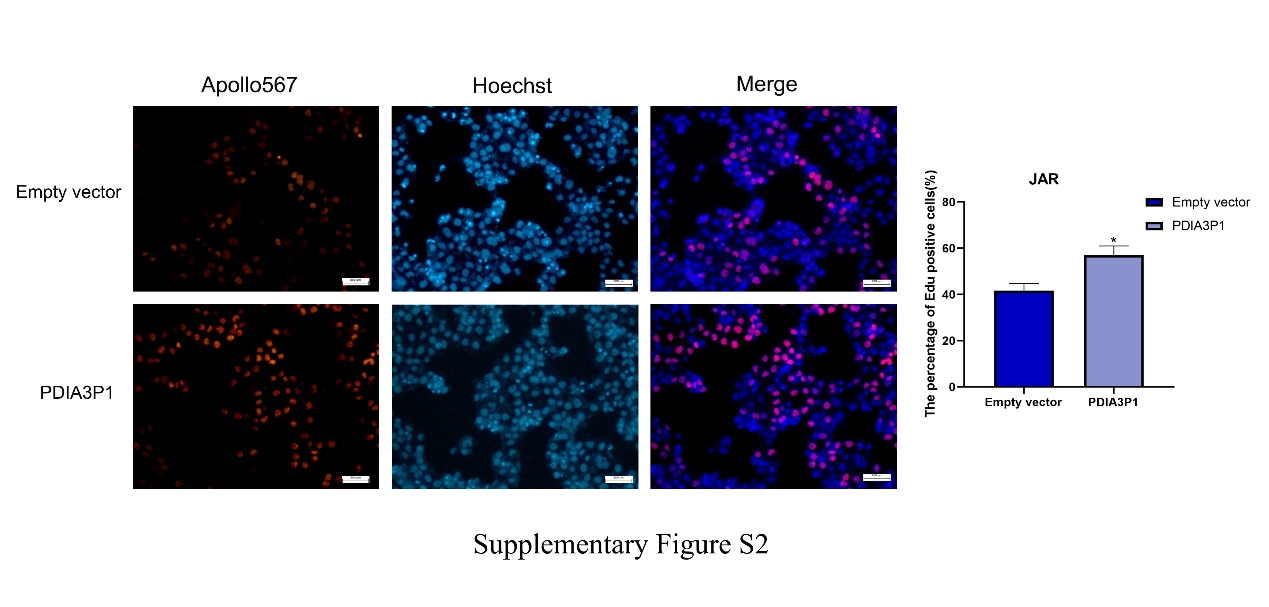


Supplementary Figure 2. EdU assay confirmed that *PDIA3P1* overexpression significantly promoted JAR cells proliferation.


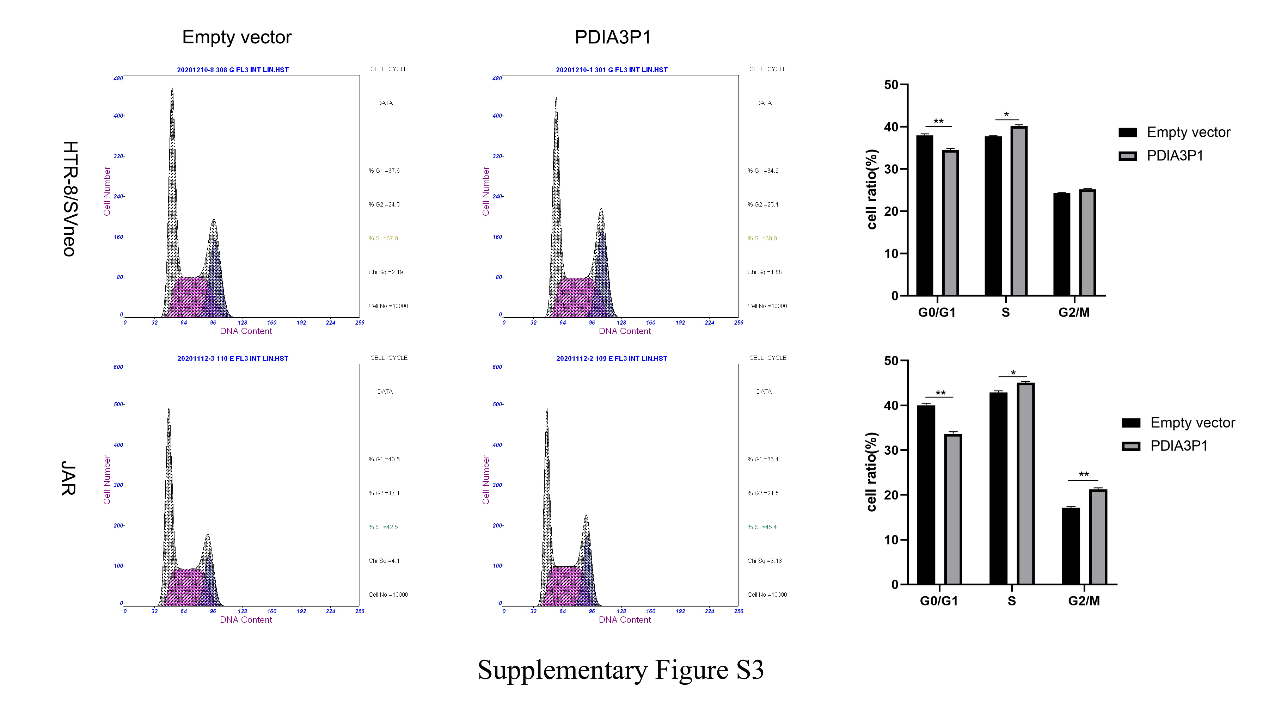


Supplementary Figure 3. The overexpression of *PDIA3P1* in HTR-8/SVneo and JAR cells resulted in a decrease in the percentage of G0 phase of the cell cycle.


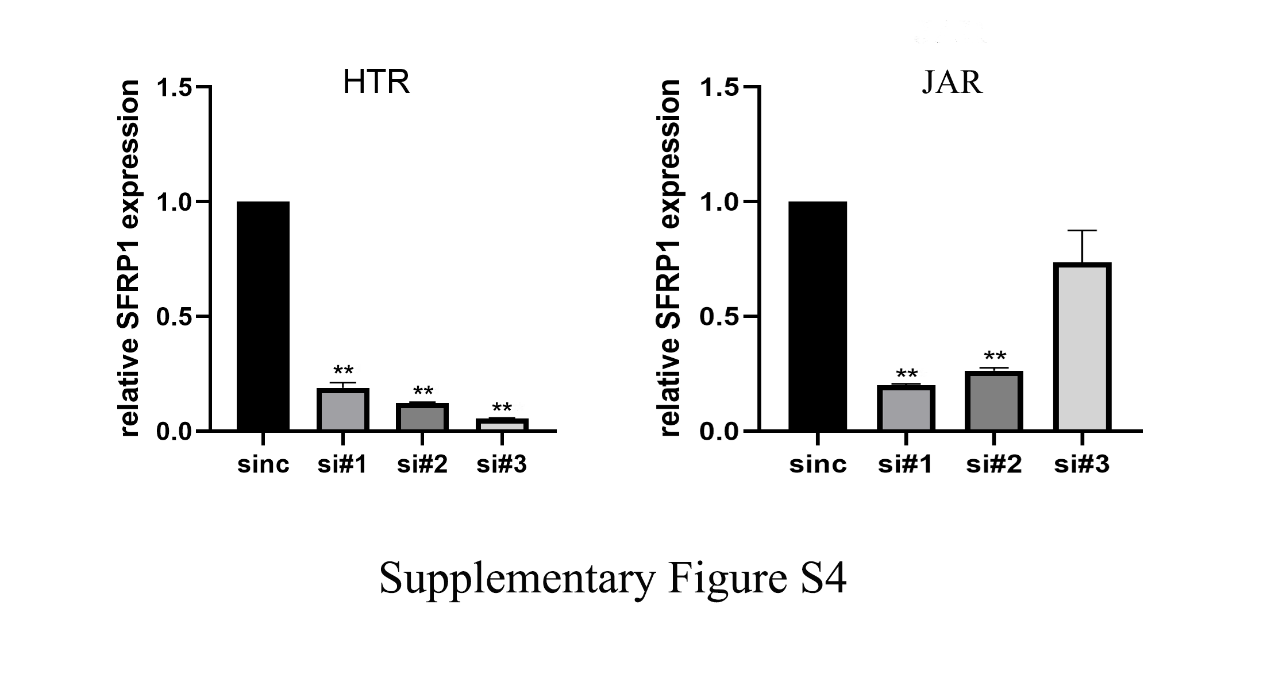


Supplementary Figure 4. SFRP1 expression was silenced in HTR/SVneo and JAR cells more efficiently by si-SFRP1-1# and si-SFRP1-2# by analyzing quantitative real-time PCR data.


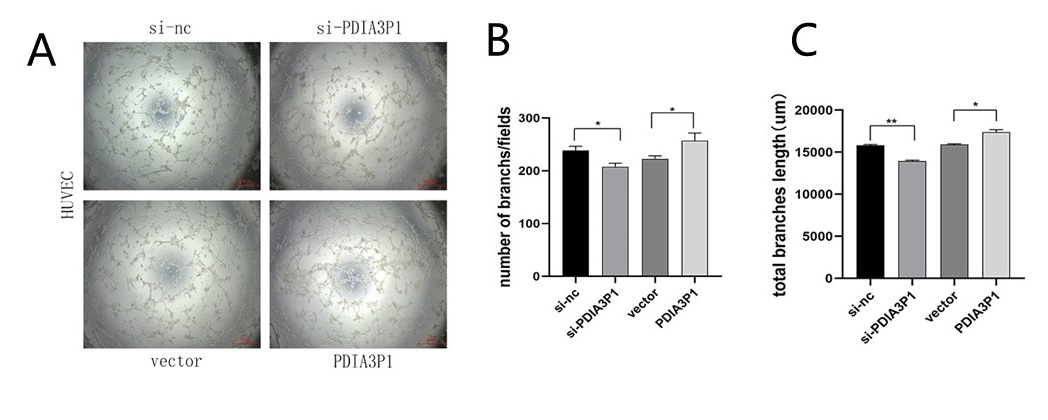


Supplementary Figure 5. *PDIA3P1* expression was silenced or overexpressed in HUVECs. Performing network formation, cells transfected with siRNAs/overexpressed plasmid targeting *PDIA3P1* showed a decrease/increase in node numbers as compared to the negative control. (A)Tube formation images of HUVEC cells transfected with si*PDIA3P1* or *PDIA3P1* plasmid were photographed. The number of branches (B) and total branch length (C) was analyzed through tube formation assays. Data are reported as mean ± SD. *p < 0.05; **p < 0.01.
